# Leveraging open cheminformatics tools for non-targeted metabolomics analysis of *C. elegans*: a workflow comparison and application to strains related to xenobiotic metabolism and neurodegeneration

**DOI:** 10.1007/s00216-025-06048-y

**Published:** 2025-08-08

**Authors:** Gianfranco Frigerio, Yunjia Lai, Emma L. Schymanski, Gary W. Miller

**Affiliations:** 1 Luxembourg Centre for Systems Biomedicine (LCSB), University of Luxembourg, 6, Avenue du Swing, L-4367 Belvaux, Luxembourg; 2 Center for Omics Sciences (COSR), IRCCS San Raffaele Scientific Institute, Milan, Italy; 3 Department of Environmental Health Sciences, Mailman School of Public Health at Columbia University, New York, NY USA

**Keywords:** Untargeted metabolomics, Exposomics, CYP enzyme mutant, FMO enzyme mutant, SV2C expression, Tau aggregation

## Abstract

*Caenorhabditis* *elegans* (*C. elegans*) is a well-established nematode model for studying metabolism and neurodegenerative disorders, such as Alzheimer’s (AD) and Parkinson’s disease (PD). Non-targeted metabolomics via liquid chromatography coupled with tandem mass spectrometry (LC–MS/MS) has proven useful for uncovering metabolic changes in biological systems. Here, we present workflows for *C. elegans* metabolomics, leveraging advanced open science tools. We compared two metabolite extraction methods: a monophasic extraction, which provided broader metabolite coverage in analyses conducted in hydrophilic interaction with positive polarity (HILIC POS), and a biphasic extraction, which yielded more features in reverse-phase C18 chromatography with negative polarity (RPLC NEG) analyses. Data were processed using patRoon, integrating IPO, XCMS, CAMERA, and MetFrag, which incorporated PubChemLite compounds and *C. elegans*–specific metabolites from an expanded WormJam database enhanced with PubChem and literature sources. MS-DIAL was also employed for data processing, allowing for expanded annotations with predicted spectra for the expanded WormJam metabolites calculated using CFM-ID. Significant metabolite differences were identified when comparing the Bristol (N2) wild-type strain with two knockout strains of xenobiotic-metabolizing enzymes and two transgenic strains related to neurodegenerative pathways. Pooled quality control (QC) samples for each strain ensured robust data quality and the detection of strain-related metabolites. Our study demonstrates the potential of non-targeted metabolomics for metabolite discovery employing open science tools in model organisms.

**Supplementary Information:**

The online version contains supplementary material available at 10.1007/s00216-025-06048-y.

## Introduction

Alzheimer’s disease (AD) and Parkinson’s disease (PD) are the most prevalent neurodegenerative diseases, affecting millions worldwide, characterized by complex biological mechanisms [1]. The aetiology involves an interplay of genetic and environmental factors, with a prolonged latency between initial triggers and symptom onset [2, 3]. Understanding the biological mechanisms underlying these diseases is crucial for developing therapeutic interventions. However, human studies are constrained by prolonged disease progression and limited access to relevant sample material, such as brain tissues and cerebrospinal fluid (CSF). Model organisms offer valuable alternatives for investigating mechanisms of neuronal injury and death as many of the processes are conserved across species. The nematode *Caenorhabditis elegans* (*C. elegans*) is a robust model for studying neurodegeneration [4]. Its advantages include a short lifespan, which enables rapid experimental manipulations and observations [5], and a well-characterized genome, which shares approximately 60–80% homology with human genes [6–8]. Moreover, the fully mapped nervous system contains only 302 neurons, which reduces complexity, enhances experimental precision, and retains disease relevance [4].

Mutant *C. elegans* strains are increasingly accessible for modeling neurodegenerative disease progression and related environmental factors. Wild-type strains serve as baselines for understanding biological processes under physiological conditions. Knockout strains targeting xenobiotic-metabolizing enzymes—such as mutations affecting cytochrome P450 expression [9] or multidomain flavoprotein monooxygenase (FMO) activity [10]—enable investigations into chemical metabolism. Transgenic strains modeling aspects of PD and AD, such as those overexpressing tyrosine hydroxylase in dopaminergic neurons (PD-related) [11, 12] or pro-aggregation tau fragments (AD-related) [13], provide insights into the molecular mechanisms underlying these disorders.

Genomics assesses the genes that encode the proteins and enzymes that process biochemicals, but metabolomics actually measures what is actually happening within an organism, providing direct insights into the phenotype [14–16]. The WormJam consensus model, established through community collaboration, integrated and curated multiple metabolic network reconstructions, offering an extensive data source of *C. elegans*–specific metabolic pathways and metabolites to characterize the metabolome [17]. In parallel, PubChem’s taxonomy pages compile species-specific data, including those for *C. elegans* [18, 19]. Moreover, a list of metabolites related to *C. elegans* was curated from a literature review [20]. These rapid research advancements underscore the need for open source, collaborative workflows to ensure continuous updates and knowledge sharing.

Investigating metabolomic alterations in disease-related transgenic strains is of particular interest for understanding the disease-related biochemistry. To accomplish this, metabolomics strategies are broadly categorized into *targeted* approaches, which focus on accurate and sensitive quantification of a known set of metabolites, and *non-targeted* approaches, which aim to detect and screen as many metabolites as possible. While non-targeted metabolomics offers broad exploratory potential, it presents challenges across the workflow, from sample preparation to compound annotation—the latter being a primary bottleneck in non-targeted workflows [21]. Various extraction methods have been applied to maximize metabolite recovery and coverage from *C. elegans* in non-targeted studies. Monophasic solvent extraction ranged from methanol [22–28], acetonitrile [29], or a combination of both [30, 31]. Biphasic extraction methods have also been used to separate polar and non-polar compounds. Traditional protocols from Folch and coworkers and from Bligh and Dyer utilize chloroform, methanol, and water for lipid analyses [25, 32–35]. More recently, the Matyash method, substituting chloroform with methyl-tert-butyl-ether (MTBE) [26, 36], has gained traction. After the addition of the organic solvent, a centrifugation step is typically performed, and only the supernatant is retained for analysis in order to avoid injecting proteins and other interfering matrix components [34]. For instrumental analysis, liquid chromatography (LC) coupled with high-resolution tandem mass spectrometry (LC–MS/MS) remains one of the preferred analytical approaches due to its versatility in accommodating various chromatographic conditions—reversed phase (RPLC) and hydrophilic interaction (HILIC)—and mass spectrometry polarity modes (positive and negative ionizations) [20].

After feature extraction and prioritization is performed, the most challenging aspect of non-targeted metabolomics is data processing and compound annotation [21]. To address this, open source tools have been developed [37–39]. Compared to vendor software, open source solutions offer advantages such as full transparency of the underlying code and algorithms. Among them, patRoon is an R package that streamlines the use of multiple established algorithms for non-targeted data processing, although primarily with an environmental focus and increasingly applicable to metabolomics [40, 41]. MS-DIAL is another widely implemented tool, which specializes in metabolomics and lipidomics analyses, visualization, and interpretation [42]. Metabolite annotation typically involves matching experimental mass fragmentation patterns against spectral libraries of known compounds or against fragmentation patterns predicted from in silico fragmentation simulations, usually produced from a list of chemicals retrieved via mass or formula from compound databases. Information from the full scan data (accurate mass, isotopic pattern, adduct presence) can also be used to refine candidate selection. For the compound annotation, the source of candidate compounds plays an important role: MS-DIAL encompasses a large spectral library containing spectra of more than 15,000 unique molecules (MSP), including spectral information from both authentic standards and in silico predictions [43]. PubChem, a comprehensive open chemistry database, contains > 121 million small molecules [18]. However, considering all these molecules can lead to irrelevant annotations and unnecessary computational burden. PubChemLite was developed as an exposomics-relevant subset of currently over 450,000 compounds from PubChem, optimizing candidate selection for chemical annotation [44, 45], and has been integrated into workflows such as patRoon. Further refinement is possible by incorporating organism-specific resources, such as *C. elegans*–specific metabolite databases.

Taken together, the primary aim of this work was to establish a comprehensive experimental and data processing pipeline for *C. elegans* metabolomics. This pipeline leverages state-of-the-art open source cheminformatics tools, such as MS-DIAL and patRoon, to optimize metabolite extraction, data processing, and annotation. As a key component of the workflow, we expanded the WormJam consensus model established by Witting et al. [17] by integrating additional metabolites curated from the review of the literature [20] and with *C. elegans*–related metabolites from PubChem. Then, the workflow was designed to address the unique challenge of analyzing knockout strains deficient in xenobiotic-metabolizing enzymes and transgenic strains associated with neurodegenerative diseases. By integrating advanced analytical strategies with a comprehensive data interpretation framework, this study seeks to provide reference data for future studies aiming for enhanced accuracy and efficiency in characterizing the *C. elegans* metabolome, uncovering genome-exposome-biology interplay, and ultimately, resolving etiologic causes and disease mechanisms.

## Materials and methods

### *Caenorhabditis elegans*: strains, culturing, and synchronization

Five *C. elegans* strains were selected for their relevance in neurobiological and neurotoxicological research. These included wild-type Bristol (“N2”), serving as the baseline for physiological comparisons; two knockout strains of xenobiotic-metabolizing enzymes (“VC40”: CYP enzyme mutant, genotype *cyp-13A7(gk31) II*; and “VC1668”: a flavin-containing monooxygenase (FMO) enzyme mutant with the genotype *fmo-2(ok2147) IV*) [46]; two transgenic strains related to neurodegeneration (“UA57”: PD-related, expresses the ortholog of tyrosine hydroxylase in dopamine neurons, genotype *baIs4 [dat-1p::GFP* + *dat-1p::CAT-2]*; and “BR5270”: AD-related, pan-neuronal overexpression of the pro-aggregation fragment of human Tau, Model of AD, severe tauopathies, genotype: *byIs161 [rab-3p::F3(delta)K280* + *myo-2p::mCherry]*).

Worms were grown at 20 °C on NGM agar plates seeded with UV-treated *Escherichia coli* OP50 (Stiernagle, 2006). After ~ 5 days, gravid worms (post-L4 stage) were synchronized via hypochlorite bleaching. For each strain, worms were washed off four plates with 4 mL sterile M9 buffer per plate, pooled into a 15-mL conical tube, and allowed to settle for 5 min. The supernatant was removed, and ~ 2.5 mL of worm suspension was split into two 1.5-mL Eppendorf tubes. After centrifugation (max speed, 1 min), the supernatant was discarded, and 1 mL of bleach solution was added. Tubes were vortexed (30 s) and monitored under a dissecting microscope until only eggs remained. Eggs were pelleted by centrifugation (21,000 × *g*, 1 min), washed three times with M9 buffer to remove bleach, and resuspended in 100 µL M9. The suspension was pipetted onto a seeded 10-cm NGM plate, left to dry under a biosafety cabinet, and incubated at 20 °C for hatching. Hatched worms were considered synchronized.

Hatched L1 worms were cultured at 20 °C for 48 h until the majority reached the L4 larval stage (young adult with discernible vulva). Worms were then washed from the NGM plates with M9 buffer and subjected to flow cytometric sorting using a COPAS Flow Pilot FP-250 system (Union Biometrica, MA, USA). L4-stage worms were sorted based on an established gating strategy [47] and collected into 300 worms per tube. For each strain, corresponding M9 blanks were prepared by collecting the supernatant after one-minute centrifugation of the worm suspension. Both sorted worms and M9 blanks were snap-frozen and stored at − 80 °C.

### Metabolite extraction

Two metabolite extraction schemes were independently applied to all strains. Scheme 1 utilized a monophasic protocol [29, 48, 49], while Scheme 2 followed a biphasic approach [36, 50].

#### Scheme 1

For each strain, four worm replicates and duplicate M9 blanks were extracted, along with pooled quality control (QC) and method blanks (from 100 µL LC–MS grade water). Frozen samples were thawed on ice and reduced to 100 µL by centrifugation (380 × *g*, 1 min) with 200 µL of the M9 supernatant removed. Zirconium oxide beads (~ 20) were added, followed by 200 µL of ice-cold acetonitrile containing internal standards (SPLASH® LIPIDOMIX®, Avanti Research), achieving a 2:1 (v/v) acetonitrile:aqueous ratio. Samples were vortexed (30 s), homogenized on a bead beater (5 min, max speed, air-cooled), and equilibrated at − 20 °C for 30 min. After centrifugation (15,000 × *g*, 10 min), supernatants were split into 100 µL raw extracts and 100 µL dried extracts (via SpeedVac). QC pools were prepared by combining 50 µL from each replicate (total 200 µL), then split into raw and dried aliquots (100 µL each).

#### Scheme 2

Similarly, four worm replicates and duplicate M9 blanks were extracted per strain, along with QC and method blanks. Samples were thawed on ice, reduced to 100 µL (380 × *g*, 1 min), and combined with zirconium oxide beads (~ 20). Each tube was added with 225 µL of ice-cold methanol containing internal standards (SPLASH® LIPIDOMIX® Mass Spec Standard, Avanti Research), followed by vortexing (10 s). Subsequently, 750 µL of methyl tert-butyl ether (MTBE) was added, and samples were homogenized (10 min, max speed, air-cooled). After adding 188 µL of LC–MS grade water and vortexing (20 s), samples were centrifuged (14,000 × *g*, 2 min) to separate phases. A total of 250 µL of the upper (MTBE:methanol) phase and 100 µL of the lower (methanol:water) phase were collected. QC pools were prepared by combining 100 µL from each replicate for the upper phase (400 µL total) and 50 µL from each for the lower phase (200 µL total). Each pool was split equally into raw and dried extracts (e.g., 200 µL raw and dried for upper; 100 µL raw and dried for lower). All samples (scheme 1 and scheme 2, raw and dried) were shipped from Columbia University (USA) to the University of Luxembourg on dry ice with a World Courier. In the present paper, results are reported only for the dry samples, while analyses also on raw samples are reported in the external repository [51].

### LC–MS/MS analyses

Dried scheme 1 samples and dried scheme 2 bottom-phase samples were resuspended in 100 µL of acetonitrile:water solution (50:50), while scheme 2 dried upper phase samples were resuspended in 100 µL of acetonitrile. All samples were vortexed for 30 s and transferred to autosampler vials for analysis.

Instrumental analyses followed previously published methods with modifications: chromatographic separation was similar to the method of Blazenovic et al. [52] chosen to balance metabolite coverage with analytical throughput: Mass spectrometry conditions were adapted from the method of Talavera Andújar et al., optimized for use with the same instrument [53]. Samples were analyzed using reverse-phase liquid chromatography with negative polarity mode (RPLC NEG) and hydrophilic interaction liquid chromatography with positive mode (HILIC POS). As a system suitability check, internal standards were monitored daily to assess mass accuracy, peak shape, and retention time consistency throughout the analytical sequence. All solvents used for the mobile phases were LC–MS grade. For RPLC NEG, 5 µL of each sample was injected into the HPLC system (Thermo Scientific Accela LC system) equipped with a Waters Acquity CSH C18 column (100 × 2.1 mm, 1.7 µm) and an Acquity CSH C18 VanGuard precolumn (5 × 2.1 mm, 1.7 µm). Metabolites were separated with a linear gradient consisting of two mobile phases: the A phase was a solution of 40% water and 60% acetonitrile containing 10 mM ammonium acetate, while the B phase was a solution of 90% isopropanol and 10% acetonitrile containing 10 mM ammonium acetate. While Blazenovic et al. [52] employed ammonium formate as a mobile phase modifier, we used ammonium acetate to achieve a slightly higher pH, thereby improving the ionization efficiency of acidic compounds in the negative ionization mode. A constant flow rate of 400 µL/min was applied, and the percentage of the B phase changed as follows: from 15 to 30% between 0 to 2 min; 30 to 48%, 2 to 2.5 min; 48 to 82%, 2.5 to 11 min; 82 to 99%, 11 to 11.5 min; held constant at 99%, 11.5 to 12 min, then from 99 to 15%, 12 to 12.1 min; held constant at 15%, 12.1 to 15 min. The autosampler was kept at 4 °C, while the column was kept at 65 °C. After the chromatographic separation, the flow was delivered to a mass spectrometer (Q Exactive™ HF, Thermo Scientific) operating in electrospray ionization (ESI) negative mode; for each scan cycle, a full mass scan was carried out (scan range from 60 to 900 m/z; resolution 120,000; AGC target 1e6, Maximum IT 70 ms) followed by a top 10 data-dependent MS^2^ experiment (isolation window: 1 m/z, collision energy: 30 V, resolution 30,000, dynamic exclusion 10 s, AGC target 1e5, Maximum IT 70 ms). For HILIC POS, 5 µL of each sample was injected onto a Waters Acquity BEH Amide column (150 × 2.1 mm, 1.7 µm), equipped also with an Acquity BEH Amide VanGuard precolumn (5 × 2.1 mm, 1.7 µm). The A phase was a solution of 10 mM ammonium formate and 0.125% formic acid in water, while the B phase was a solution of 10 mM ammonium formate and 0.125% formic acid in water:acetonitrile (5:95). A linear gradient at a constant flow rate of 400 µL/min was run with the following B phase percentages: constant 100%, from 0 to 2 min; 100 to 70%, 2 to 7.7 min; 70 to 40%, 7.7 to 9.5 min; 40 to 30%, 9.5 to 10.25 min; 30 to 100%, 10.25 to 12.75 min; constant 100%, 12.75 to 17 min. The autosampler was kept at 4 °C while the column was kept at 45 °C. The mass spectrometer operated in ESI positive mode; for each scan cycle, a full mass scan was carried out (scan range from 60 to 900 m/z; resolution 60,000; AGC target 1e6, Maximum IT 70 ms) followed by a top 5 data-dependent MS^2^ experiment (isolation window: 1 m/z, collision energy: 20 V, resolution 30,000, dynamic exclusion 10 s, AGC target 1e5, Maximum IT 70 ms). The mass spectrometry settings for the HILIC analyses (lower resolution and fewer MS^2^ spectra compared to the RPLC ones) were adjusted to compensate for the typically narrower peaks observed with the HILIC method.

Scheme 1 samples were analyzed both with the RPLC NEG and HILIC POS methods, while scheme 2 upper phase samples were analyzed only with the RPLC NEG method, and scheme 2 bottom-phase samples were analyzed only with the HILIC POS method.

### Data processing

Raw files were converted to the open mzML format using MSConvertGUI (ProteoWizard, version: 3.0.22108-f83e548) [54], with peak-picking filter, vendor algorithm, and all MS levels enabled. These files were then processed both with patRoon (version 2.3.1) [40, 41] and MS-DIAL (version 5.1.230912) [42]. Data analysis in R (version 4.3.1) [55] with the RStudio interface (2023.06.1) utilized the tidyverse package (version 2.0.0) [56]. The patRoon and MS-DIAL parameters were chosen to best suit each software and to ensure comparability between the two. Notably, gap filling was not performed during feature extraction in both patRoon and MS-DIAL to assess the actual feature presence across QC samples and incorporate this information as part of the quality control procedure, as detailed in the following sections.

A custom *C. elegans* metabolite collection, termed “WormJam expanded” hereafter, was compiled from the WormJam database [17], PubChem *C. elegans*–related metabolites [19] and literature-sourced metabolites [20], as documented in an RMarkdown file [57].

#### patRoon

The following data analyses were carried out using different functions from the patRoon R-package. Specifically, the features, i.e., signals with a defined *m*/*z* ratio and retention time, were extracted and aligned using the “xcms3” algorithm [58–60]. The XCMS parameters were selected by running the IPO algorithm [61] on pooled quality control samples; the componentization, i.e., grouping features likely originated from or related to the same molecule by isotope patterns and adducts, was conducted using the CAMERA algorithm [62]. Then, after retrieving the MS/MS peak list within patRoon, annotation was performed with MetFrag [63] considering two databases separately: PubChemLite (version 1.19.0) [45, 64] and the “WormJam expanded” collection [57]. Details of all the parameters and processing workflow used can be found in the R-scripts available in a publicly available GitHub repository [65].

#### MS-DIAL

The parameters used for data processing in MS-DIAL (version 5.1.230912) were ionization type: soft ionization, separation type: chromatography, MS method type: conventional LC/MS or data-dependent, collision type: CID/HCD, data type: centroid data, target omics lipidomics (for RPLC NEG) and metabolomics (for HILIC POS), MS1 accuracy tolerance: 0.02 Da, MS2 accuracy tolerance: 0.025 Da, minimum peak height: 1000 amplitude, mass slice width: 0.1 Da. For annotation, the MSMS_Public_EXP_NEG_VS17 and the MSMS_Public_ExpBioInsilico_NEG_VS17 (in negative) and the MSMS_Public_EXP_POS_VS17 (in positive) MSP libraries were used, MS1 accurate mass tolerance: 0.02 Da, MS2 accurate mass tolerance: 0.05 Da, dot product score cut off: 50, weighted dot product score cut off: 100, reverse dot product score cut off: 100, matched spectrum percentage: 0%, minimum number of matched spectrum: 1. The adducts considered were [M-H]^−^, [M-H_2_O-H]^−^, [M + Na-2H]^−^, [M + HCOO-H]^−^, [M + CH_3_COO-H]^−^, [M + CH_3_COONa-H]^−^ (in negative) and [M + H]^+^, [M + NH_4_]^+^, [M + Na]^+^, [M + CH_3_OH + H]^+^, [M + K]^+^, [M + ACN + H]^+^, [M + H-H2O]^+^ (in positive). Analyses were aligned against a pooled quality control file, with retention time tolerance of 0.1 min, MS1 tolerance of 0.02 Da, retention time, and MS1 factor of 0.5. Manual curation, based on a visual inspection of peak shape, was performed for the significant compounds before exporting the area intensity table, which was used for further elaboration and statistical analyses.

Additionally, the MS-DIAL processing was repeated with the same parameters, using MSP libraries created by in silico fragmentation of the compounds in the WormJam expanded database. The function “cfm-predict” of the competitive fragmentation modeling tool CFM-ID [66–69] was used through Docker Desktop to predict mass spectral fragments from the SMILES of the WormJam expanded compounds. The in silico fragmentation was performed separately in positive and negative modes, each considering three collision energies (low or “energy0,” 10 V; medium or “energy1,” 20 V; high or “energy2,” 40 V). Intensity weights were applied to combine fragments obtained with the three collision energies: energy0, enegy1, and energy2 were weighted 10%, 80%, and 10% in positive (since HILIC POS analyses were carried out with a collision energy of 20 V); and 10%, 45%, and 45% in negative (since collision energy in RPLC NEG were 30 V) modes, respectively. The resulting positive and negative MSP libraries were exported and used for the MS-DIAL re-analysis. The code created to perform this elaboration is publicly available [65].

#### Feature processing, statistical, and pathway analysis

**Fig. 1:**
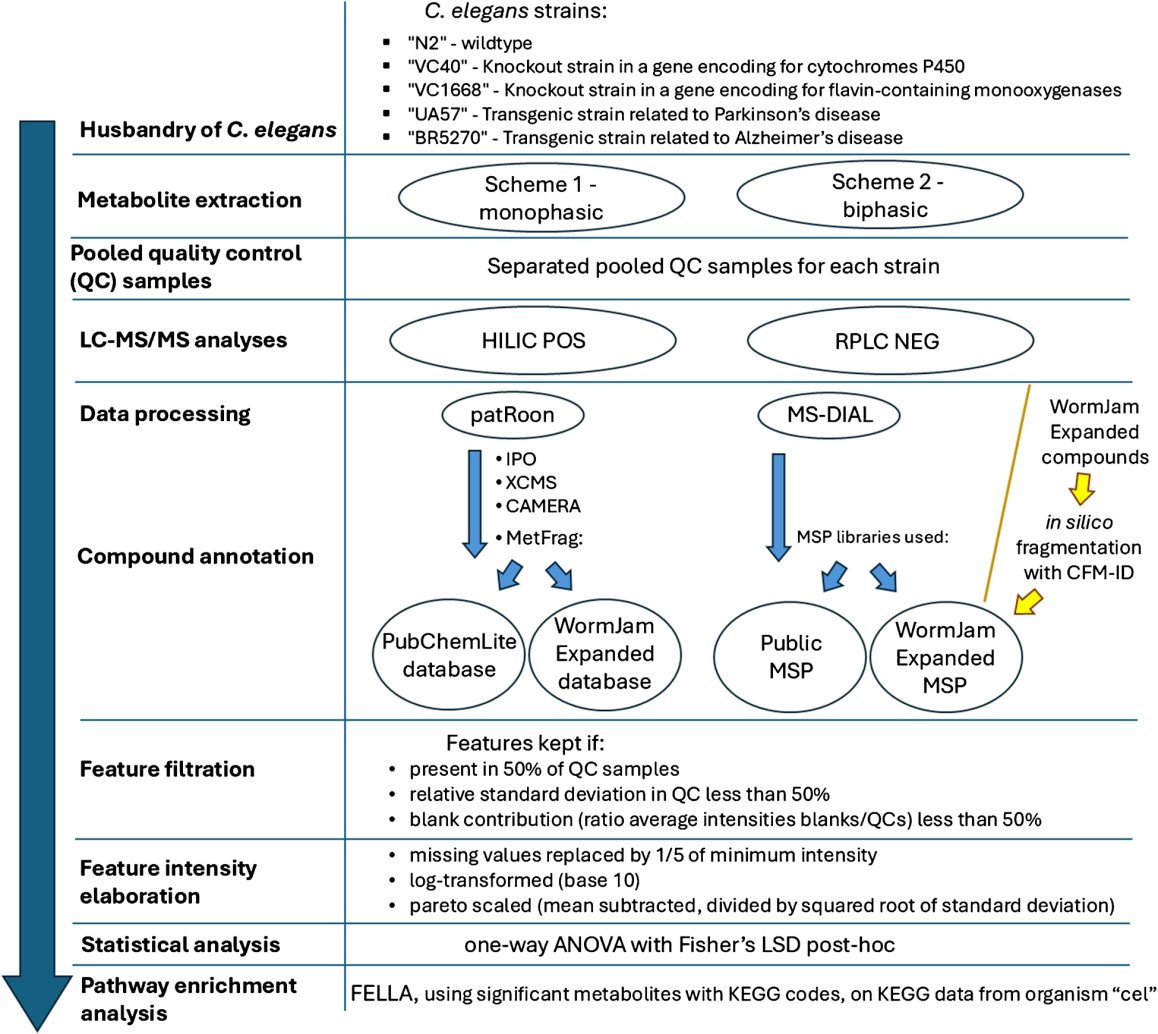
An overview of the experimental and data analysis workflow, as detailed in the “Materials and methods” section. Circles represent steps performed in parallel, with results subsequently compared for comprehensive analysis

A summary of all the experimental and data analyses workflow is shown in Fig. 1, while all the R-scripts written to perform all the data elaborations, statistical analyses, visualizations, and pathway analyses are publicly available [65]. The annotated compounds were ranked according to the confidence levels by Schymanski et al. [70] in particular using MoNAScore cut-offs in patRoon, and dot product and fragment presence cut-offs in MS-DIAL, as previously reported by Talavera et al. (“2a,” “2b,” “3a,” “3b,” “3c,” “4a,” “5”) [53]. Every feature table was filtered considering the pooled quality controls: in particular, separately for each type of pooled QC sample, features were retained only if they were detected in at least 50% of QCs, had a relative standard deviation (RSD) below 50% in QC intensities, and showed a blank contribution (the ratio of average intensity in blanks and QCs) less than 50%. Then, every feature that passed this check in at least one QC sample type was retained and considered for the following statistical analyses [71]. Data intensities from each feature that passed the pooled QC check were transformed as follows: missing values were replaced by 1/5 of the minimum intensity, then they were log-transformed (base 10) and Pareto-scaled (mean subtracted and divided by the squared root of the standard deviation). Afterwards, one-way ANOVA was performed on transformed intensities for each feature, considering the five sample groups (N2, VC40, VC1668, UA57, and BR5270); pairwise group comparison was performed with the Fisher’s LSD post hoc test implementing the agricole package [72]; false discovery rate (FDR) correction was also applied on the ANOVA *p*-values [73], and an FDR *p*-value less than 0.05 was considered statistically significant. Separate statistical analyses were conducted for each extraction procedure, for both RPLC NEG and HILIC POS.

For the annotated features, additional chemical information was retrieved using the R-packages “RChemMass” [74], “webchem” [75], “classyfireR” [76], and “metaboliteIDmapping” [77]. Euler-Venn diagrams were created with the “eulerr” package [78] and the “ggven” package [79] to compare the features among the different sample extraction procedures; similarly, an upset plot was also built using the “UpSetR” package [80]. SankeyNetwork graphs, with the “networkD3” package [81], were built to visualize the different categories of annotated compounds. A dedicated graph was created to visualize the presence of annotated compounds among the different procedures and to visualize which of those were statistically significantly different from the control group, using the packages “ggplot” [56], “patchwork” [82], “grid,” and “gridExtra” [83]. For each compound, the KEGG code was assigned using metaboliteIDmapping [77] and, if there was no correspondence, it was manually retrieved from the PubChem page of that molecule, if present; then, considering statistically significant metabolites for which a KEGG code was found, a pathway enrichment analysis was performed with the “FELLA” R-package [84], after building the KEGG knowledge model from the “cel” (*C. elegans*, nematode) organism.

## Results and discussion

### Sample preparation schemes

**Fig. 2:**
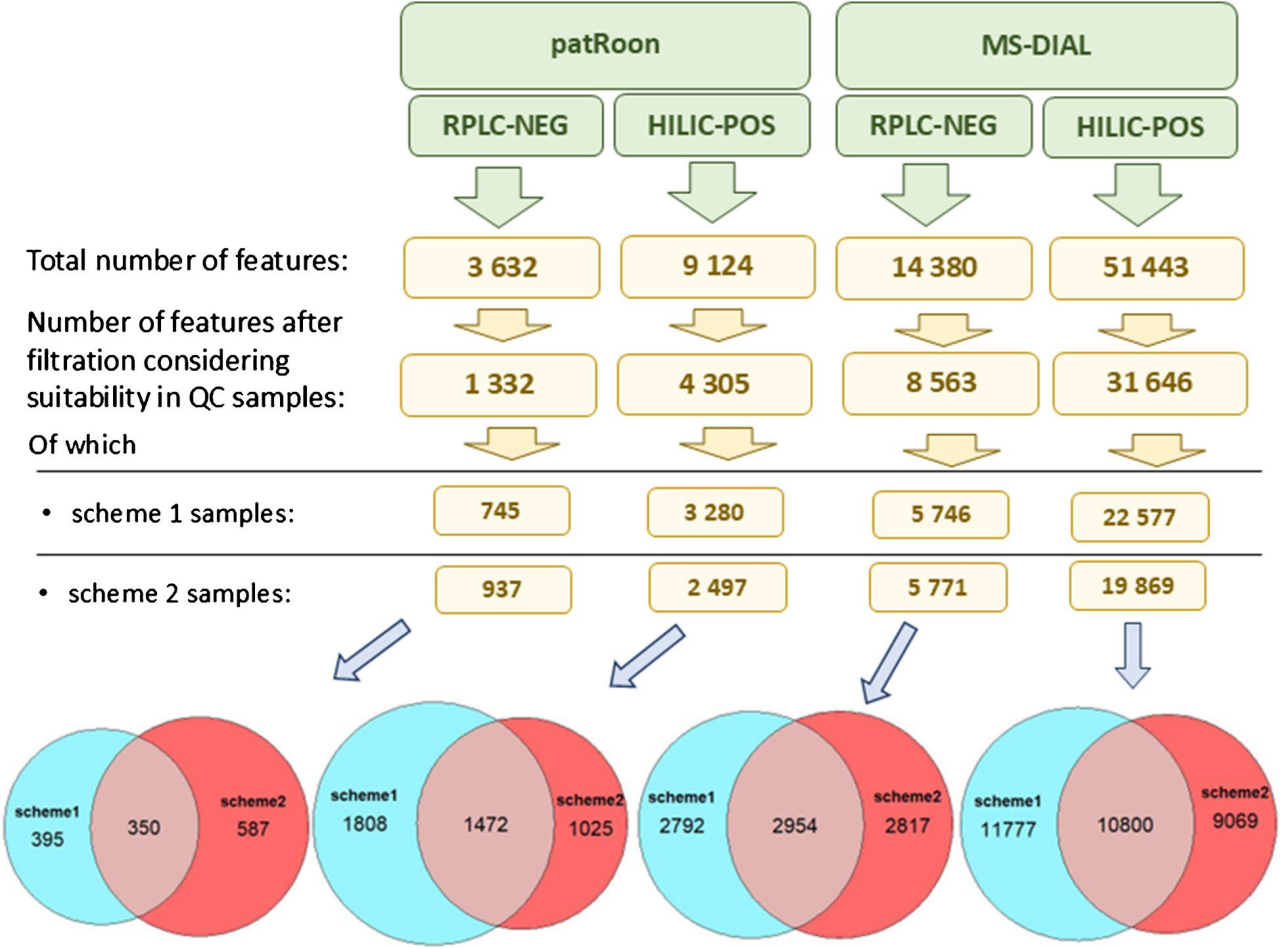
Summary of feature counts obtained across different analytical setups—RPLC NEG and HILIC POS—using either patRoon or MS-DIAL. Initial feature counts were refined by applying pooled QC filtering. The lower section shows the number of features passing the QC check for each sample preparation scheme, while Euler-Venn diagrams depict feature overlaps among the schemes. The details of the QC check procedure are reported in the “Feature processing, statistical, and pathway analysis” section

Considering the various data processing workflows, distinct feature tables were generated using patRoon and MS-DIAL for both RPLC NEG and HILIC POS analyses. Features were filtered based on their consistency in QC samples to retain only biologically plausible features. The number of features retained at each processing step, including the different sample preparation schemes, is summarized in Fig. 2.

Here, we compared the biologically plausible features across analytical steps. Among features that passed the pooled QC check, a higher number was detected in HILIC POS analyses (4305 with patRoon and 31,646 with MS-DIAL) compared with RPLC NEG (1332 using patRoon and 8563 with MS-DIAL). We hypothesize that the higher number of features detected in HILIC analyses is primarily attributable to the nature of the chromatography, as HILIC retains polar and hydrophilic small compounds better. Furthermore, the use of electrospray ionization in positive mode might also explain the higher number of features, as we hypothesize that it enables better ionization efficiency and broader metabolite coverage than in negative mod; this likely contributed to the greater sensitivity and feature detectability in HILIC POS analyses compared to RPLC NEG.

In terms of extraction schemes, differences emerged depending on the analytical setup and processing tool. For RPLC NEG analyses, the biphasic extraction (scheme 2) yielded a higher number of plausible features when processed with patRoon (937 features in scheme 2 compared to 745 in scheme 1), while feature counts were comparable in MS-DIAL (5771 and 5746). In contrast, HILIC POS analyses favored scheme 1: when processed with patRoon, scheme 1 resulted in 3280 features, surpassing scheme 2 with 2497 features; similarly, MS-DIAL processing yielded 22,577 features for scheme 1 and 19,869 for scheme 2.

Although there was substantial overlap in features detected among extraction schemes, certain features were unique to specific methods. In patRoon-processed RPLC NEG data, scheme 2 had more unique features (587). Conversely, for HILIC POS analyses, scheme 1 exhibited greater uniqueness, with 1808 features exclusive to this extraction method. Overall, the results suggest that biphasic scheme 2 is more suitable for RPLC analyses, while monophasic scheme 1 performs better with HILIC analyses.

Our findings align with the observations of Geiger et al., who reported significant differences in feature counts across extraction strategies for *C. elegans* in RPLC NEG: Their results underscored the critical role of solvent choice, despite using different solvent combinations (80% methanol in water vs. chloroform/methanol) [25]. In contrast, a previous study that investigated the reproducibility and yield of tissue extraction procedures, although not in *C. elegans*, suggested monophasic extraction for the concurrent analysis of polar and non-polar metabolites [85]. Moleenars and coworkers developed and validated a simple biphasic extraction (using water:methanol:chloroform 1:1:2) for *C. elegans* metabolomics and lipidomics. Their comparison of lipidomics outcomes indicated that most lipids were consistently detected across methods, but some lipid subclasses displayed extraction-specific patterns. For instance, lysophosphatidylethanolamines were uniquely identified in monophasic extracts (using methanol:chloroform 1:1), while bis(monoacylglycero)phosphates and other low-abundance lipids were better captured using biphasic extraction [34].

Regarding instrumental method choice, although implementing all four combinations of chromatographic methods (RPLC and HILIC) and mass spectrometry polarities (negative and positive) maximizes metabolite coverage [86, 87], in this work, we combined RPLC NEG and HILIC POS only to balance throughput and metabolite coverage, following prior recommendations [88].

When comparing feature detection tools, MS-DIAL consistently detected a higher number of features than patRoon (XCMS algorithm), a trend also reported by [53]. However, Li et al. observed the opposite in their study, finding XCMS to yield both a higher number of features and verified “true positive” features compared to MS-DIAL [89]. These discrepancies highlight the importance of employing multiple data processing tools for non-targeted metabolomics, as their combined use and cross-reference can enhance biomarker discovery and reduce methodological bias [89].

### Compound annotation strategies

**Fig. 3:**
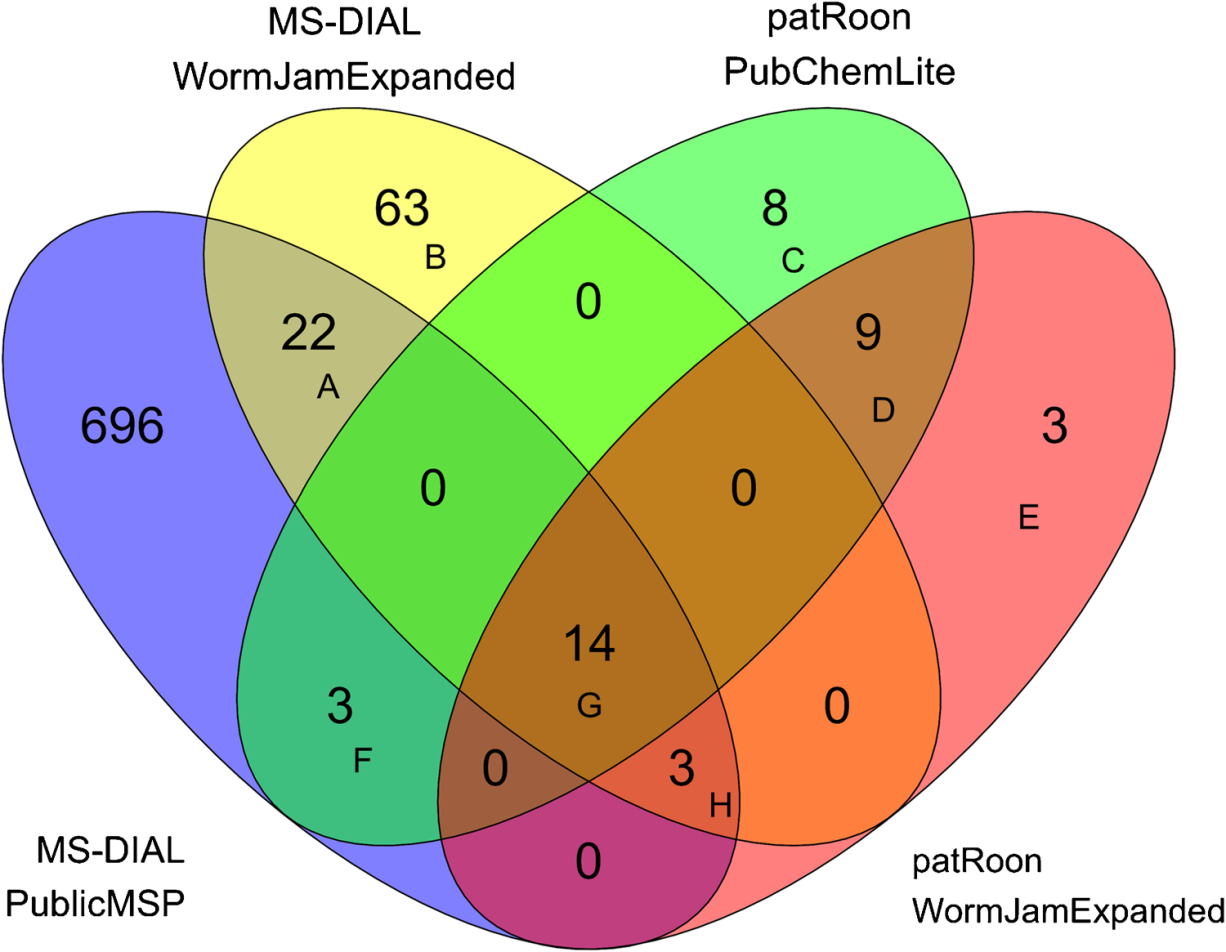
Euler-Venn diagram illustrating the unique compounds annotated at level 3 or above. The results integrate all sample preparation schemes and chromatographic runs and are grouped by the four annotation strategies applied, highlighting intersections among them. The compounds contained in the overlaps are (click embedded hyperlink): **A**: (22 compounds), **B**: (63 compounds), **C**: (8 compounds), **D**: (9 compounds), **E**: (3 compounds), **F**: (3 compounds), **G**: (14 compounds), **H**: (3 compounds)

Following feature and mass spectral processing with MS-DIAL and patRoon, extensive comparisons were conducted. The number of annotated features across chromatographic runs, sample preparation schemes, and annotation strategies—grouped by annotation levels—are detailed in Supplementary Figures S1–S13. Combining results from RPLC NEG and HILIC POS analyses across both sample preparation schemes, a total of 821 unique compounds were annotated at level 3 or above. Specifically, 738 compounds were uniquely annotated using MS-DIAL with the Public MSP, 102 with MS-DIAL and the WormJam MSP, 34 using patRoon with PubChemLite, and 29 with patRoon and WormJam. A visual summary of these results is presented in Fig. 3, with an accompanying upset plot provided in Supplementary Figure S14. Full details of the annotated compounds are available in Supplementary Table S1.

Supplementary Figure S15 presents a sankeyNetwork graph summarizing the distribution of annotated compound classes. According to ClassyFire classification, among compounds annotated at level 3 or above, 195 compounds (23.8%) belonged to the superclass of organoheterocyclic compounds, 164 (20.0%) were organic acids and derivatives—of which 111 were amino acids, peptides, or analogues—141 (17.2%) were lipids or lipid-like molecules, including 79 fatty acyls, and 106 (12.9%) were benzenoids. Since most compounds were annotated with MS-DIAL, a separate sankeyNetwork graph related only to the 40 compounds annotated with patRoon is also reported in Supplementary Figure S16. Among these, 13 compounds (32.5%) were organic acids and derivatives, including 10 amino acids and derivatives; 7 (17.5%) were nucleosides, nucleotides, and analogues; 6 (15.0%) were organoheterocyclic compounds; and 5 (12.5%) were lipids and lipid-like molecules, all classified as fatty acyls.

A larger number of unique annotations were found in MS-DIAL. MS/MS spectra and fragment matches were comparable between the ones produced by CDM-ID and MetFrag, and the discrepancies are likely caused by other factors: patRoon, originally developed for environmental analysis, tends to extract fewer features in complex biological samples like metabolomics datasets, applies stricter criteria for feature annotation, and tends to assign lower confidence levels compared to MS-DIAL, which may contribute to the lower number of high-confidence annotations retained.

While MS-DIAL yielded a substantially higher number of annotated compounds, some unlikely annotations were noted even at high confidence level (2a), such as methylpyrrolidine and desethylatrazine, or at a lower confidence level such as sulcatol (3a), chlormequat, melamine, nitrosodimethylamine, drofenine, wogorin, and diaveridine (3b): Many of these are exogenous compounds that are unlikely to be present in these samples. This may in part explain why so many more features were found using MS-DIAL with the public MSP libraries, and in future studies, further parameter refinement may be necessary to avoid so many false positives. However, employing in MS-DIAL the in silico MSP libraries generated from the WormJam expanded database improved annotation reliability: Although the number of compounds annotated using these in silico libraries was lower compared to public MSP libraries, unreliable annotations were notably absent, suggesting that dedicated MSP libraries can enhance the accuracy of candidate annotations in MS-DIAL. For patRoon, most annotated compounds overlapped between PubChemLite and WormJam expanded databases, although a few unique compounds were identified with PubChemLite. This highlights the complementary value of combining multiple annotation resources to increase the coverage. Restricting searches to organism-specific or highly curated spectral libraries—such as WormJam—enhances annotation quality, even if this strategy aligns more closely with a suspect screening approach and may limit the detection of novel or unexpected compounds. Conversely, broader searches using public or in silico libraries substantially expand chemical coverage but induce higher false-positive rates and computational demands. Our results highlight the risks and trade-off between annotation quality and coverage and underscore the value of hybrid strategies that integrate both specific and broad-spectrum resources to optimize metabolite annotation in non-targeted workflows.

The occurrence of apparently unlikely level 2a annotations highlights the trade-off of balancing sensitivity and specificity in MS^2^ spectral matching in non-targeted metabolomics: More permissive score thresholds and broader public spectral libraries, such as those used in MS-DIAL with the public MSP libraries, can improve coverage and reduce false negatives, but also increase the risk of false positives. Conversely, more conservative approaches like in patRoon yield fewer annotations but also fewer unlikely ones. These findings underscore the limitations of relying solely on MS^2^-based identification, as mass spectra are not always sufficiently informative. Moreover, in silico spectra may lack the validity of experimental reference spectra, and different scoring algorithms may prioritize different spectral features, leading to inconsistent or incorrect annotations. This shows that validation with authentic reference standards remains essential, and compound annotations should be considered a starting point for hypothesis generation rather than a definitive compound identification.

### Metabolite differences across *C. elegans* strains

ANOVA tests were performed to assess biological differences among the *C. elegans* strains. An overview of all statistically significant features, including their annotation levels, is given in Supplementary Figures S1–13, while detailed information on significantly different compounds annotated at level 3 or above is provided in Supplementary Table S2. In RPLC NEG analyses, more significant features were identified in samples extracted with scheme 1 compared to scheme 2 (105 vs. 71 with patRoon, and 182 vs. 98 with MS-DIAL). For HILIC POS analyses, significant feature counts were similar between schemes using patRoon (148 vs. 166), while MS-DIAL identified more significant features in scheme 1 (363 vs. 233). Considering all extraction schemes, analytical modes (RPLC NEG and HILIC POS), and data processing tools (patRoon and with MS-DIAL), a total of 87 unique compounds were significantly different among strains and annotated at least at level 3, as reported in Supplementary Table S2. The following section focuses on those 87 features.

**Fig. 4:**
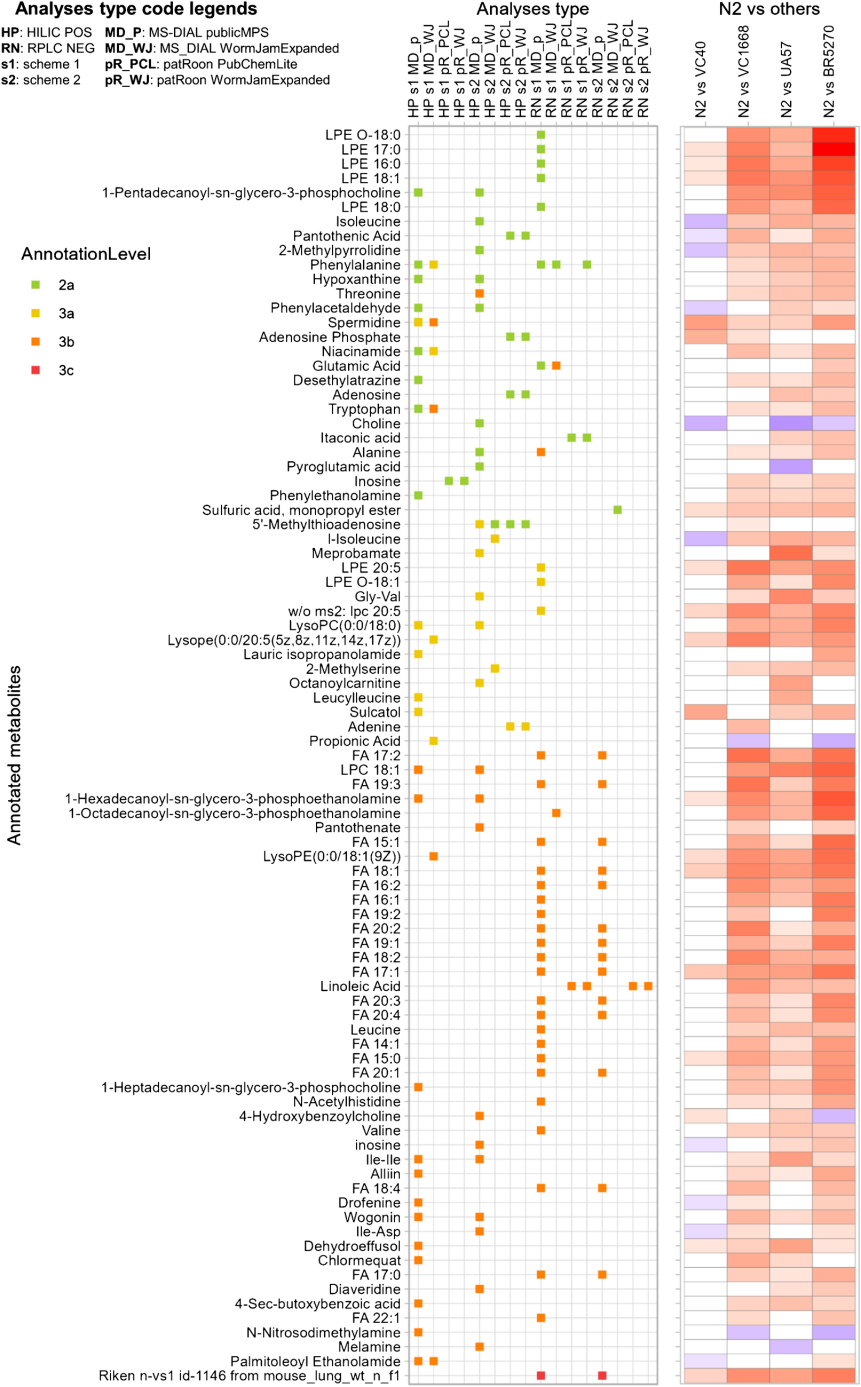
A visual summary of compounds annotated at level 3 or above that were significantly different among the analyzed groups. Compounds are sorted by FDR-corrected ANOVA *p*-values (lowest at the top). The central panel indicates the analysis types in which these compounds were detected and annotated, while the right-hand graph displays Fisher’s LSD pairwise comparisons between the wild-type group (N2) and other sample groups: white cells represent no significant difference, red indicates significantly higher intensity in N2, and blue denotes significantly lower intensity in N2, with deeper shades indicating stronger significance (lower *p*-value)

Among these 87 compounds, 28 were annotated at confidence level 2a, 15 at level 3a, 43 at level 3b, and 1 at level 3c. The overview of the different levels and related scores to reach them is reported in Table 1 of Talavera Andújar et al. [53]. According to ClassyFire classification, 24 compounds were fatty acyls, 18 were carboxylic acids and derivatives (17 of subclass amino acids, peptides, and analogues) and 16 were glycerophospholipids. A complete overview of the compound classification is presented in Supplementary Figure S17. Figure 4 provides a visualization of the significantly different compounds annotated at level 3 or above. Additionally, for each significant compound, individual boxplots illustrating the distribution across strain groups are provided in Supplementary Data 2.

As shown in the left panel of Fig. 4, the combined use of multiple extraction schemes, analysis type (HILIC POS and RPLC NEG), and data annotation strategies (patRoon and MS-DIAL) enabled a broader coverage of significant annotated compounds. Notably, RPLC NEG analyses with MS-DIAL—particularly for samples prepared with scheme 1—captured the majority of lipid compounds. This aligns with the recognition of MS-DIAL for lipidomics analysis [42, 90]; while some compounds were exclusively annotated with patRoon such as pantothenic acid, adenosine phosphate, and adenosine (in HILIC POS), and itaconic acid (in RPLC NEG). The use of different tools affected the results of feature annotation, and some features relevant to biological variation may be missed or filtered out in one workflow but retained in the other. These differences underscore how the choice of software and annotation strategy can influence the recovery of biomarkers.

Lipid annotation was substantially more successful with MS-DIAL compared to patRoon. Manual inspection confirmed good spectral matches for lysophosphatidylethanolamine (LPE) annotated at level 2a (such as LPE O-18:0 and LPE 17:0), while fragments in the MS^2^ spectra of fatty acids annotated at level 3b were generally sparse and non-specific. A reason for the lower number of lipid annotations in patRoon is that the implemented database PubChemLite does not include many lipids, unlike the MoNA library integrated into MetFrag. In contrast, MS-DIAL includes the Lipid BLAST in silico library [91] and other lipid spectra that are not incorporated in the measured MS/MS spectra included within MetFrag, enhancing its capacity to annotate lipid classes. This discrepancy underscores the complementarity of the tools and the need to select annotation strategies based on chemical class. In support of the annotation accuracy, the elution order of fatty acids in RPLC followed expected trends based on carbon chain length and degree of unsaturation (Supplementary Table S3).

Notably, the differences in results between patRoon and MS-DIAL are reduced when focusing on the statistically significant compounds. This may be partly due to the filtering effect of the ANOVA statistical analysis, which also narrows the focus to features showing consistent group differences, thereby excluding many low-quality or inconsistent peaks. It is also possible that some discrepancies between the tools arise from differences in annotation thresholds, with MS-DIAL being too inclusive and patRoon too conservative, resulting in the different consideration of borderline or low-quality features.

Among the 87 unique statistically significant compounds annotated at level 3 or above considering all the different elaboration strategies implemented, 47 valid KEGG codes were available and thus used for enrichment analysis using the FELLA package. Results of the comprehensive enrichment analysis, considering all significant annotated compounds, are reported in Table S4 and in Figure S18. In addition, enrichment analyses focused on significant compounds from the pairwise comparisons between each strain and the wild type are detailed in Supplementary Tables S5–S8. The following sections discuss the biological implications of the significant compounds annotated for each knockout and transgenic strain, providing insights into potential metabolic alterations linked to xenobiotic metabolism and neurodegenerative processes. These interpretations are exploratory and should be considered as hypothesis-generating; further validation using targeted approaches and reference standards will be essential to confirm the observed metabolic alterations, but is beyond the scope of the current study.

#### Knockout strain in a gene encoding cytochrome P450 (“VC40”)

Comparing the wild-type sample group (N2) with the knockout strain “VC40”, 18 annotated compounds were higher in N2, while 10 were lower. The VC40 has a knockout in the *cyp-13A7* gene, which encodes cytochrome P450 enzymes (CYPs). These enzymes catalyze monooxygenase reactions for xenobiotic metabolism [92] and play a role in the biosynthesis and biodegradation of endogenous compounds, including fatty acids [9]. In line with this, several fatty acids were significantly lower in VC40 compared to wild type, including lysophosphatidylethanolamine (LPE) 17:0, 16:0, and 18:1; and fatty acids (FA) 18:1, 17:1, and 15:0. Previous studies have reported that the *cyp-13A7* gene is upregulated in *C. elegans* Dauer larvae [93], suggesting that gene silencing could disrupt Dauer formation. Notably, spermidine—a polyamine derived from putrescine [94]—was significantly higher in the wild-type group in our study. Putrescine itself is formed via decarboxylation of ornithine and arginine [95], further supporting the notion that the loss of c*yp-13A7* may influence key metabolic pathways related to development and cellular stress responses.

#### Knockout strain in a gene encoding for flavin-containing monooxygenases (“VC1668”)

In the comparison between wild type and the “VC1668” knockout strain, 69 annotated compounds were significantly higher in the wild type group, while only 2 were lower. The VC1668 strain carries a knockout in *fmo-2*, one of five genes in *C. elegans* encoding flavin-containing monooxygenases (FMO)—enzymes that catalyze the oxidation of nucleophilic heteroatom-containing compounds (e.g., nitrogen and sulphur substrates) [96]. FMOs are involved in the oxidation of both xenobiotics and endogenous compounds [97]. Recent research has demonstrated that *fmo-2* plays a role in regulating one-carbon metabolism [98], which encompasses the folate and methionine cycles. Intermediates from these cycles are a focal point for regulating longevity and/or age-related metabolic processes, including transsulfuration and lipid metabolism [99, 100]. Indeed, our analyses revealed significant differences in lipid profiles between the *fmo-2* knockout strain and the wild type, including LPE 16:0, LPE 17:0, LPE 18:0, LPE 18:1, LPE O-18:0, and some fatty acids. Choi and colleagues confirmed that *fmo-2* expression impacts endogenous metabolism: they performed non-targeted and targeted metabolomics analyses on *C. elegans* comparing wild type to both *fmo-2* overexpression and *fmo-2* knockout [98] and observed significant differences in polar metabolites, particularly between wild type and the overexpression strains, such as for homocysteine, s-adenosylmethionine, cystathionine, and pyridoxal 5′-phosphate. Considering the knockout strains (the same strain we analyzed), Choi et al. found significantly higher levels of phenylalanine in the wild type, results in agreement with our analyses. Conversely, we also found significant differences in tryptophan levels, for which Choi et al. did not find a significant difference. Furthermore, Choi et al. proposed that FMO-2 interacts with the target of rapamycin (mTOR) pathway, consistent with our pathway results. These results indicate a link between *fmo-2* activity, metabolic regulation, and broader pathways for cellular growth.

#### Transgenic strain related to Parkinson’s disease (“UA57”)

For the PD-related transgenic strain “UA57” (*baIs4 [dat-1p::GFP* + *dat-1p::CAT-2]*), 70 annotated compounds were significantly higher in the wild-type group, while 3 were lower. This strain overexpresses the gene *cat-2*, which encodes a protein that is equivalent to mammalian tyrosine hydroxylase, a rate-limiting enzyme in dopamine synthesis. This strain is characterized by dopamine overproduction in dopaminergic neurons and has been used to model key aspects of Parkinson’s disease, specifically dysregulated dopamine homeostasis [11, 12, 101]. Some metabolites identified as significantly altered in this strain have been previously suggested as potential PD biomarkers in human biological fluids (plasma, serum, urine, or cerebrospinal fluid) [102]. These include amino acids such as alanine [103–106], threonine [103, 106], tryptophan [105, 107], leucine/isoleucine [103, 106], and valine [103]. In addition, metabolites such as spermidine [108] and hypoxanthine [108, 109] were also significantly altered, supporting their potential role in PD-related metabolic dysregulation.

#### Transgenic strain related to Alzheimer’s disease (“BR5270”)

In the Alzheimer (AD)-related transgenic strain “BR5270,” 75 annotated compounds were significantly higher in the wild type, while 4 were lower. This strain overexpresses the pro-aggregation fragment of tau, leading to tau aggregation and tauopathy-related neurodegeneration. Tau is essential for microtubule stabilization under physiological conditions, but hyperphosphorylation of tau can lead to neurofibrillary tangles—one of the key pathological features of AD [13, 110]. In *C. elegans*, pan-neuronal tau expression induces insoluble, phosphorylated aggregates, causing neurodegeneration and locomotion defects that mirror human tauopathy [111]. Several metabolites that were significantly altered in BR5270 were previously suggested as potential AD biomarkers in human studies [102, 112]. Based on a proposed classification for AD pathophysiology, these include β-amyloid, phospho-tau, or neuronal injury [113]. Specifically, our analyses and previous studies have determined amino acids associated with these pathologies, spanning threonine [114], tryptophan [115–121], valine [114, 115, 122–125], and leucine/isoleucine [125]. Among the other altered metabolites in this strain, choline was found to be a potential disease progression biomarker [122]. Together, our findings support the relevance of the BR5270 model in recapitulating key metabolic changes associated with AD and highlight potential biomarkers for future investigation.

#### Comparative overview of metabolite differences across the strains

Considering the metabolite differences between the wild type and all the four mutant strains, some analogies and differences can be noted: particularly, several compounds were consistently lower in both the *fmo-2* knockout strain (VC1668) and the transgenic strain related to Parkinson’s (UA57) and Alzheimer’s disease (BR5270), including fatty acids such as LPEs; amino acids like isoleucine/leucine, phenylalanine, threonine, tryptophan, and alanine; and some other metabolites such as hypoxanthine, niacinamide, and inosine. Only a few metabolites were consistently lower in all of the four strains, such as LPE 17:0, 16:0, and 18:1 and some fatty acids and spermidine. In contrast, other metabolites were significantly higher only in the cytochrome P450 knockout strain (VC40), such as isoleucine, pantothenic acid, and phenylacetaldehyde. Interestingly, choline was the only metabolite that was consistently elevated not only in the cytochrome P450 knockout strain (VC40), but also in the neurodegenerative models (UA57, BR5270). Choline is a precursor for acetylcholine, a neurotransmitter whose impairment is involved in both Alzheimer’s and Parkinson’s disease [126, 127]. Overall, these observations highlight both converging and diverging metabolic signatures among the strains.

## Conclusions

In this work, we developed workflows for non-targeted metabolomics analyses of *C. elegans* samples, leveraging open science resources. We compared two metabolite extraction schemes: a monophasic approach, which provided broader metabolite coverage particularly in HILIC analyses, and a biphasic approach, which yielded a higher number of features in RPLC analyses. All data were processed using the open source tool patRoon, integrating algorithms such as IPO, XCMS, CAMERA, and MetFrag. Compound annotation with MetFrag utilized two chemical databases, PubChemLite and expanded WormJam databases, incorporating *C. elegans*–specific metabolites curated from PubChem and the literature. In addition, data were processed using the open software MS-DIAL, which enabled the annotation of a larger number of compounds, particularly when implementing the public MSP libraries. To enhance annotation reliability and coverage, we generated MSP libraries by in silico fragmentation of compounds from the expanded WormJam using CFM-ID. Although this approach yielded fewer candidates compared to public MSP libraries, it resulted in more reliable annotations, demonstrated by the absence of the unexpected annotations observed with public MSPs in MS-DIAL. Significant metabolite differences were observed when comparing knockout strains of xenobiotic-metabolizing enzymes and transgenic strains related to neurodegenerative diseases, with results generally aligning with existing literature. The use of strain-specific pooled quality control samples ensured high data quality and facilitated the accurate detection of strain-related metabolites.

While the parallel use of multiple data processing tools expanded the annotation landscape, our findings underscore the importance of critically evaluating and understanding the discrepancies among software outputs—an area warranting thorough investigation and harmonization. In non-targeted metabolomics, the goal should not necessarily be to maximize the number of tentative annotations, but rather to derive a manageable and interpretable set of reliable candidate compounds that can be prioritized for further validation using authentic chemical standards. Overall, the non-targeted approach described in this work may be valuable for metabolite discovery and/or hypothesis generation, paving ways for follow-up sensitive, targeted analyses that are equally essential for validation and in-depth etiologic investigation.

## Supplementary Information

Below is the link to the electronic supplementary material.

Supplementary file1 (PDF 1898 KB)

Supplementary file2 (PDF 6465 KB)

Supplementary file3 (XLSX 266 KB)

## Data Availability

The expanded WormJam chemical list and related MSP libraries, the raw files of LC–MS/MS analyses, and all the tables of features and following elaborations are reported in a Zenodo repository [51]. All the code written for data elaboration are available in a GitHub repository [65]. The code to produce the WormJam extended database is available on GitLab [57].
