# Supplementary material for: Leveraging open cheminformatics tools for non-targeted metabolomics analysis of C. elegans: a workflow comparison and application to strains related to xenobiotic metabolism and neurodegeneration
Source: Anal Bioanal Chem. 2025 Aug 8;417(27):6089–106. doi: 10.1007/s00216-025-06048-y (PMC12583387; doi:10.1007/s00216-025-06048-y)
Supplement: Supplementary file 1 — Supplementary file1 (PDF 1898 KB) [file 216_2025_6048_MOESM1_ESM.pdf]

Leveraging open cheminformatics tools for non-targeted metabolomics analysis of *C. elegans*: a workflow comparison and application to strains related to xenobiotic metabolism and neurodegeneration

Gianfranco Frigerio[1,2]*, Yunjia Lai[3], Emma L. Schymanski[1]*, Gary W. Miller[3].

[1] Luxembourg Centre for Systems Biomedicine (LCSB), University of Luxembourg, 6, Avenue du Swing, L-4367 Belvaux, Luxembourg

[2] Center for Omics Sciences (COSR), IRCCS San Raffaele Scientific Institute, Milan, Italy

[3] Department of Environmental Health Sciences, Mailman School of Public Health at Columbia University, New York, NY, USA

* Corresponding authors: GF: frigerio.gianfranco@hsr.it & ELS: emma.schymanski@uni.lu

ORCIDs: GF: 0000-0002-3538-1443, YL: 0000-0002-1081-0897, ELS: 0000-0001-6868-8145, GWM: 0000-0001-8984-1284

Supplementary data 1

Supplementary figures S1: Summary of the number of features obtained from the elaboration of the different combinations of analyses performed in RPLC NEG and HILIC POS, using patRoon or MS-DIAL. The total number of features initially retrieved was reduced to include only those passing the pooled QC check, and further divided by sample preparation scheme. The lower section shows the number of statistically significant features (ANOVA) among the groups of strains.

Supplementary figures S2, S5, S8, S11: For each of the four combinations of analysis type and data elaboration tool used, two Eulero-venn diagrams are reported: the first one shows the features that passed the QC check that are in common among the considered extraction schemes (scheme 1 or scheme 2); the second one, analogously, shows features statistically significant among sample groups.

Supplementary figures S3-S4, S5-S7, S9-S10, S12-S13: For each of the four combinations of analysis type and data elaboration tool used, a table reporting the number of annotated features, according to the levels of reporting, is shown for the total number of features; a similar table is then reported for features that passed the QC criteria; in the second column a similar table is reported separately considering samples treated with scheme 1 and scheme 2; in the third column similar tables are reported for features that were significantly different among considered groups.

Supplementary figure S14: Upset plot showing the unique compounds annotated at level 3 or above, obtained considering all samples preparation schemes and chromatographic runs, grouped by the four annotation strategies implemented and showing the intersection among them.

Supplementary figure S15: SankeyNetwork graph illustrating an overview of the compound classes of annotated compounds at level 3 or above.

Supplementary figure S16: SankeyNetwork graph illustrating an overview of the compound classes of annotated compounds at level 3 or above with patRoon.

Supplementary figure S17: SankeyNetwork graph illustrating an overview of the compound classes of annotated compounds at level 3 or above that were significantly different among the considered groups of strains.

Supplementary figure S18: Visual representation of the enrichment analyses performed with the FELLA package considering all the annotated compounds that were significantly different among the considered groups of strains.

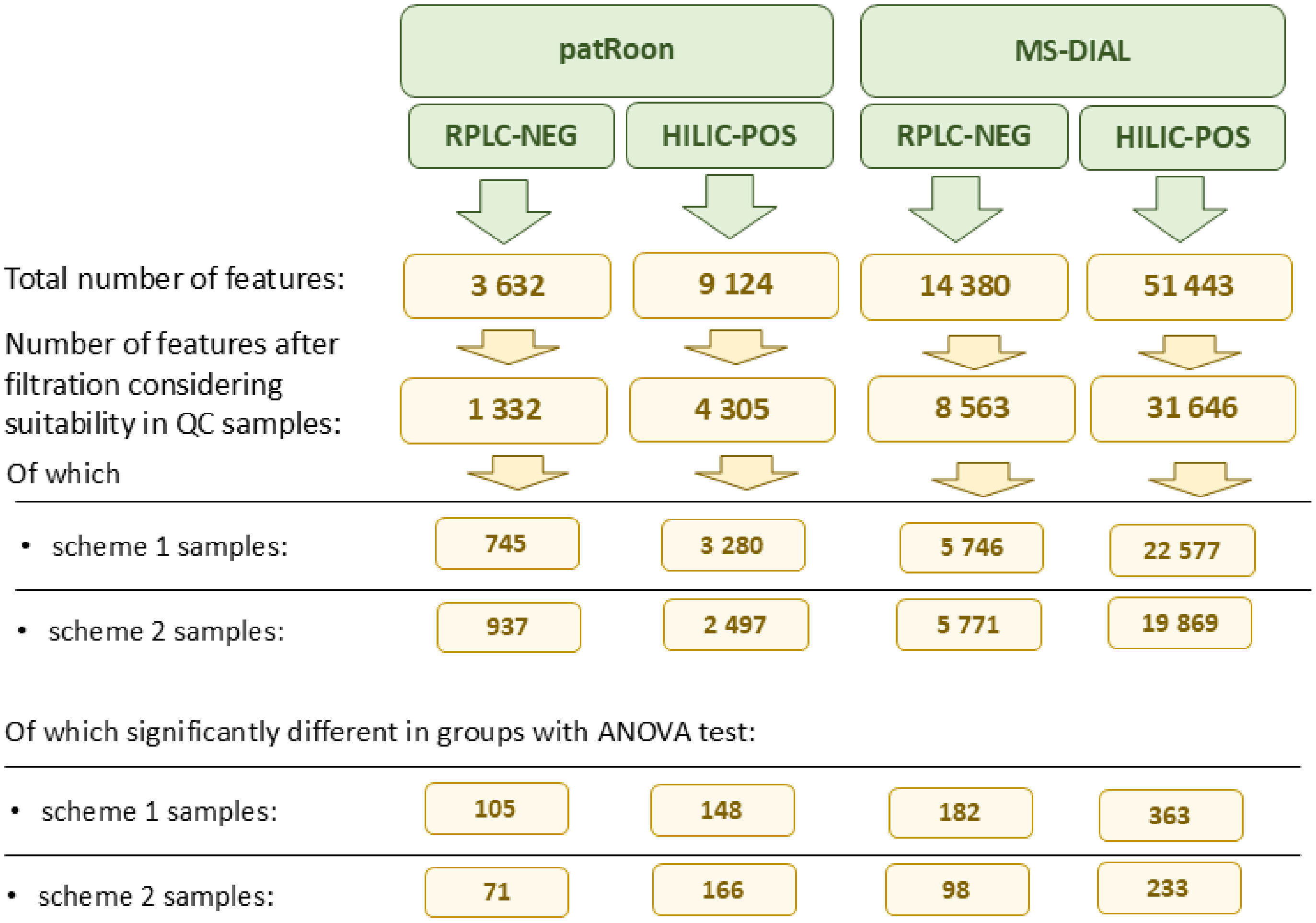

Supplementary figures S1: Summary of the number of features obtained from the elaboration of the different combinations of analyses performed in RPLC NEG and HILIC POS, using patRoon or MS-DIAL. The total number of features initially retrieved was reduced to include only those passing the pooled QC check, and further divided by sample preparation scheme. The lower section shows the number of statistically significant features (ANOVA) among the groups of strains.

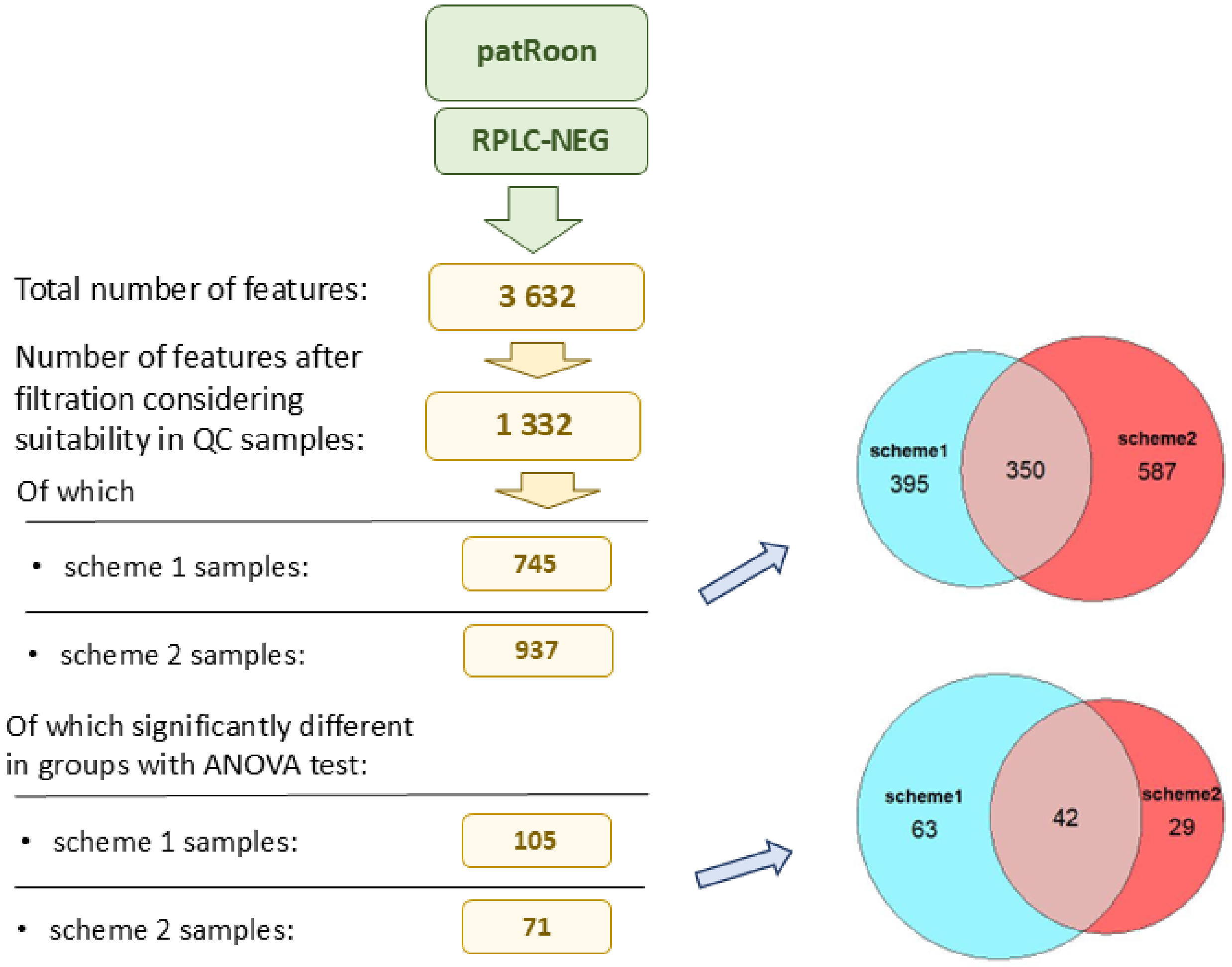

Supplementary figures S2: For RPLC NEG analyses elaborated with patRoon, two Eulero-venn diagrams are reported: the first one shows the features that passed the QC check that are in common among the considered extraction schemes (scheme 1 or scheme 2); the second one, analogously, shows features statistically significant among sample groups.

patRoon **RPLC-NEG** **PubChemLite**

Total features:

Annotation Level	N	%
2a	14	0.4
3a	1	0
3b	9	0.2
4a	371	10.2
5	3237	89.1
TOTAL:	3632	99.9

Features after filtration considering suitability in QC samples:

Annotation Level	N	%
2a	6	0.5
3a	1	0.1
3b	5	0.4
4a	189	14.2
5	1131	84.9
TOTAL:	1332	100.1

Features after filtration considering suitability in QC samples, only in scheme 1 samples:

Annotation Level	N	%
2a	6	0.8
3a	1	0.1
3b	3	0.4
4a	135	18.1
5	600	80.5
TOTAL:	745	99.9

Features after filtration considering suitability in QC samples, only in scheme 2 samples:

Annotation Level	N	%
2a	3	0.3
3b	3	0.3
4a	121	12.9
5	810	86.4
TOTAL:	937	99.9

Features after filtration considering suitability in QC samples, and significantly different in groups with ANOVA test, only in scheme 1 samples:

Annotation Level	N	%
2a	1	1
3b	1	1
4a	24	22.9
5	79	75.2
TOTAL:	105	100.1

Features after filtration considering suitability in QC samples, and significantly different in groups with ANOVA test, only in scheme 2 samples:

Annotation Level	N	%
3b	1	1.4
4a	18	25.4
5	52	73.2
TOTAL:	71	100

Supplementary figures S3: For RPLC NEG analyses elaborated with patRoon with the PubChemLite database, a table reporting the number of annotated features, according to the levels of reporting, is shown for the total number of features; a similar table is then reported for features that passed the QC criteria; in the second column a similar table is reported separately considering samples treated with scheme 1 and scheme 2; in the third column similar tables are reported for features that were significantly different among considered groups.

patRoon | **RPLC-NEG** | **WormJam expanded**

Total features:

Annotation Level	N	%
2a	16	0.4
3b	10	0.3
4a	142	3.9
5	3464	95.4
TOTAL:	3632	100

Features after filtration considering suitability in QC samples:

Annotation Level	N	%
2a	7	0.5
3b	5	0.4
4a	88	6.6
5	1232	92.5
TOTAL:	1332	100

Features after filtration considering suitability in QC samples, only in scheme 1 samples:

Annotation Level	N	%
2a	6	0.8
3b	3	0.4
4a	74	9.9
5	662	88.9
TOTAL:	745	100

Features after filtration considering suitability in QC samples, only in scheme 2 samples:

Annotation Level	N	%	
2a		3	0.3
3b		3	0.3
4a		49	5.2
	5	882	94.1
TOTAL:		937	99.9

Features after filtration considering suitability in QC samples, and significantly different in groups with ANOVA test, only in scheme 1 samples:

Annotation Level	N	%	
2a		2	1.9
3b		1	1
4a		21	20
	5	81	77.1
TOTAL:		105	100

Features after filtration considering suitability in QC samples, and significantly different in groups with ANOVA test, only in scheme 2 samples:

Annotation Level	N	%	
3b		1	1.4
4a		13	18.3
	5	57	80.3
TOTAL:		71	100

Supplementary figures S4: For RPLC NEG analyses elaborated with patRoon with the WormJam expanded database, a table reporting the number of annotated features, according to the levels of reporting, is shown for the total number of features; a similar table is then reported for features that passed the QC criteria; in the second column a similar table is reported separately considering samples treated with scheme 1 and scheme 2; in the third column similar tables are reported for features that were significantly different among considered groups.

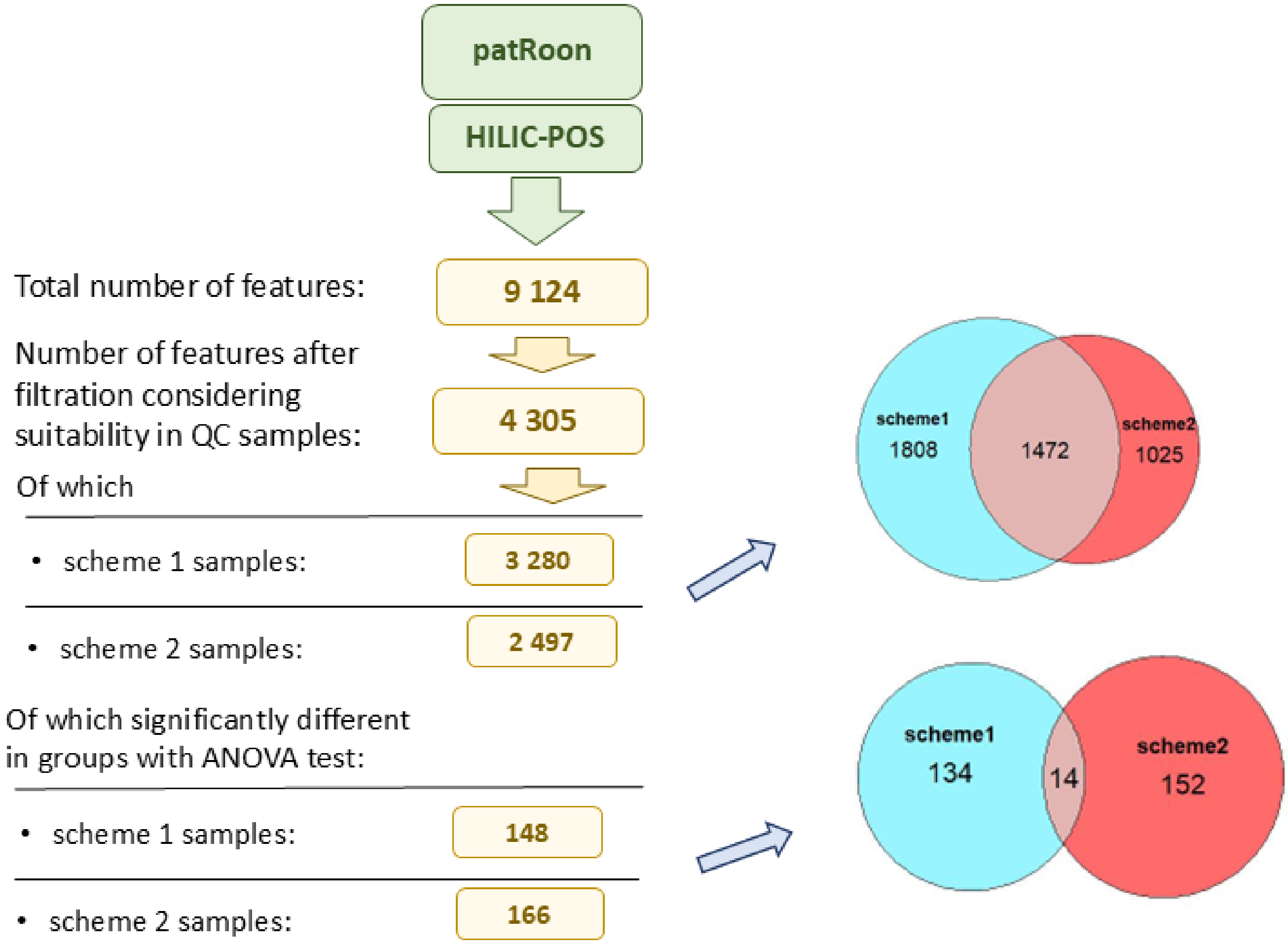

Supplementary figures S5: For HILIC POS analyses elaborated with patRoon, two Eulero-venn diagrams are reported: the first one shows the features that passed the QC check that are in common among the considered extraction schemes (scheme 1 or scheme 2); the second one, analogously, shows features statistically significant among sample groups.

patRoon | HILIC-POS | PubChemLite

Total features:

Annotation Level	N	%
2a	15	0.2
3a	8	0.1
3b	6	0.1
4a	1372	15
5	7723	84.6
TOTAL:	9124	100

Features after filtration considering suitability in QC samples:

Annotation Level	N	%
2a	12	0.3
3a	8	0.2
3b	5	0.1
4a	872	20.3
5	3408	79.2
TOTAL:	4305	100.1

Features after filtration considering suitability in QC samples, only in scheme 1 samples:

Annotation Level	N	%
2a	12	0.4
3a	8	0.2
3b	5	0.2
4a	672	20.5
5	2583	78.8
TOTAL:	3280	100.1

Features after filtration considering suitability in QC samples, only in scheme 2 samples:

Annotation Level	N	%
2a	11	0.4
3a	6	0.2
3b	4	0.2
4a	592	23.7
5	1884	75.5
TOTAL:	2497	100

Features after filtration considering suitability in QC samples, and significantly different in groups with ANOVA test, only in scheme 1 samples:

Annotation Level	N	perc
2a	1	0.7
4a	29	19.6
5	118	79.7
TOTAL:	148	100

Features after filtration considering suitability in QC samples, and significantly different in groups with ANOVA test, only in scheme 2 samples:

Annotation Level	N	perc
2a	4	2.4
3a	1	0.6
4a	41	24.7
5	120	72.3
TOTAL:	166	100

Supplementary figures S6: For HILIC POS analyses elaborated with patRoon with the PubChemLite database, a table reporting the number of annotated features, according to the levels of reporting, is shown for the total number of features; a similar table is then reported for features that passed the QC criteria; in the second column a similar table is reported separately considering samples treated with scheme 1 and scheme 2; in the third column similar tables are reported for features that were significantly different among considered groups.

patRoon | HILIC-POS | WormJam expanded

Total features:

Annotation Level	N	perc
2a	11	0.1
3a	7	0.1
3b	4	0
4a	229	2.5
5	8873	97.2
TOTAL:	9124	99.9

Features after filtration considering suitability in QC samples:

Annotation Level	N	%
2a	9	0.2
3a	7	0.2
3b	3	0.1
4a	168	3.9
5	4118	95.7
TOTAL:	4305	100.1

Features after filtration considering suitability in QC samples, only in scheme 1 samples:

Annotation Level	N	%
2a	9	0.3
3a	7	0.2
3b	3	0.1
4a	138	4.2
5	3123	95.2
TOTAL:	3280	100

Features after filtration considering suitability in QC samples, only in scheme 2 samples:

Annotation Level	N	%
2a	9	0.4
3a	5	0.2
3b	3	0.1
4a	127	5.1
5	2353	94.2
TOTAL:	2497	100

Features after filtration considering suitability in QC samples, and significantly different in groups with ANOVA test, only in scheme 1 samples:

Annotation Level	N	%
2a	1	0.7
4a	12	8.1
5	135	91.2
TOTAL:	148	100

Features after filtration considering suitability in QC samples, and significantly different in groups with ANOVA test, only in scheme 2 samples:

Annotation Level	N	%
2a	4	2.4
3a	1	0.6
4a	19	11.4
5	142	85.5
TOTAL:	166	99.9

Supplementary figures S7: For HILIC POS analyses elaborated with patRoon with the WormJam expanded database, a table reporting the number of annotated features, according to the levels of reporting, is shown for the total number of features; a similar table is then reported for features that passed the QC criteria; in the second column a similar table is reported separately considering samples treated with scheme 1 and scheme 2; in the third column similar tables are reported for features that were significantly different among considered groups.

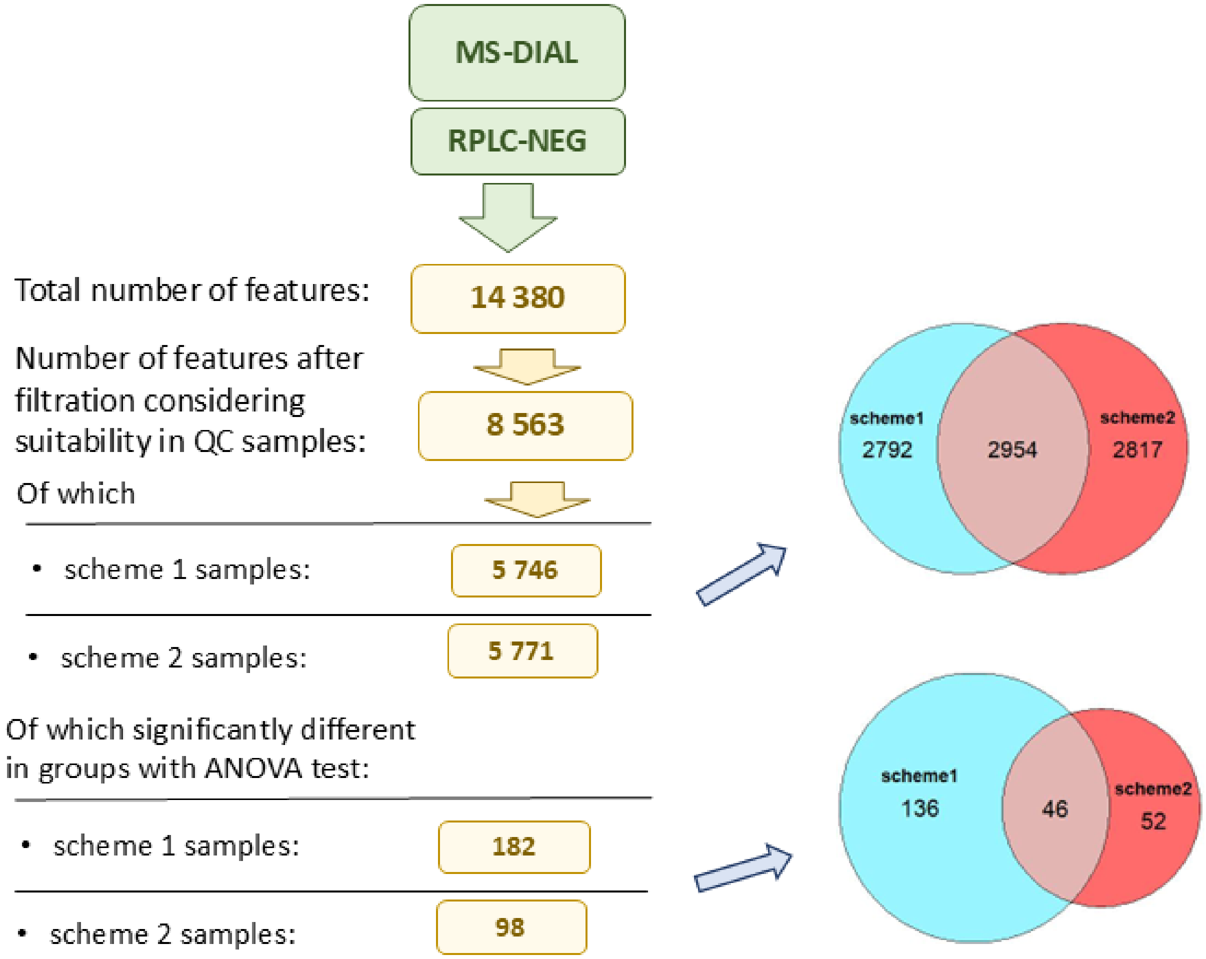

Supplementary figures S8: For RPLC NEG analyses elaborated with MS-DIAL, two Eulero-venn diagrams are reported: the first one shows the features that passed the QC check that are in common among the considered extraction schemes (scheme 1 or scheme 2); the second one, analogously, shows features statistically significant among sample groups.

MS-DIAL **RPLC-NEG** **Public MSP**

Total features:

Annotation Level	N	%
2a	50	0.3
2b	20	0.1
3a	59	0.4
3b	191	1.3
3c	19	0.1
4a	6063	42.2
5	7978	55.5
TOTAL:	14380	99.9

Features after filtration considering suitability in QC samples:

AnnotationLevel	N	%
2a	37	0.4
2b	19	0.2
3a	42	0.5
3b	134	1.6
3c	11	0.1
4a	4095	47.8
5	4225	49.3
TOTAL:	8563	99.9

Features after filtration considering suitability in QC samples, only in scheme 1 samples:

AnnotationLevel	N	%
2a	33	0.6
2b	15	0.3
3a	31	0.5
3b	111	1.9
3c	7	0.1
4a	2839	49.4
5	2710	47.2
TOTAL:	5746	100

Features after filtration considering suitability in QC samples, only in scheme 2 samples:

Annotation Level	N	%
2a	14	0.2
2b	16	0.3
3a	23	0.4
3b	97	1.7
3c	7	0.1
4a	2746	47.6
5	2868	49.7
TOTAL:	5771	100

Features after filtration considering suitability in QC samples, and significantly different in groups with ANOVA test, only in scheme 1 samples:

Annotation Level	N	%
2a	8	4.4
3a	3	1.6
3b	27	14.8
3c	1	0.5
4a	105	57.7
5	38	20.9
TOTAL:	182	99.9

Features after filtration considering suitability in QC samples, and significantly different in groups with ANOVA test, only in scheme 2 samples:

Annotation Level	N	%
3b	16	16.3
3c	1	1
4a	41	41.8
5	40	40.8
TOTAL:	98	99.9

Supplementary figures S9 For RPLC NEG analyses elaborated with MS-DIAL with the public MPS libraries, a table reporting the number of annotated features, according to the levels of reporting, is shown for the total number of features; a similar table is then reported for features that passed the QC criteria; in the second column a similar table is reported separately considering samples treated with scheme 1 and scheme 2; in the third column similar tables are reported for features that were significantly different among considered groups.

MS-DIAL | RPLC-NEG | WormJam expanded

Total features:

Annotation Level	N	%
2a	9	0.1
3a	30	0.2
3b	66	0.5
4a	2431	17.1
5	11679	82.2
TOTAL:	14215	100.1

Features after filtration considering suitability in QC samples:

Annotation Level	N	%
2a	8	0.1
3a	21	0.2
3b	44	0.5
4a	1768	20.9
5	6623	78.2
TOTAL:	8464	99.9

Features after filtration considering suitability in QC samples, only in scheme 1 samples:

Annotation Level	N	%
2a	7	0.1
3a	17	0.3
3b	31	0.5
4a	1307	23
5	4317	76
TOTAL:	5679	99.9

Features after filtration considering suitability in QC samples, only in scheme 2 samples:

Annotation Level	N	%
2a	3	0.1
3a	12	0.2
3b	22	0.4
4a	1147	20.1
5	4520	79.2
TOTAL:	5704	100

Features after filtration considering suitability in QC samples, and significantly different in groups with ANOVA test, only in scheme 1 samples:

Annotation Level	N	%
2a	1	0.6
3b	2	1.1
4a	41	22.8
5	136	75.6
TOTAL:	180	100.1

Features after filtration considering suitability in QC samples, and significantly different in groups with ANOVA test, only in scheme 2 samples:

Annotation Level	N	%
2a	1	1.1
4a	27	28.7
5	66	70.2
TOTAL:	94	100

Supplementary figures S10: For RPLC NEG analyses elaborated with MS-DIAL with the WormJam expanded MSP libraries, a table reporting the number of annotated features, according to the levels of reporting, is shown for the total number of features; a similar table is then reported for features that passed the QC criteria; in the second column a similar table is reported separately considering samples treated with scheme 1 and scheme 2; in the third column similar tables are reported for features that were significantly different among considered groups.

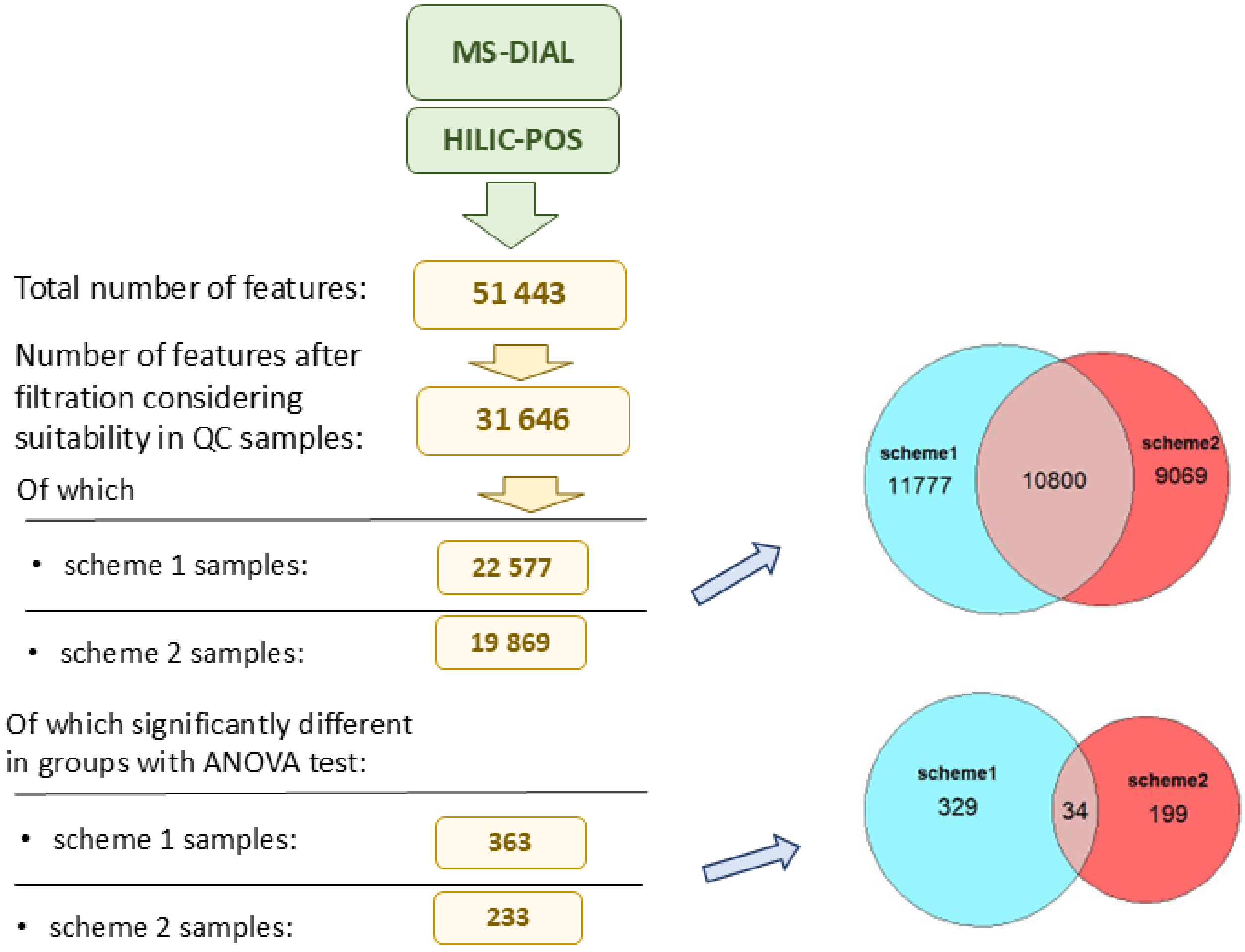

Supplementary figures S11: For HILIC POS analyses elaborated with MS-DIAL, two Eulero-venn diagrams are reported: the first one shows the features that passed the QC check that are in common among the considered extraction schemes (scheme 1 or scheme 2); the second one, analogously, shows features statistically significant among sample groups.

MS-DIAL **HILIC-POS** **Public MSP**

Total features:

Annotation Level	N	%
2a	1008	2
3a	517	1
3b	1830	3.6
4a	27355	53.2
5	20733	40.3
TOTAL:	51443	100.1

Features after filtration considering suitability in QC samples:

Annotation Level	N	%
2a	738	2.3
3a	369	1.2
3b	1256	4
4a	18091	57.2
5	11192	35.4
TOTAL:	31646	100.1

Features after filtration considering suitability in QC samples, only in scheme 1 samples:

Annotation Level	N	%
2a	595	2.6
3a	278	1.2
3b	943	4.2
4a	13049	57.8
5	7712	34.2
TOTAL:	22577	100

Features after filtration considering suitability in QC samples, only in scheme 2 samples:

Annotation Level	N	%
2a	468	2.4
3a	228	1.1
3b	773	3.9
4a	11449	57.6
5	6951	35
TOTAL:	19869	100

Features after filtration considering suitability in QC samples, and significantly different in groups with ANOVA test, only in scheme 1 samples:

Annotation Level	N	%
2a	13	3.6
3a	6	1.7
3b	15	4.1
4a	197	54.3
5	132	36.4
TOTAL:	363	100.1

Features after filtration considering suitability in QC samples, and significantly different in groups with ANOVA test, only in scheme 2 samples:

Annotation Level	N	%
2a	10	4.3
3a	5	2.1
3b	13	5.6
4a	94	40.3
5	111	47.6
TOTAL:	233	99.9

Supplementary figures S12: For HILIC POS analyses elaborated with MS-DIAL with the public MSP libraries, a table reporting the number of annotated features, according to the levels of reporting, is shown for the total number of features; a similar table is then reported for features that passed the QC criteria; in the second column a similar table is reported separately considering samples treated with scheme 1 and scheme 2; in the third column similar tables are reported for features that were significantly different among considered groups.

MS-DIAL | HILIC-POS | WormJam expanded

Total features:

Annotation Level	N	%
2a	120	0.2
3a	97	0.2
3b	162	0.3
4a	10648	21.3
5	39020	78
TOTAL:	50047	100

Features after filtration considering suitability in QC samples:

Annotation Level	N	%
2a	86	0.3
3a	72	0.2
3b	101	0.3
4a	7349	24
5	23028	75.2
TOTAL:	30636	100

Features after filtration considering suitability in QC samples, only in scheme 1 samples:

Annotation Level	N	%
2a	62	0.3
3a	60	0.3
3b	81	0.4
4a	5274	24.2
5	16352	74.9
TOTAL:	21829	100.1

Features after filtration considering suitability in QC samples, only in scheme 2 samples:

Annotation Level	N	%
2a	55	0.3
3a	47	0.2
3b	69	0.4
4a	4809	25.1
5	14217	74.1
TOTAL:	19197	100.1

Features after filtration considering suitability in QC samples, and significantly different in groups with ANOVA test, only in scheme 1 samples:

Annotation Level	N	%
3a	4	1.1
3b	4	1.1
4a	106	30.4
5	235	67.3
TOTAL:	349	99.9

Features after filtration considering suitability in QC samples, and significantly different in groups with ANOVA test, only in scheme 2 samples:

Annotation Level	N	%
2a	1	0.4
3a	2	0.9
4a	38	17
5	182	81.6
TOTAL:	223	99.9

Supplementary figures S13: For HILIC POS analyses elaborated with MS-DIAL with the WormJam expanded MSP libraries, a table reporting the number of annotated features, according to the levels of reporting, is shown for the total number of features; a similar table is then reported for features that passed the QC criteria; in the second column a similar table is reported separately considering samples treated with scheme 1 and scheme 2; in the third column similar tables are reported for features that were significantly different among considered groups.

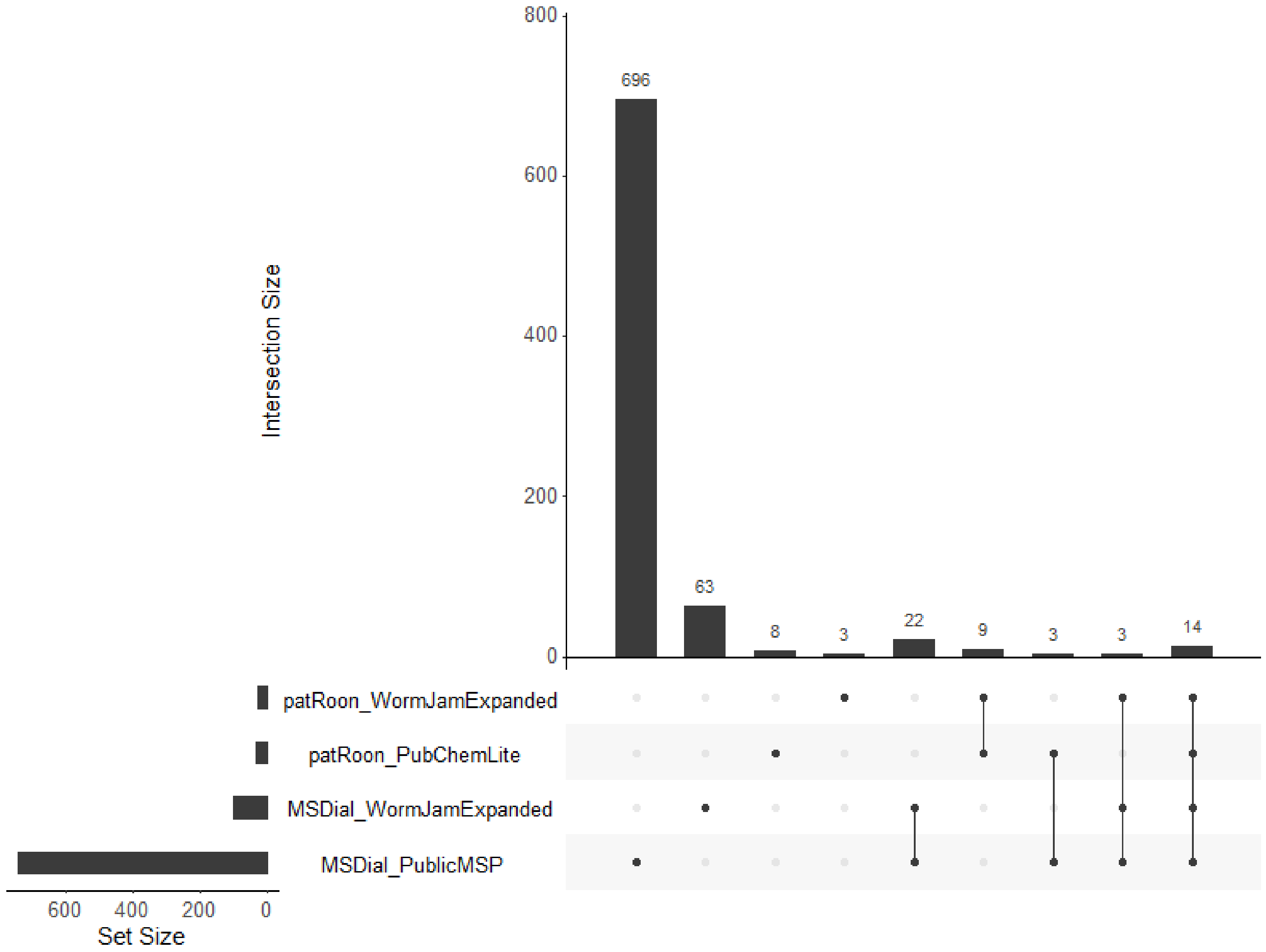

Supplementary figure S14: Upset plot showing the unique compounds annotated at level 3 or above, obtained considering all samples preparation schemes and chromatographic runs, grouped by the four annotation strategies implemented and showing the intersection among them.

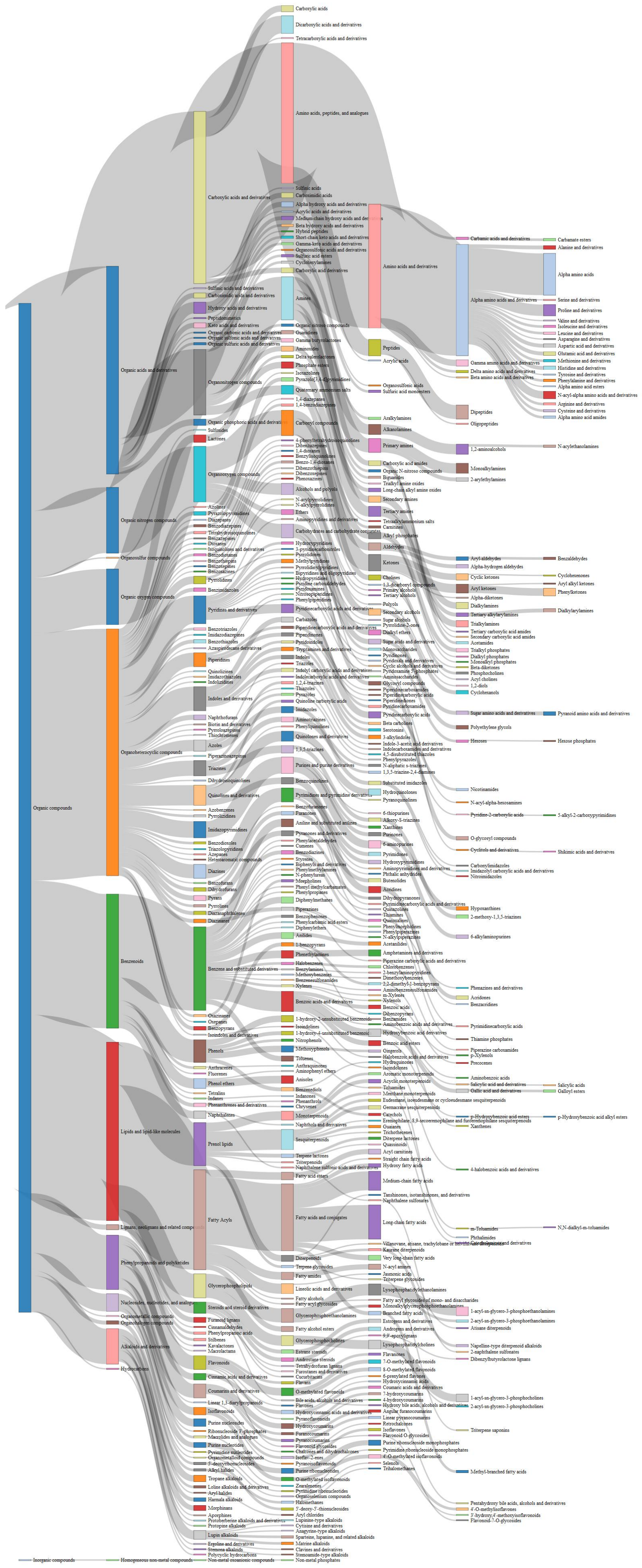

Supplementary figure S15: SankeyNetwork graph illustrating an overview of the compound classes of annotated compounds at level 3 or above.

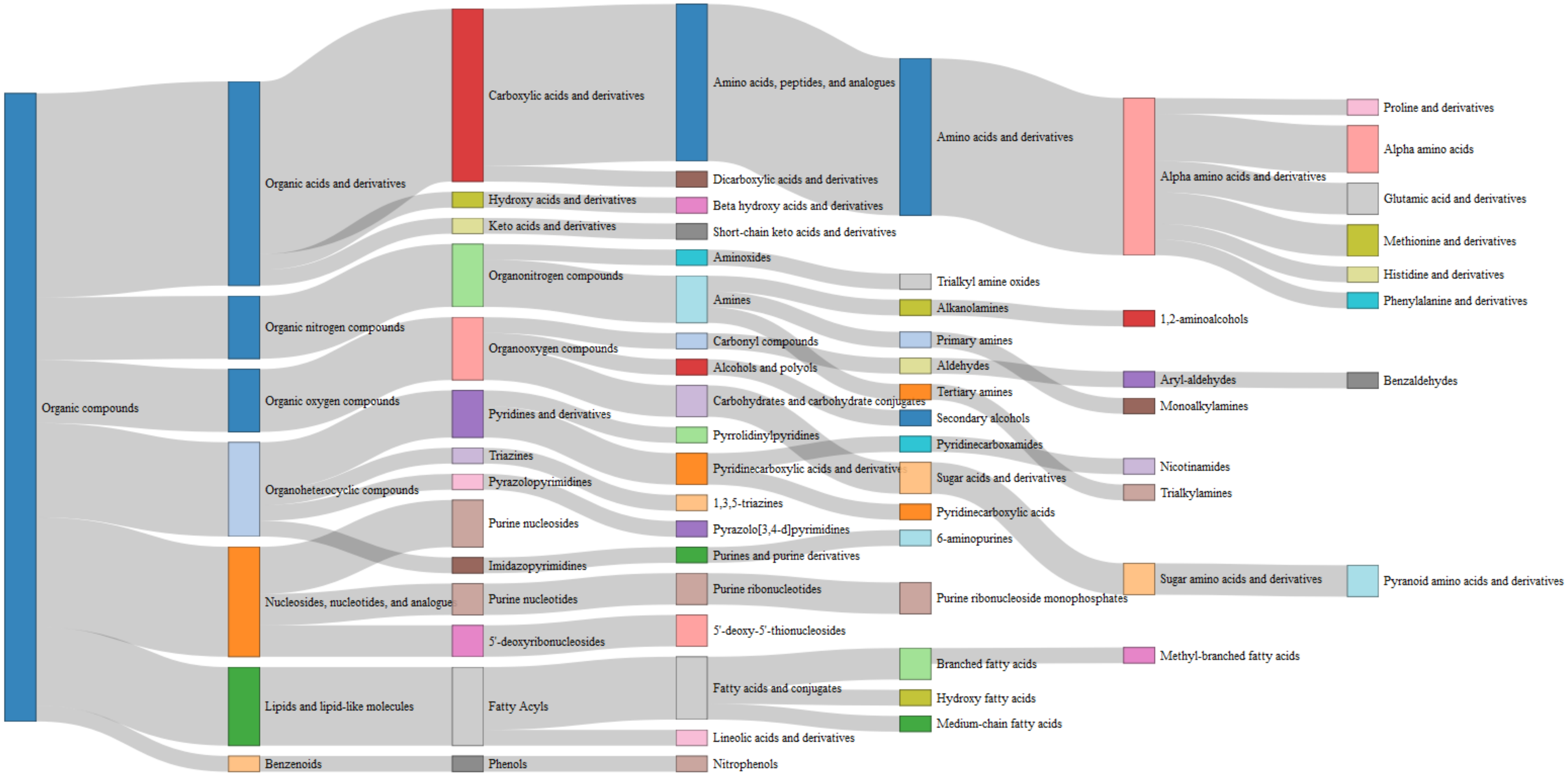

Supplementary figure S16: SankeyNetwork graph illustrating an overview of the compound classes of annotated compounds at level 3 or above with patRoon.

Supplementary data 1

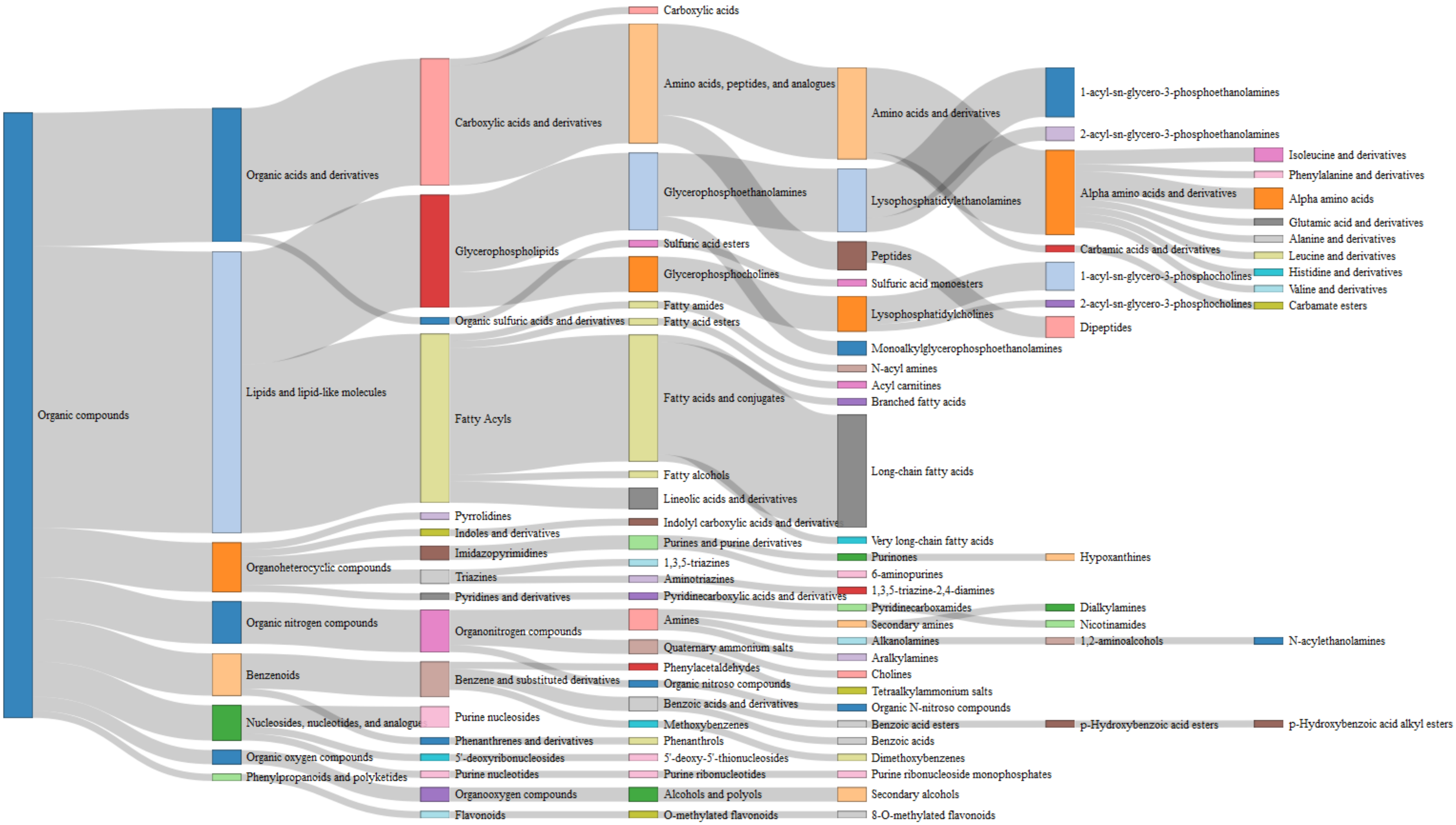

Supplementary figure S17: SankeyNetwork graph illustrating an overview of the compound classes of annotated compounds at level 3 or above that were significantly different among the considered groups of strains.

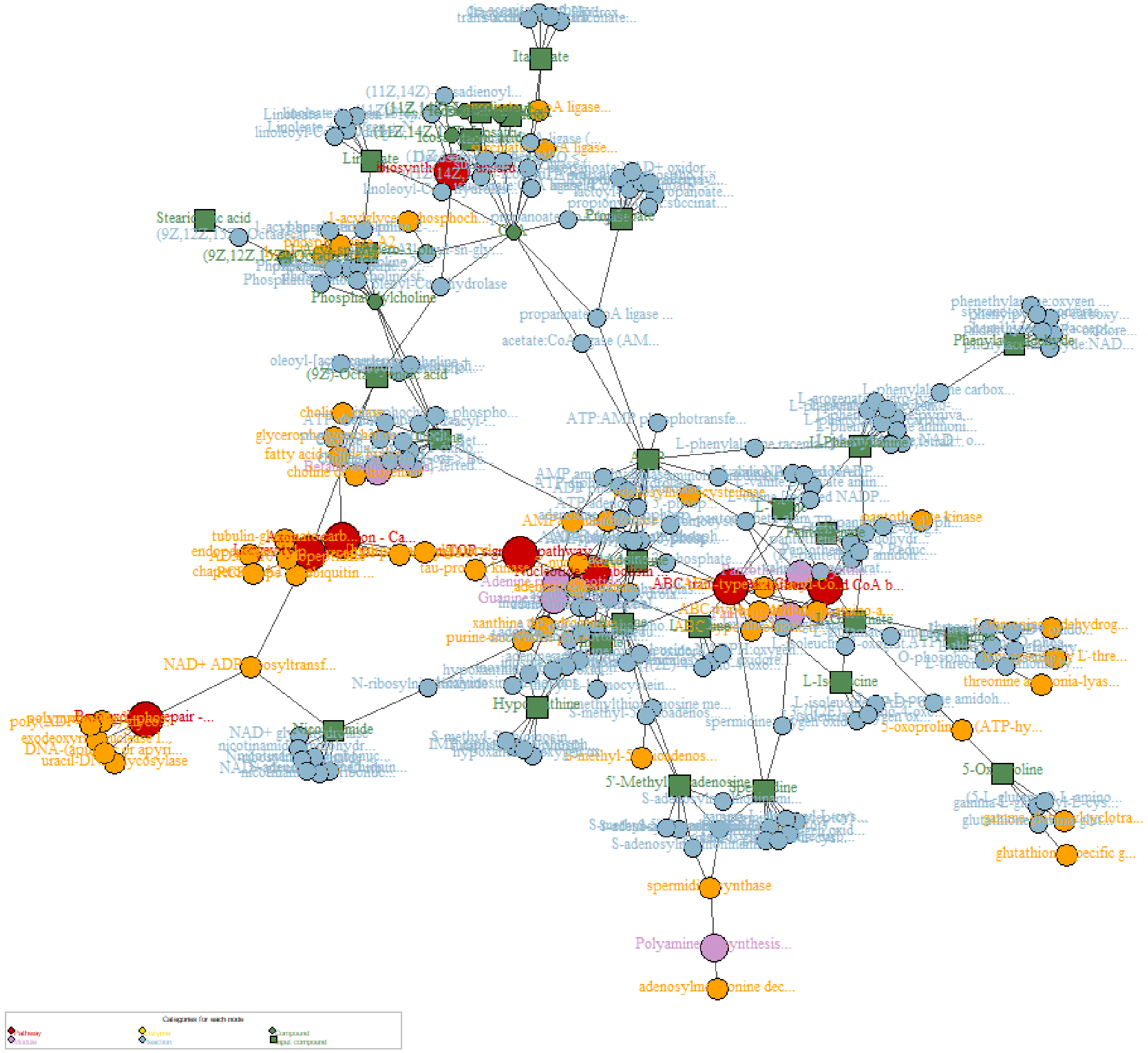

Supplementary figure S18: Visual representation of the enrichment analyses performed with the FELLA package considering all the annotated compounds that were significantly different among the considered groups of strains.
