# Supplementary material for: Leveraging open cheminformatics tools for non-targeted metabolomics analysis of C. elegans: a workflow comparison and application to strains related to xenobiotic metabolism and neurodegeneration
Source: Anal Bioanal Chem. 2025 Aug 8;417(27):6089–106. doi: 10.1007/s00216-025-06048-y (PMC12583387; doi:10.1007/s00216-025-06048-y)
Supplement: Supplementary file 2 — Supplementary file2 (PDF 6465 KB) [file 216_2025_6048_MOESM2_ESM.pdf]

Leveraging open cheminformatics tools for non-targeted metabolomics analysis of *C. elegans*: a workflow comparison and application to strains related to xenobiotic metabolism and neurodegeneration

Gianfranco Frigerio[1,2]*, Yunjia Lai[3], Emma L. Schymanski[1]*, Gary W. Miller[3].

[1] Luxembourg Centre for Systems Biomedicine (LCSB), University of Luxembourg, 6, Avenue du Swing, L-4367 Belvaux, Luxembourg

[2] Center for Omics Sciences (COSR), IRCCS San Raffaele Scientific Institute, Milan, Italy

[3] Department of Environmental Health Sciences, Mailman School of Public Health at Columbia University, New York, NY, USA

* Corresponding authors: GF: frigerio.gianfranco@hsr.it & ELS: emma.schymanski@uni.lu

ORCIDs: GF: 0000-0002-3538-1443, YL: 0000-0002-1081-0897, ELS: 0000-0001-6868-8145, GWM: 0000-0001-8984-1284

Supplementary data 2

For each significant compound annotated at level 3 or above, a boxplot shows the distribution in the considered groups of strains. On the right, molecular information is reported, along with the FDR corrected p-value of the ANOVA analysis, the significant pairwise comparison according to the Fisher's LSD test, and the type of analysis in which the molecule was found.

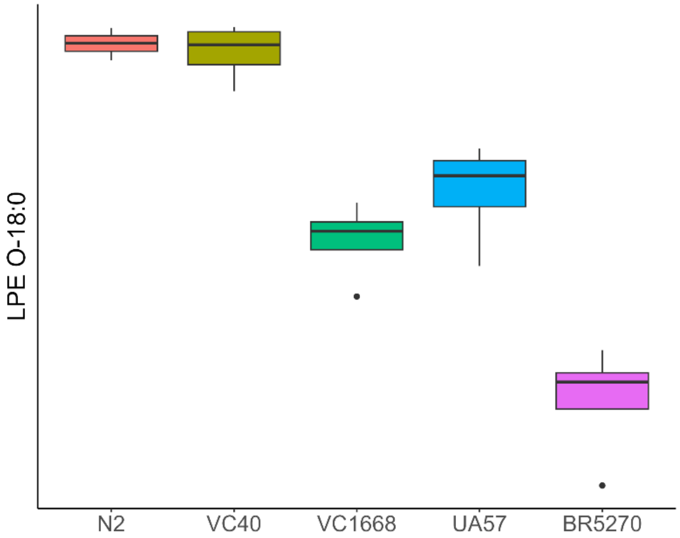

Pubchem CID: 74819129
InChIKey: JIJGULMDDZORPH-UHFFFAOYSA-N
Title: 2-Aminoethyl (2-hydroxy-3-octadecoxypro
Compound name: LPE O-18:0
Name used: LPE O-18:0
Annotation level: 2a
ANOVA FDR PValue: 1.196e-07
Fisher's LSD pairwise comparison:
N2 > VC1668 - Pvalue: 6.409e-07
N2 > UA57 - Pvalue: 2.750e-05
N2 > BR5270 - Pvalue: 5.023e-11
VC40 > VC1668 - Pvalue: 1.314e-07
VC40 > UA57 - Pvalue: 8.316e-06
VC40 > BR5270 - Pvalue: 5.749e-12
VC1668 > BR5270 - Pvalue: 1.074e-05
UA57 > BR5270 - Pvalue: 2.776e-07
Type of analysis: RPLCNEG scheme1 MSDial PublicMSP

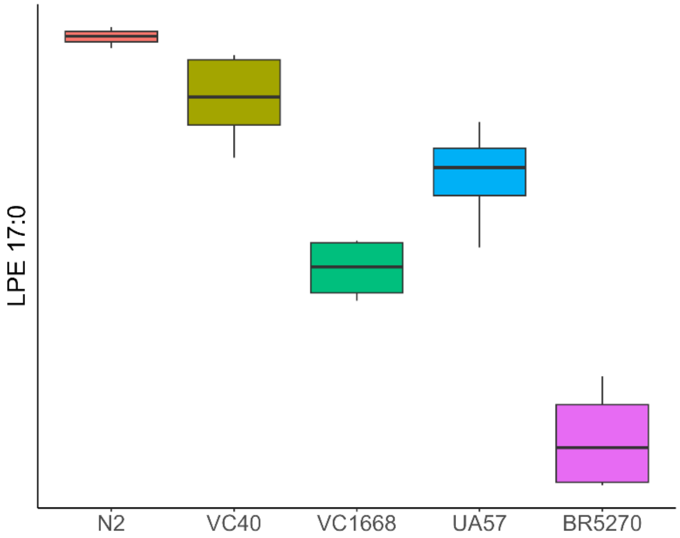

Pubchem CID: 75958076
InChIKey: RVNBVQKDPQVSOY-UHFFFAOYSA-N
Title: [3-[2-Aminoethoxy(hydroxy)phosphoryl]ox
Compound name: LPE 17:0
Name used: LPE 17:0
Annotation level: 2a
ANOVA FDR PValue: 1.270e-07
Fisher's LSD pairwise comparison:
N2 > VC40 - Pvalue: 0.0224
N2 > VC1668 - Pvalue: 1.264e-07
N2 > UA57 - Pvalue: 1.007e-04
N2 > BR5270 - Pvalue: 1.542e-11
VC40 > VC1668 - Pvalue: 1.325e-06
VC40 > UA57 - Pvalue: 0.0051
VC40 > BR5270 - Pvalue: 2.234e-11
UA57 > VC1668 - Pvalue: 0.0041
VC1668 > BR5270 - Pvalue: 9.168e-06
UA57 > BR5270 - Pvalue: 1.825e-08
Type of analysis: RPLCNEG scheme1 MSDial PublicMSP

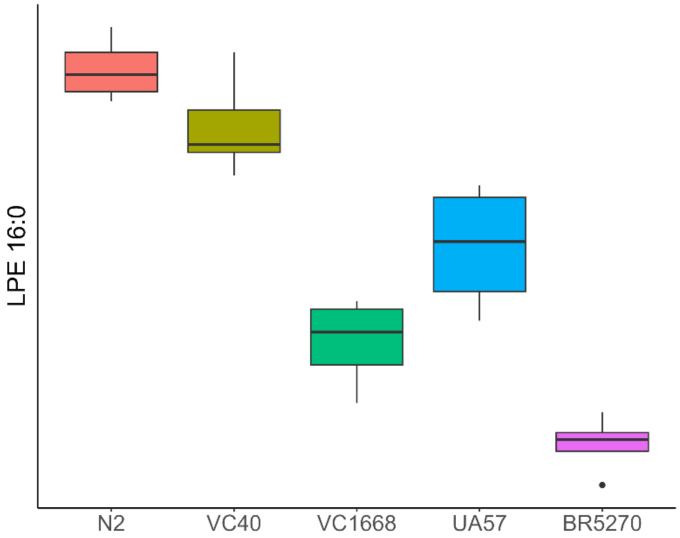

Pubchem CID: 89229
InChIKey: YVYMBNSKXOXSKW-UHFFFAOYSA-N
Title: [3-[2-Aminoethoxy(hydroxy)phosphoryl]ox
Compound name: LPE 16:0
Name used: LPE 16:0
Annotation level: 2a
ANOVA FDR PValue: 4.573e-07
Fisher's LSD pairwise comparison:
N2 > VC40 - Pvalue: 0.0417
N2 > VC1668 - Pvalue: 4.162e-08
N2 > UA57 - Pvalue: 1.620e-05
N2 > BR5270 - Pvalue: 2.390e-10
VC40 > VC1668 - Pvalue: 1.858e-07
VC40 > UA57 - Pvalue: 2.900e-04
VC40 > BR5270 - Pvalue: 3.752e-10
UA57 > VC1668 - Pvalue: 0.0066
VC1668 > BR5270 - Pvalue: 0.0039
UA57 > BR5270 - Pvalue: 4.458e-06
Type of analysis: RPLCNEG scheme1 MSDial PublicMSP

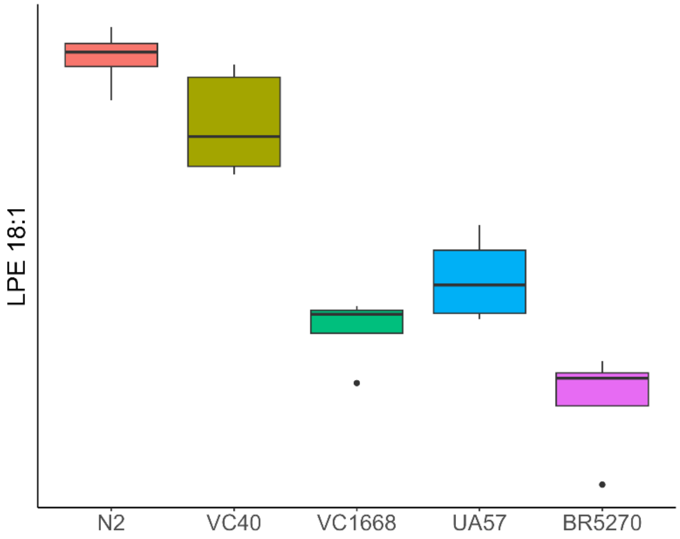

Pubchem CID:	25200684
InChIKey:	PYVRVRFVLRNJLY-UHFFFAOYSA-N
Title:	2-Azaniumylethyl (2-hydroxy-3-octadec-9-
Compound name:	LPE 18:1
Name used:	LPE 18:1
Annotation level:	2a
ANOVA FDR PValue:	1.134e-06
Fisher's LSD pairwise comparison:	N2 > VC40 - Pvalue: 0.0239<br>N2 > VC1668 - Pvalue: 5.112e-08<br>N2 > UA57 - Pvalue: 1.072e-06<br>N2 > BR5270 - Pvalue: 1.245e-09<br>VC40 > VC1668 - Pvalue: 3.998e-07<br>VC40 > UA57 - Pvalue: 1.829e-05<br>VC40 > BR5270 - Pvalue: 4.076e-09<br>VC1668 > BR5270 - Pvalue: 0.0351<br>UA57 > BR5270 - Pvalue: 9.857e-04
Type of analysis:	RPLCNEG scheme1 MSDial PublicMSP

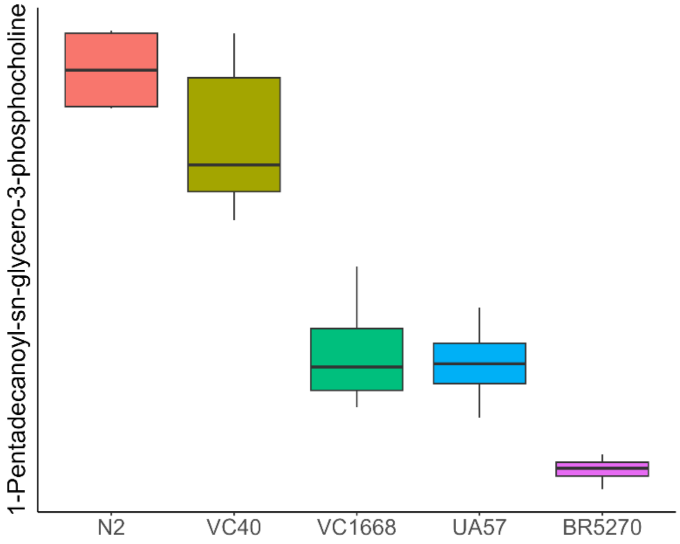

Pubchem CID:	24779458
InChIKey:	RJZVWDTYEWCUAR-JOCHJYFZSA-N
Title:	1-Pentadecanoyl-sn-glycero-3-phosphoch
Compound name:	1-pentadecanoyl-2-hydroxy-sn-glycero-3-[
Name used:	1-Pentadecanoyl-sn-glycero-3-phosphoch
Annotation level:	2a
ANOVA FDR PValue:	4.219e-06
Fisher's LSD pairwise comparison:	N2 > VC1668 - Pvalue: 7.807e-07<br>N2 > UA57 - Pvalue: 4.472e-07<br>N2 > BR5270 - Pvalue: 3.797e-09<br>VC40 > VC1668 - Pvalue: 5.506e-06<br>VC40 > UA57 - Pvalue: 2.775e-06<br>VC40 > BR5270 - Pvalue: 8.478e-09<br>VC1668 > BR5270 - Pvalue: 0.0072<br>UA57 > BR5270 - Pvalue: 0.0135
Type of analysis:	HILICPOS scheme1 MSDial PublicMSP
Other analyses in which significative:	HILICPOS scheme2 MSDial PublicMSP

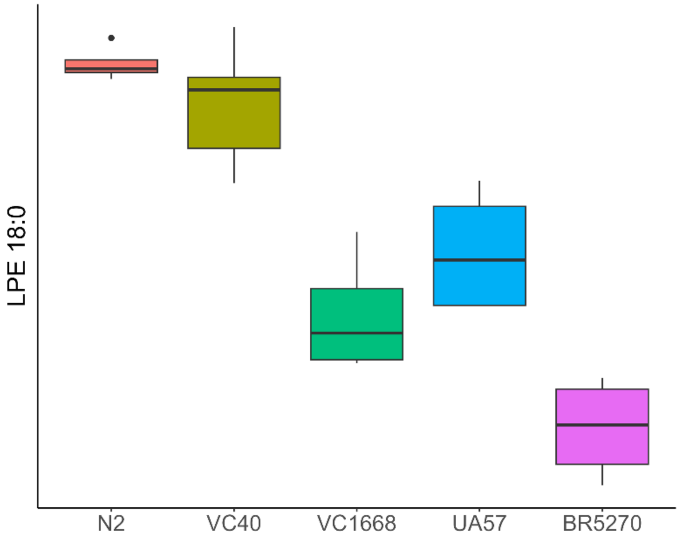

Pubchem CID:	45479288
InChIKey:	BBYWOYAFBUOUFP-UHFFFAOYSA-N
Title:	Stearoyl lysophosphatidylethanolamine
Compound name:	LPE 18:0
Name used:	LPE 18:0
Annotation level:	2a
ANOVA FDR PValue:	4.551e-06
Fisher's LSD pairwise comparison:	N2 > VC1668 - Pvalue: 1.883e-06<br>N2 > UA57 - Pvalue: 7.165e-05<br>N2 > BR5270 - Pvalue: 7.451e-09<br>VC40 > VC1668 - Pvalue: 2.639e-06<br>VC40 > UA57 - Pvalue: 1.882e-04<br>VC40 > BR5270 - Pvalue: 4.562e-09<br>VC1668 > BR5270 - Pvalue: 0.0068<br>UA57 > BR5270 - Pvalue: 1.427e-04
Type of analysis:	RPLCNEG scheme1 MSDial PublicMSP

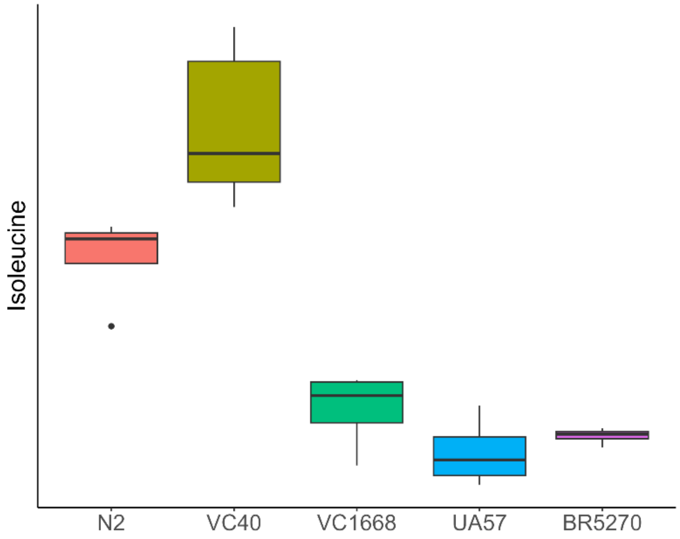

Pubchem CID:	791
InChIKey:	AGPKZVBTJJNPAG-UHFFFAOYSA-N
Title:	2-Amino-3-methylpentanoic acid
Compound name:	Isoleucine
Name used:	Isoleucine
Annotation level:	2a
ANOVA FDR PValue:	5.740e-06
Fisher's LSD pairwise comparison:	VC40 > N2 - Pvalue: 5.241e-04<br>N2 > VC1668 - Pvalue: 4.440e-04<br>N2 > UA57 - Pvalue: 2.916e-05<br>N2 > BR5270 - Pvalue: 8.095e-05<br>VC40 > VC1668 - Pvalue: 2.513e-08<br>VC40 > UA57 - Pvalue: 2.524e-09<br>VC40 > BR5270 - Pvalue: 5.888e-09
Type of analysis:	HILICPOS scheme2 MSDial PublicMSP

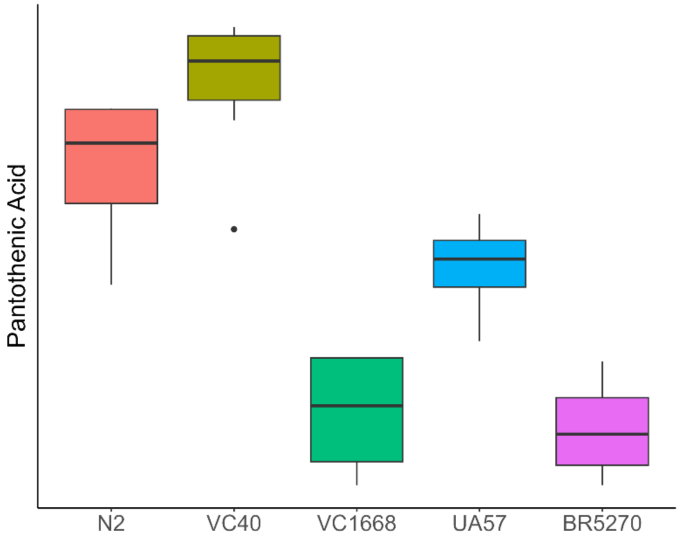

Pubchem CID:	6613
InChIKey:	GHOKWGTUZJEAQD-ZETCQYMHSA-N
Title:	Pantothenic Acid
Compound name:	3-[[(2R)-2,4-dihydroxy-3,3-dimethylbutano
Name used:	Pantothenic Acid
Annotation level:	2a
ANOVA FDR PValue:	1.609e-05
Fisher's LSD pairwise comparison:	VC40 > N2 - Pvalue: 0.0442<br>N2 > VC1668 - Pvalue: 4.987e-05<br>N2 > UA57 - Pvalue: 0.0489<br>N2 > BR5270 - Pvalue: 2.448e-05<br>VC40 > VC1668 - Pvalue: 1.223e-07<br>VC40 > UA57 - Pvalue: 2.023e-04<br>VC40 > BR5270 - Pvalue: 6.214e-08<br>UA57 > VC1668 - Pvalue: 0.0058<br>UA57 > BR5270 - Pvalue: 0.0028
Type of analysis:	HILICPOS scheme2 patRoon PubChemLi
Other analyses in which significative:	HILICPOS scheme2 patRoon WormJamE

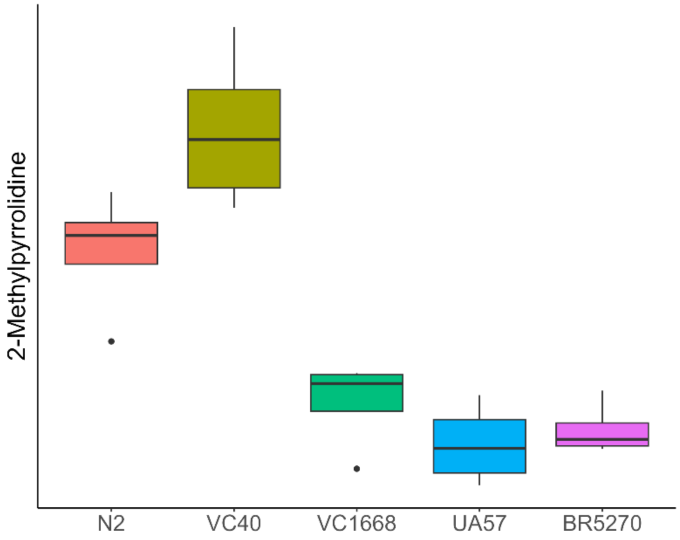

Pubchem CID:	13003
InChIKey:	RGHPCLZJAFCTIK-UHFFFAOYSA-N
Title:	2-Methylpyrrolidine
Compound name:	2-Methylpyrrolidine; CE10; RGHPCLZJAF
Name used:	2-Methylpyrrolidine
Annotation level:	2a
ANOVA FDR PValue:	2.212e-05
Fisher's LSD pairwise comparison:	VC40 > N2 - Pvalue: 0.0018<br>N2 > VC1668 - Pvalue: 8.720e-04<br>N2 > UA57 - Pvalue: 7.322e-05<br>N2 > BR5270 - Pvalue: 1.730e-04<br>VC40 > VC1668 - Pvalue: 1.192e-07<br>VC40 > UA57 - Pvalue: 1.305e-08<br>VC40 > BR5270 - Pvalue: 2.768e-08
Type of analysis:	HILICPOS scheme2 MSDial PublicMSP

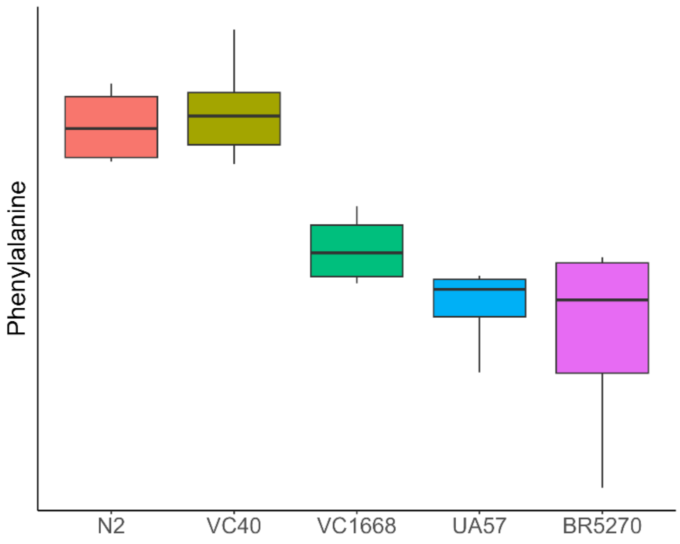

Pubchem CID:	6140
InChIKey:	COLNVLDHVKWLRT-QMMMGPOBSA-N
Title:	Phenylalanine
Compound name:	Phenylalanine
Name used:	Phenylalanine
Annotation level:	2a
ANOVA FDR PValue:	7.968e-04
Fisher's LSD pairwise comparison:	N2 > VC1668 - Pvalue: 0.0070<br>N2 > UA57 - Pvalue: 2.822e-04<br>N2 > BR5270 - Pvalue: 5.572e-05<br>VC40 > VC1668 - Pvalue: 0.0011<br>VC40 > UA57 - Pvalue: 2.796e-05<br>VC40 > BR5270 - Pvalue: 4.716e-06<br>VC1668 > BR5270 - Pvalue: 0.0456
Type of analysis:	RPLCNEG scheme1 MSDial WormJamEx
Other analyses in which significative:	RPLCNEG scheme1 MSDial PublicMSP<br>RPLCNEG scheme1 patRoon WormJamE<br>HILICPOS scheme1 MSDial PublicMSP<br>HILICPOS scheme1 MSDial WormJamEx

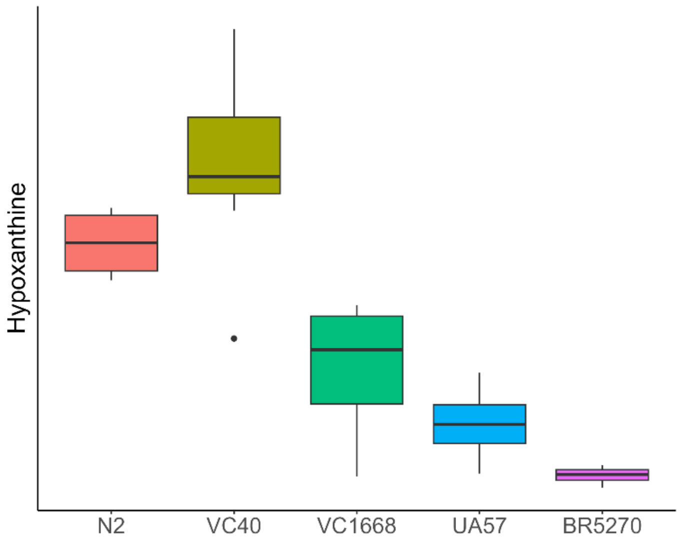

Pubchem CID:	790
InChIKey:	FDGQSTZJBFJUBT-UHFFFAOYSA-N
Title:	CID 790
Compound name:	Hypoxanthine
Name used:	Hypoxanthine
Annotation level:	2a
ANOVA FDR PValue:	0.0013
Fisher's LSD pairwise comparison:	N2 > VC1668 - Pvalue: 0.0161<br>N2 > UA57 - Pvalue: 0.0013<br>N2 > BR5270 - Pvalue: 1.165e-04<br>VC40 > VC1668 - Pvalue: 9.731e-05<br>VC40 > UA57 - Pvalue: 5.938e-06<br>VC40 > BR5270 - Pvalue: 4.865e-07<br>VC1668 > BR5270 - Pvalue: 0.0413
Type of analysis:	HILICPOS scheme2 MSDial PublicMSP
Other analyses in which significative:	HILICPOS scheme1 MSDial PublicMSP

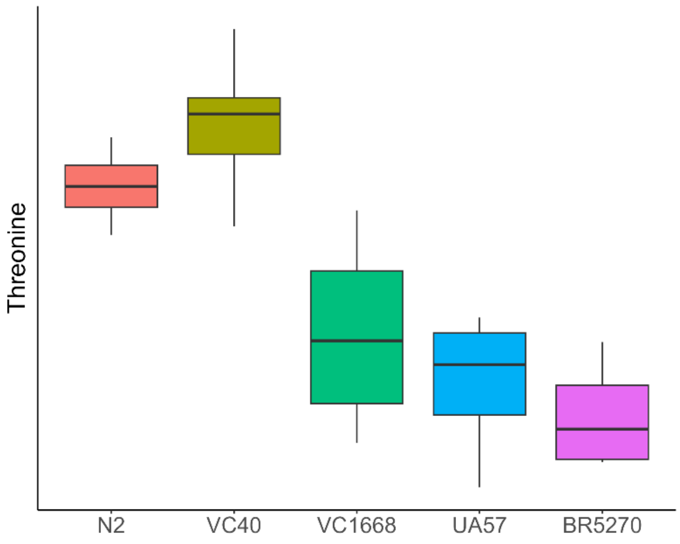

Pubchem CID:	6288
InChIKey:	AYFVYJQAPQTCCC-GBXIJSLDSA-N
Title:	Threonine
Compound name:	Threonine
Name used:	Threonine
Annotation level:	2a
ANOVA FDR PValue:	0.0014
Fisher's LSD pairwise comparison:	N2 > VC1668 - Pvalue: 0.0063<br>N2 > UA57 - Pvalue: 5.983e-04<br>N2 > BR5270 - Pvalue: 1.309e-04<br>VC40 > VC1668 - Pvalue: 7.147e-05<br>VC40 > UA57 - Pvalue: 5.373e-06<br>VC40 > BR5270 - Pvalue: 1.110e-06
Type of analysis:	HILICPOS scheme2 MSDial PublicMSP

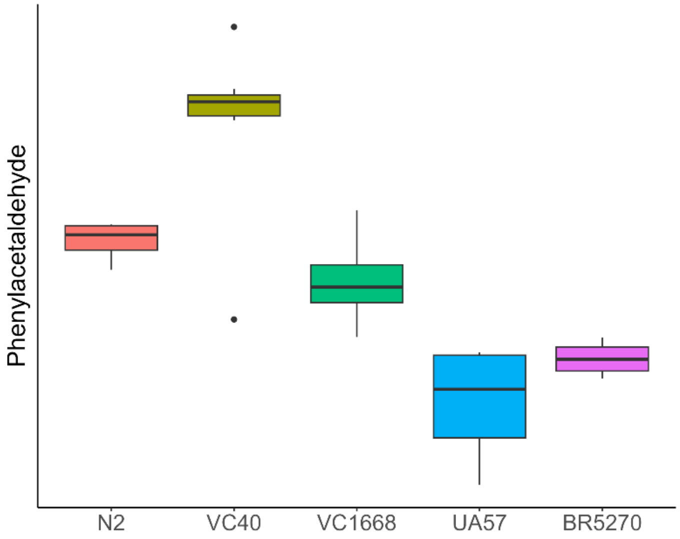

Pubchem CID:	998
InChIKey:	DTUQWGWMVIHBKE-UHFFFAOYSA-N
Title:	Phenylacetaldehyde
Compound name:	Phenylacetaldehyde
Name used:	Phenylacetaldehyde
Annotation level:	2a
ANOVA FDR PValue:	0.0019
Fisher's LSD pairwise comparison:	VC40 > N2 - Pvalue: 0.0052<br>N2 > UA57 - Pvalue: 0.0015<br>N2 > BR5270 - Pvalue: 0.0146<br>VC40 > VC1668 - Pvalue: 4.949e-04<br>VC40 > UA57 - Pvalue: 4.784e-07<br>VC40 > BR5270 - Pvalue: 5.241e-06<br>VC1668 > UA57 - Pvalue: 0.0110
Type of analysis:	HILICPOS scheme2 MSDial PublicMSP
Other analyses in which significative:	HILICPOS scheme1 MSDial PublicMSP

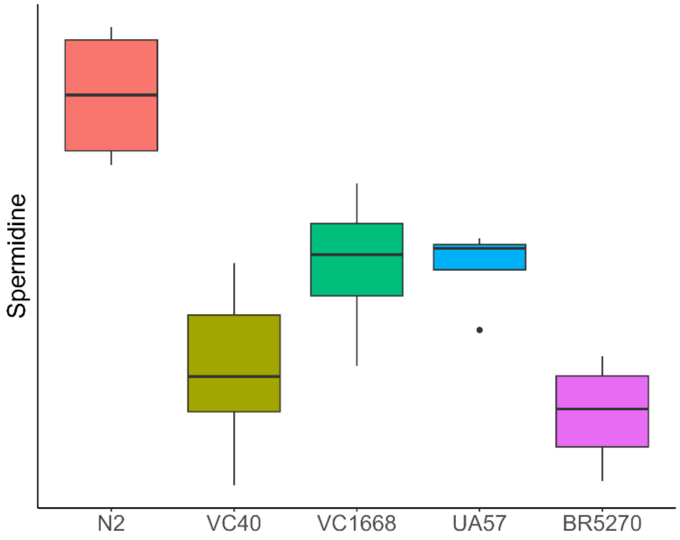

Pubchem CID:	1102
InChIKey:	ATHGHQPFGPMSJY-UHFFFAOYSA-N
Title:	Spermidine
Compound name:	SPERMIDINE
Name used:	Spermidine
Annotation level:	2a
ANOVA FDR PValue:	0.0033
Fisher's LSD pairwise comparison:	N2 > VC40 - Pvalue: 3.764e-06<br>N2 > VC1668 - Pvalue: 0.0026<br>N2 > UA57 - Pvalue: 0.0024<br>N2 > BR5270 - Pvalue: 2.982e-06<br>VC1668 > VC40 - Pvalue: 0.0264<br>UA57 > VC40 - Pvalue: 0.0284<br>VC1668 > BR5270 - Pvalue: 0.0065<br>UA57 > BR5270 - Pvalue: 0.0069
Type of analysis:	HILICPOS scheme1 MSDial PublicMSP
Other analyses in which significative:	HILICPOS scheme1 MSDial WormJamEx

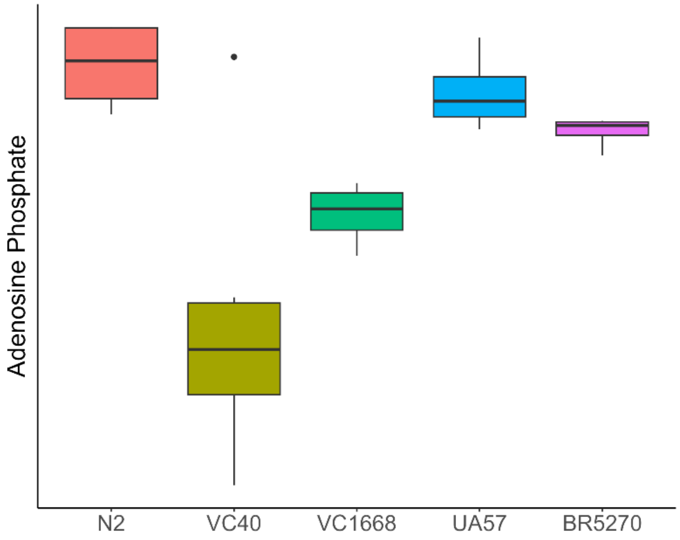

Pubchem CID:	6083
InChIKey:	UDMBCSSLTHHNCD-KQYNXXCUSA-N
Title:	Adenosine Phosphate
Compound name:	[(2R,3S,4R,5R)-5-(6-aminopurin-9-yl)-3,4-
Name used:	Adenosine Phosphate
Annotation level:	2a
ANOVA FDR PValue:	0.0047
Fisher's LSD pairwise comparison:	N2 > VC40 - Pvalue: 4.215e-05<br>N2 > VC1668 - Pvalue: 0.0185<br>VC1668 > VC40 - Pvalue: 0.0322<br>UA57 > VC40 - Pvalue: 1.381e-04<br>BR5270 > VC40 - Pvalue: 8.360e-04<br>UA57 > VC1668 - Pvalue: 0.0479
Type of analysis:	HILICPOS scheme2 patRoon PubChemLi
Other analyses in which significative:	HILICPOS scheme2 patRoon WormJamE

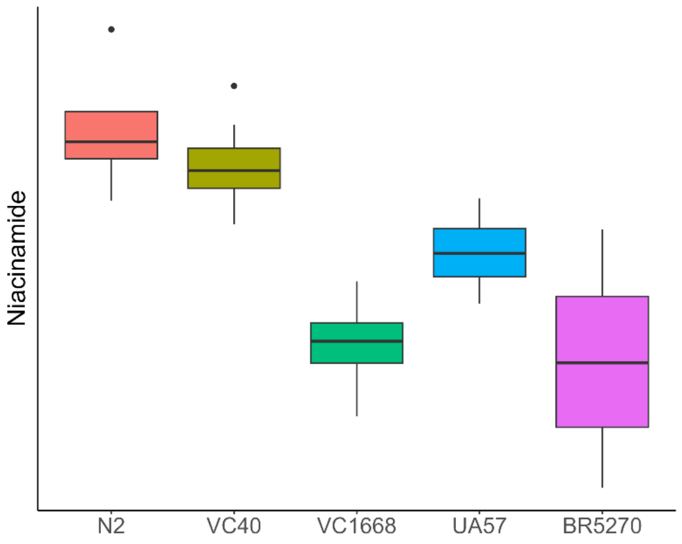

Pubchem CID:	936
InChIKey:	DFPAKSUCGFBDDF-UHFFFAOYSA-N
Title:	Niacinamide
Compound name:	Nicotinamide
Name used:	Niacinamide
Annotation level:	2a
ANOVA FDR PValue:	0.0101
Fisher's LSD pairwise comparison:	N2 > VC1668 - Pvalue: 1.750e-04<br>N2 > UA57 - Pvalue: 0.0156<br>N2 > BR5270 - Pvalue: 8.254e-05<br>VC40 > VC1668 - Pvalue: 2.867e-04<br>VC40 > UA57 - Pvalue: 0.0466<br>VC40 > BR5270 - Pvalue: 1.197e-04<br>UA57 > BR5270 - Pvalue: 0.0311
Type of analysis:	HILICPOS scheme1 MSDial PublicMSP
Other analyses in which significative:	HILICPOS scheme1 MSDial WormJamEx

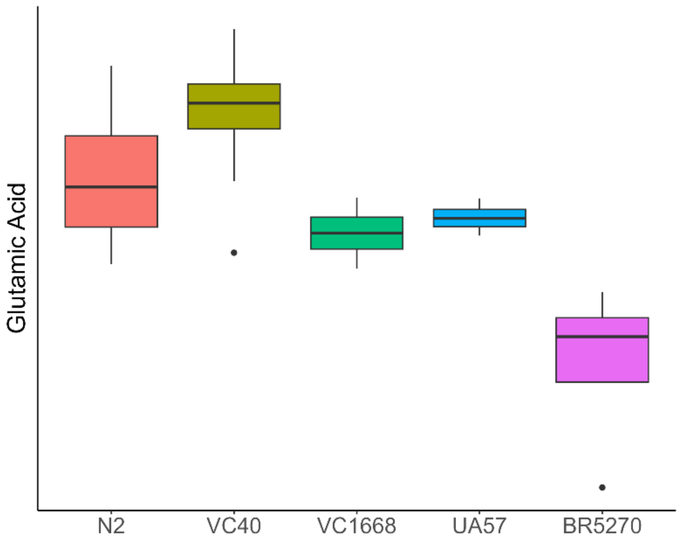

Pubchem CID:	33032
InChIKey:	WHUUTDBJXJRKMK-VKHMYHEASA-N
Title:	Glutamic Acid
Compound name:	L-Glutamic acid
Name used:	Glutamic Acid
Annotation level:	2a
ANOVA FDR PValue:	0.0112
Fisher's LSD pairwise comparison:	N2 > BR5270 - Pvalue: 7.299e-04<br>VC40 > VC1668 - Pvalue: 0.0092<br>VC40 > UA57 - Pvalue: 0.0209<br>VC40 > BR5270 - Pvalue: 6.846e-06<br>VC1668 > BR5270 - Pvalue: 0.0116<br>UA57 > BR5270 - Pvalue: 0.0056
Type of analysis:	RPLCNEG scheme1 MSDial PublicMSP
Other analyses in which significative:	RPLCNEG scheme1 MSDial WormJamEx

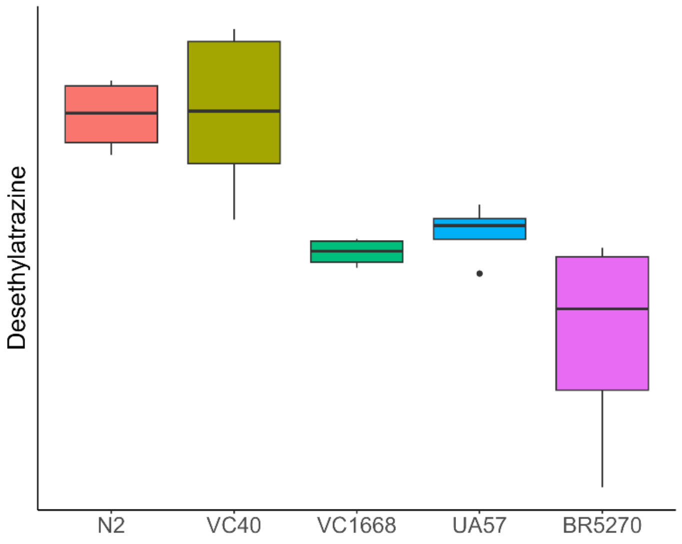

Pubchem CID:	22563
InChIKey:	DFWFIQKMSFGDCQ-UHFFFAOYSA-N
Title:	Desethyl atrazine
Compound name:	Desethylatrazine
Name used:	Desethylatrazine
Annotation level:	2a
ANOVA FDR PValue:	0.0131
Fisher's LSD pairwise comparison:	N2 > VC1668 - Pvalue: 0.0080<br>N2 > UA57 - Pvalue: 0.0204<br>N2 > BR5270 - Pvalue: 1.186e-04<br>VC40 > VC1668 - Pvalue: 0.0022<br>VC40 > UA57 - Pvalue: 0.0069<br>VC40 > BR5270 - Pvalue: 1.800e-05<br>UA57 > BR5270 - Pvalue: 0.0336
Type of analysis:	HILICPOS scheme1 MSDial PublicMSP

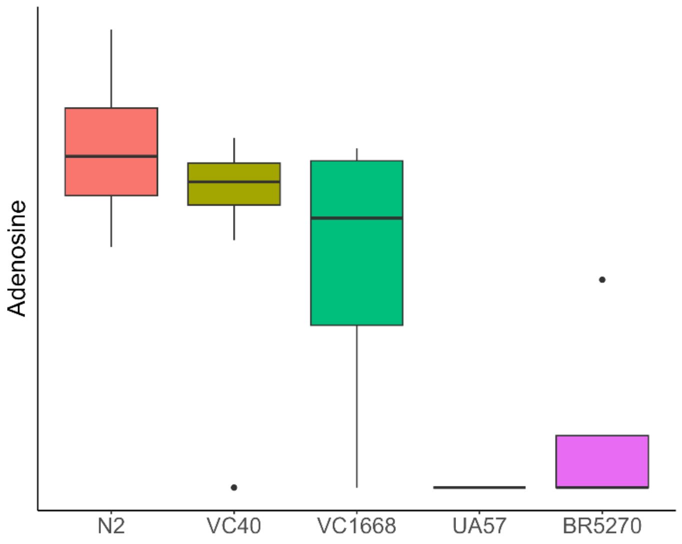

Pubchem CID:	60961
InChIKey:	OIRDTQYFTABQOQ-KQYNXXCUSA-N
Title:	Adenosine
Compound name:	(2R,3R,4S,5R)-2-(6-aminopurin-9-yl)-5-(hy
Name used:	Adenosine
Annotation level:	2a
ANOVA FDR PValue:	0.0147
Fisher's LSD pairwise comparison:	N2 > UA57 - Pvalue: 2.587e-04<br>N2 > BR5270 - Pvalue: 0.0012<br>VC40 > UA57 - Pvalue: 6.267e-04<br>VC40 > BR5270 - Pvalue: 0.0038<br>VC1668 > UA57 - Pvalue: 0.0094<br>VC1668 > BR5270 - Pvalue: 0.0400
Type of analysis:	HILICPOS scheme2 patRoon PubChemLi
Other analyses in which significative:	HILICPOS scheme2 patRoon WormJamE

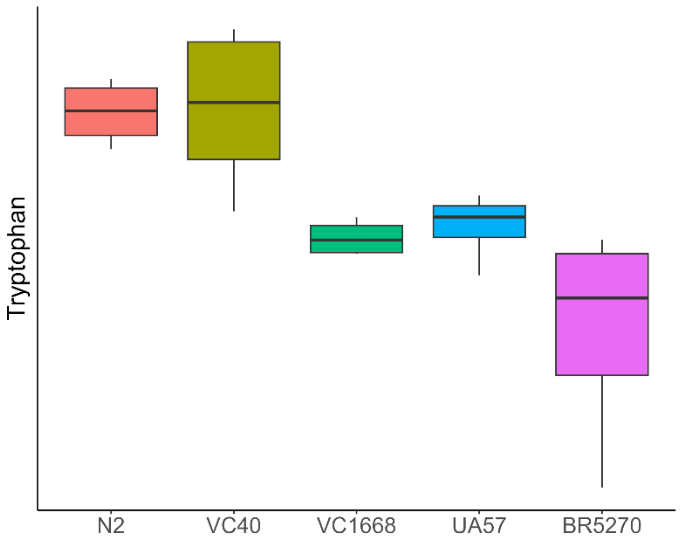

Pubchem CID:	9060
InChIKey:	QIVBCDIJIAJPQS-SECBINFHSA-N
Title:	D-Tryptophan
Compound name:	D-TRYPTOPHAN
Name used:	Tryptophan
Annotation level:	2a
ANOVA FDR PValue:	0.0173
Fisher's LSD pairwise comparison:	N2 > VC1668 - Pvalue: 0.0137<br>N2 > UA57 - Pvalue: 0.0235<br>N2 > BR5270 - Pvalue: 1.458e-04<br>VC40 > VC1668 - Pvalue: 0.0041<br>VC40 > UA57 - Pvalue: 0.0078<br>VC40 > BR5270 - Pvalue: 2.159e-05<br>UA57 > BR5270 - Pvalue: 0.0353
Type of analysis:	HILICPOS scheme1 MSDial PublicMSP
Other analyses in which significative:	HILICPOS scheme1 MSDial WormJamEx

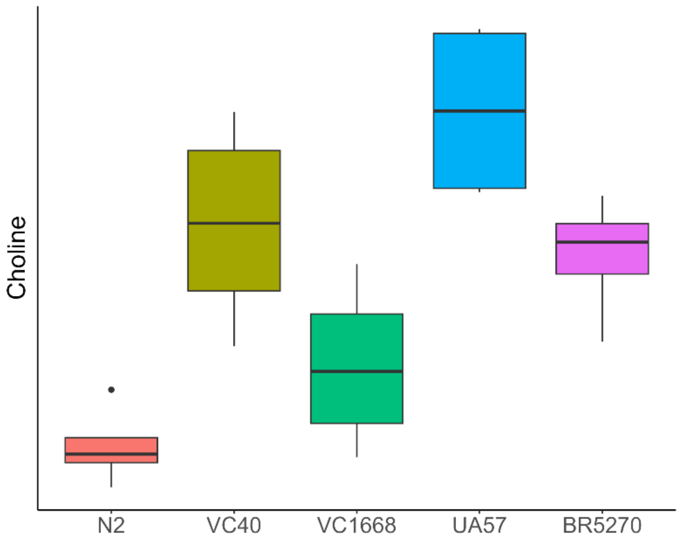

Pubchem CID:	305
InChIKey:	OEYIOHPDSNJKLS-UHFFFAOYSA-N
Title:	Choline
Compound name:	Choline
Name used:	Choline
Annotation level:	2a
ANOVA FDR PValue:	0.0175
Fisher's LSD pairwise comparison:	VC40 > N2 - Pvalue: 1.740e-04<br>UA57 > N2 - Pvalue: 7.845e-06<br>BR5270 > N2 - Pvalue: 0.0027<br>VC40 > VC1668 - Pvalue: 0.0077<br>UA57 > VC40 - Pvalue: 0.0297<br>UA57 > VC1668 - Pvalue: 1.896e-04<br>UA57 > BR5270 - Pvalue: 0.0171
Type of analysis:	HILICPOS scheme2 MSDial PublicMSP

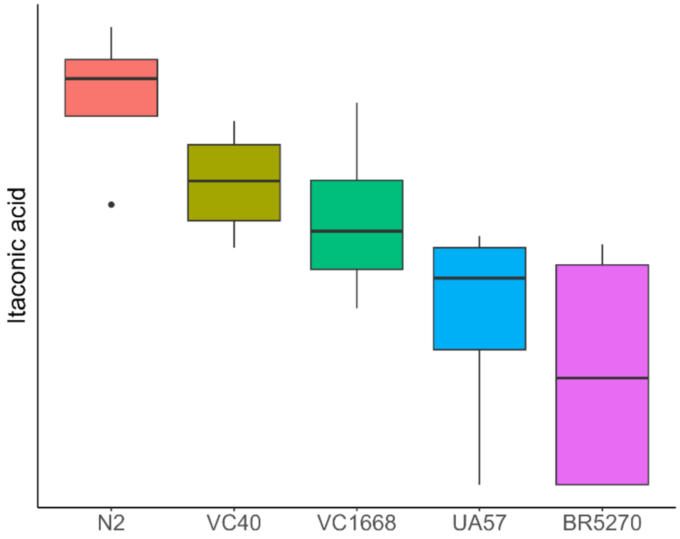

Pubchem CID:	811
InChIKey:	LVHBHZANLOWSRM-UHFFFAOYSA-N
Title:	Itaconic acid
Compound name:	2-methylidenebutanedioic acid
Name used:	Itaconic acid
Annotation level:	2a
ANOVA FDR PValue:	0.0179
Fisher's LSD pairwise comparison:	N2 > UA57 - Pvalue: 0.0020<br>N2 > BR5270 - Pvalue: 2.983e-04<br>VC40 > UA57 - Pvalue: 0.0202<br>VC40 > BR5270 - Pvalue: 0.0024<br>VC1668 > BR5270 - Pvalue: 0.0235
Type of analysis:	RPLCNEG scheme1 patRoon PubChemL
Other analyses in which significative:	RPLCNEG scheme1 patRoon WormJamE

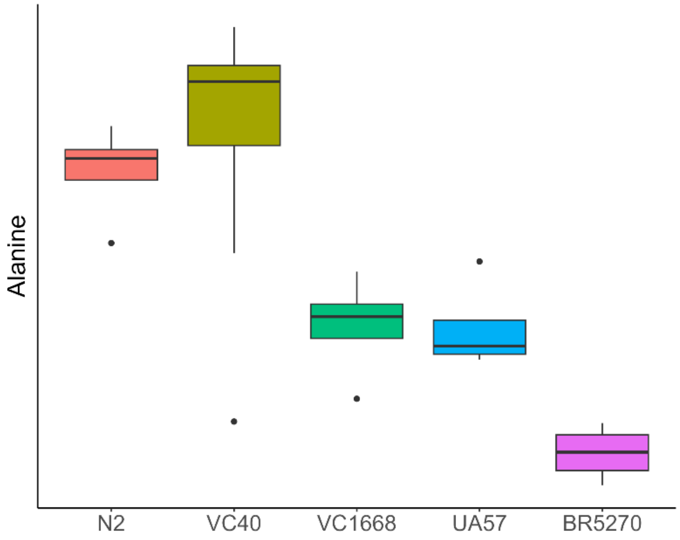

Pubchem CID:	71080
InChIKey:	QNAYBMKLOCPYGJ-UWTATZPHSA-N
Title:	D-Alanine
Compound name:	L-ALANINE
Name used:	Alanine
Annotation level:	2a
ANOVA FDR PValue:	0.0225
Fisher's LSD pairwise comparison:	N2 > VC1668 - Pvalue: 0.0241<br>N2 > UA57 - Pvalue: 0.0223<br>N2 > BR5270 - Pvalue: 2.638e-04<br>VC40 > VC1668 - Pvalue: 0.0025<br>VC40 > UA57 - Pvalue: 0.0023<br>VC40 > BR5270 - Pvalue: 1.360e-05
Type of analysis:	HILICPOS scheme2 MSDial PublicMSP
Other analyses in which significative:	RPLCNEG scheme1 MSDial PublicMSP

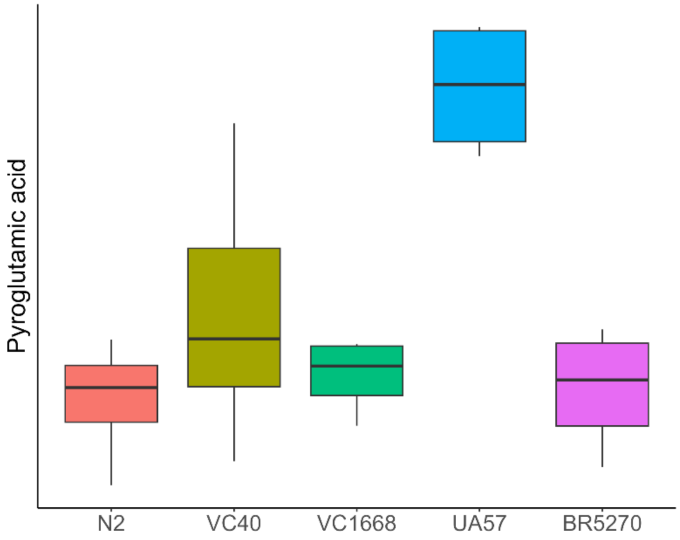

Pubchem CID:	7405
InChIKey:	ODHCTXKNWHHXJC-VKHMYHEASA-N
Title:	Pyroglutamic Acid
Compound name:	Pyroglutamic acid
Name used:	Pyroglutamic acid
Annotation level:	2a
ANOVA FDR PValue:	0.0267
Fisher's LSD pairwise comparison:	UA57 > N2 - Pvalue: 3.096e-05<br>UA57 > VC40 - Pvalue: 2.033e-04<br>UA57 > VC1668 - Pvalue: 7.937e-05<br>UA57 > BR5270 - Pvalue: 4.685e-05
Type of analysis:	HILICPOS scheme2 MSDial PublicMSP

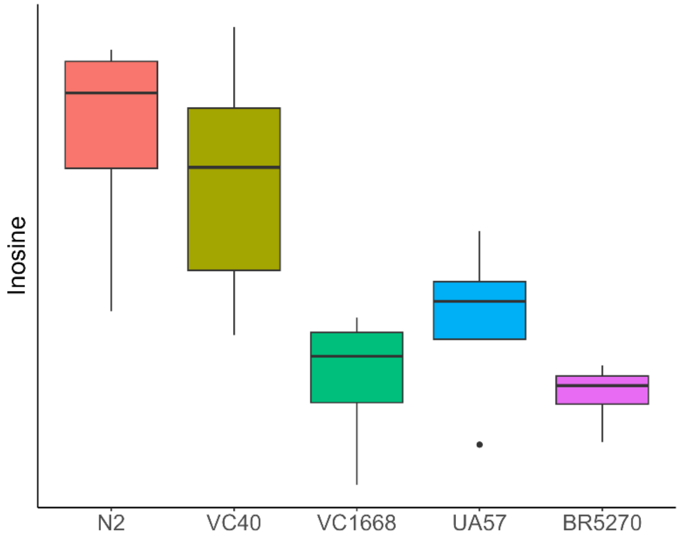

Pubchem CID:	135398641
InChIKey:	UGQMRVRMYYASKQ-KQYNXXCUSA-N
Title:	Inosine
Compound name:	9-[(2R,3R,4S,5R)-3,4-dihydroxy-5-(hydrox
Name used:	Inosine
Annotation level:	2a
ANOVA FDR PValue:	0.0301
Fisher's LSD pairwise comparison:	N2 > VC1668 - Pvalue: 0.0019<br>N2 > UA57 - Pvalue: 0.0135<br>N2 > BR5270 - Pvalue: 0.0011<br>VC40 > VC1668 - Pvalue: 0.0028<br>VC40 > UA57 - Pvalue: 0.0257<br>VC40 > BR5270 - Pvalue: 0.0015
Type of analysis:	HILICPOS scheme1 patRoon PubChemLi
Other analyses in which significative:	HILICPOS scheme1 patRoon WormJamE

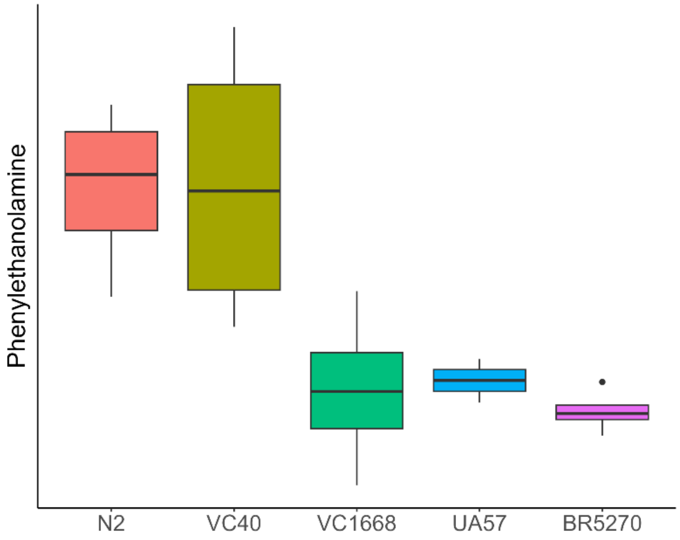

Pubchem CID:	1000
InChIKey:	ULSIYEODSMZIPX-UHFFFAOYSA-N
Title:	Phenylethanolamine
Compound name:	PHENYLETHANOLAMINE
Name used:	Phenylethanolamine
Annotation level:	2a
ANOVA FDR PValue:	0.0349
Fisher's LSD pairwise comparison:	N2 > VC1668 - Pvalue: 0.0038<br>N2 > UA57 - Pvalue: 0.0053<br>N2 > BR5270 - Pvalue: 0.0017<br>VC40 > VC1668 - Pvalue: 0.0011<br>VC40 > UA57 - Pvalue: 0.0016<br>VC40 > BR5270 - Pvalue: 4.269e-04
Type of analysis:	HILICPOS scheme1 MSDial PublicMSP

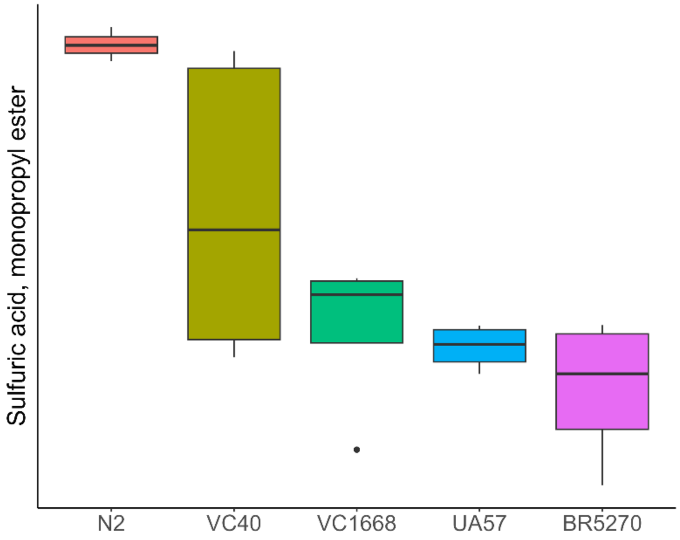

Pubchem CID:	70469
InChIKey:	TYRGSDXYMNTMML-UHFFFAOYSA-N
Title:	Sulfuric acid, monopropyl ester
Compound name:	Sulfuric acid, monopropyl ester
Name used:	Sulfuric acid, monopropyl ester
Annotation level:	2a
ANOVA FDR PValue:	0.0366
Fisher's LSD pairwise comparison:	N2 > VC40 - Pvalue: 0.0114<br>N2 > VC1668 - Pvalue: 5.437e-04<br>N2 > UA57 - Pvalue: 3.003e-04<br>N2 > BR5270 - Pvalue: 7.474e-05<br>VC40 > UA57 - Pvalue: 0.0336<br>VC40 > BR5270 - Pvalue: 0.0073
Type of analysis:	RPLCNEG scheme2 MSDial WormJamEx

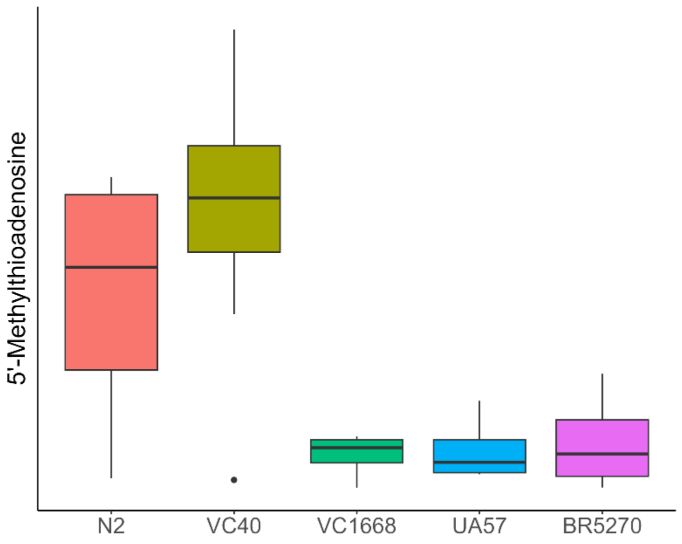

Pubchem CID:	439176
InChIKey:	WUUGFSXJNOTRMR-IOSLPCCCSA-N
Title:	5'-Methylthioadenosine
Compound name:	(2R,3R,4S,5S)-2-(6-aminopurin-9-yl)-5-(m
Name used:	5'-Methylthioadenosine
Annotation level:	2a
ANOVA FDR PValue:	0.0374
Fisher's LSD pairwise comparison:	N2 > VC1668 - Pvalue: 0.0487<br>VC40 > VC1668 - Pvalue: 0.0013<br>VC40 > UA57 - Pvalue: 0.0015<br>VC40 > BR5270 - Pvalue: 0.0020
Type of analysis:	HILICPOS scheme2 patRoon PubChemLi
Other analyses in which significative:	HILICPOS scheme2 patRoon WormJamE<br>HILICPOS scheme2 MSDial WormJamEx<br>HILICPOS scheme2 MSDial PublicMSP

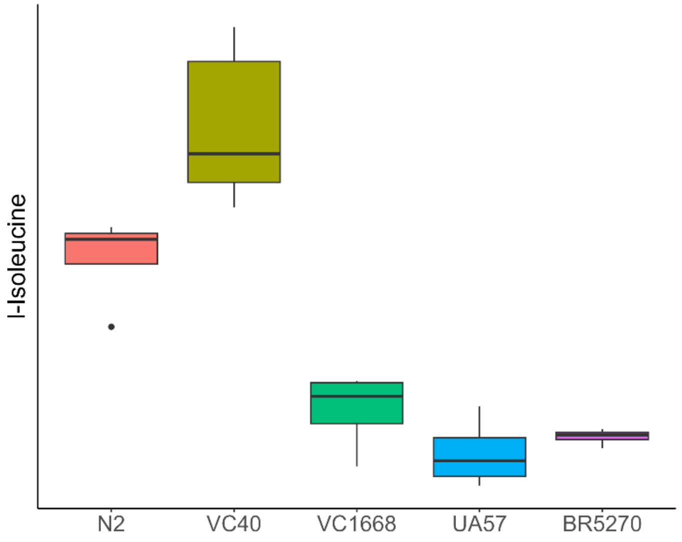

Pubchem CID:	6306
InChIKey:	AGPKZVBTJJNPAG-WHFBIAKZSA-N
Title:	l-Isoleucine
Compound name:	l-Isoleucine
Name used:	l-Isoleucine
Annotation level:	3a
ANOVA FDR PValue:	5.556e-06
Fisher's LSD pairwise comparison:	VC40 > N2 - Pvalue: 5.241e-04<br>N2 > VC1668 - Pvalue: 4.440e-04<br>N2 > UA57 - Pvalue: 2.916e-05<br>N2 > BR5270 - Pvalue: 8.095e-05<br>VC40 > VC1668 - Pvalue: 2.513e-08<br>VC40 > UA57 - Pvalue: 2.524e-09<br>VC40 > BR5270 - Pvalue: 5.888e-09
Type of analysis:	HILICPOS scheme2 MSDial WormJamEx

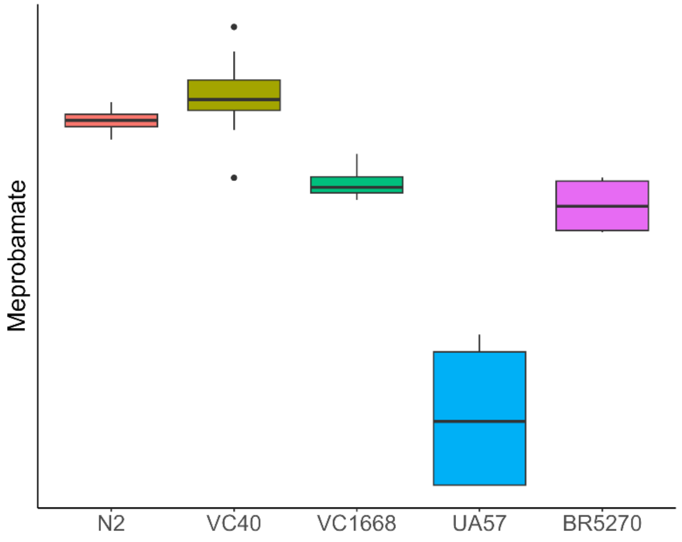

Pubchem CID:	4064
InChIKey:	NPPQSCRMBWNHMW-UHFFFAOYSA-N
Title:	Meprobamate
Compound name:	Meprobamate
Name used:	Meprobamate
Annotation level:	3a
ANOVA FDR PValue:	2.522e-05
Fisher's LSD pairwise comparison:	N2 > UA57 - Pvalue: 1.865e-08<br>N2 > BR5270 - Pvalue: 0.0156<br>VC40 > VC1668 - Pvalue: 0.0063<br>VC40 > UA57 - Pvalue: 5.256e-10<br>VC40 > BR5270 - Pvalue: 9.431e-04<br>VC1668 > UA57 - Pvalue: 6.251e-07<br>BR5270 > UA57 - Pvalue: 2.686e-06
Type of analysis:	HILICPOS scheme2 MSDial PublicMSP

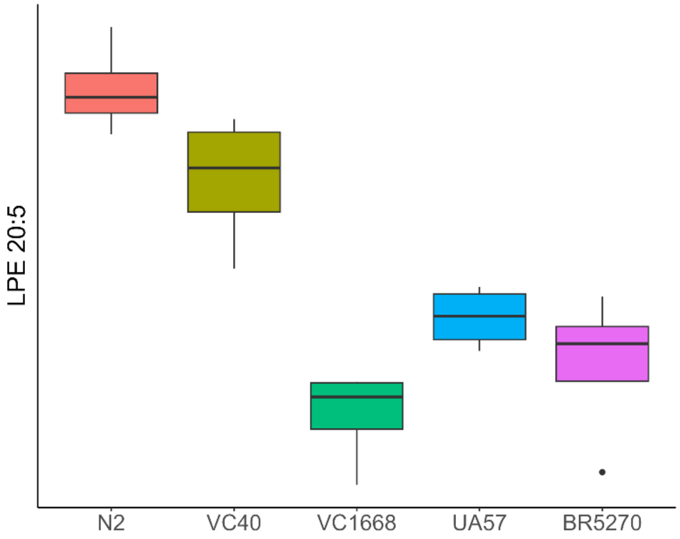

Pubchem CID:	75958074
InChIKey:	KCWGBXXAJHVZNN-UHFFFAOYSA-N
Title:	[3-[2-Aminoethoxy(hydroxy)phosphoryl]ox
Compound name:	LPE 20:5
Name used:	LPE 20:5
Annotation level:	3a
ANOVA FDR PValue:	3.045e-05
Fisher's LSD pairwise comparison:	N2 > VC40 - Pvalue: 0.0140<br>N2 > VC1668 - Pvalue: 4.871e-08<br>N2 > UA57 - Pvalue: 7.492e-06<br>N2 > BR5270 - Pvalue: 6.070e-07<br>VC40 > VC1668 - Pvalue: 6.137e-07<br>VC40 > UA57 - Pvalue: 3.695e-04<br>VC40 > BR5270 - Pvalue: 1.537e-05<br>UA57 > VC1668 - Pvalue: 0.0177
Type of analysis:	RPLCNEG scheme1 MSDial PublicMSP

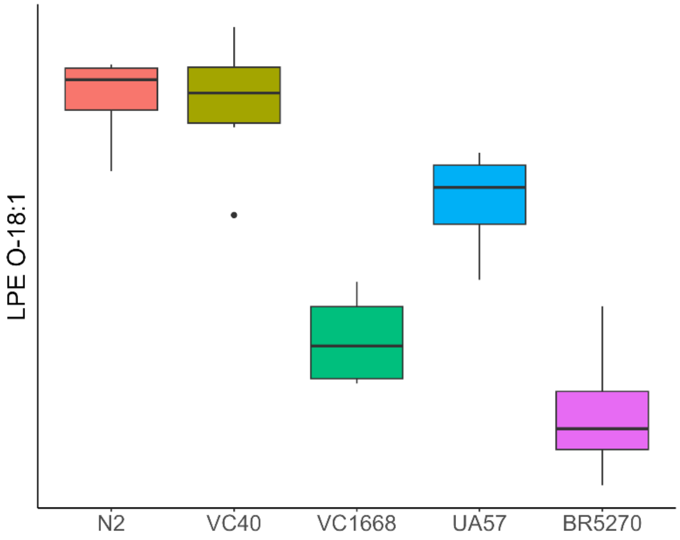

Pubchem CID:	74819130
InChIKey:	CDONWGCJDDHTLP-UHFFFAOYSA-N
Title:	CID 74819130
Compound name:	LPE O-18:1
Name used:	LPE O-18:1
Annotation level:	3a
ANOVA FDR PValue:	3.594e-05
Fisher's LSD pairwise comparison:	N2 > VC1668 - Pvalue: 1.175e-05<br>N2 > UA57 - Pvalue: 0.0209<br>N2 > BR5270 - Pvalue: 3.213e-07<br>VC40 > VC1668 - Pvalue: 2.037e-06<br>VC40 > UA57 - Pvalue: 0.0104<br>VC40 > BR5270 - Pvalue: 4.173e-08<br>UA57 > VC1668 - Pvalue: 0.0033<br>UA57 > BR5270 - Pvalue: 5.894e-05
Type of analysis:	RPLCNEG scheme1 MSDial PublicMSP

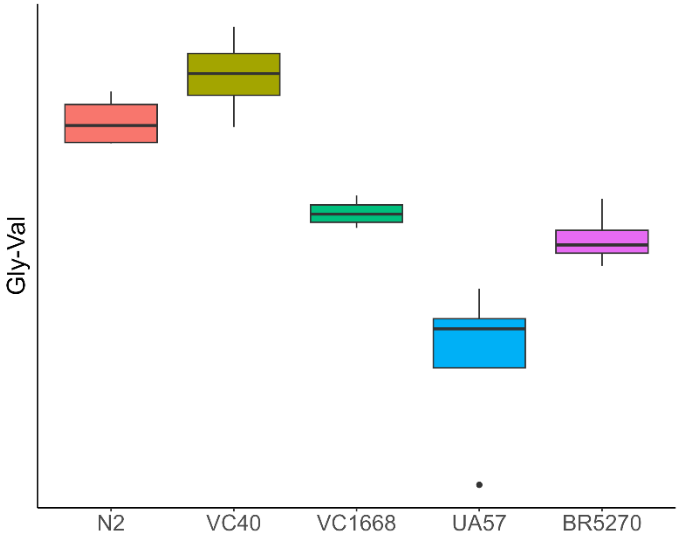

Pubchem CID:	97417
InChIKey:	STKYPAFSDFAEPH-UHFFFAOYSA-N
Title:	2-(2-Aminoacetamido)-3-methylbutanoic a
Compound name:	Gly-Val
Name used:	Gly-Val
Annotation level:	3a
ANOVA FDR PValue:	5.785e-05
Fisher's LSD pairwise comparison:	N2 > VC1668 - Pvalue: 0.0078<br>N2 > UA57 - Pvalue: 3.044e-07<br>N2 > BR5270 - Pvalue: 0.0012<br>VC40 > VC1668 - Pvalue: 6.217e-05<br>VC40 > UA57 - Pvalue: 2.219e-09<br>VC40 > BR5270 - Pvalue: 7.679e-06<br>VC1668 > UA57 - Pvalue: 1.532e-04<br>BR5270 > UA57 - Pvalue: 0.0010
Type of analysis:	HILICPOS scheme2 MSDial PublicMSP

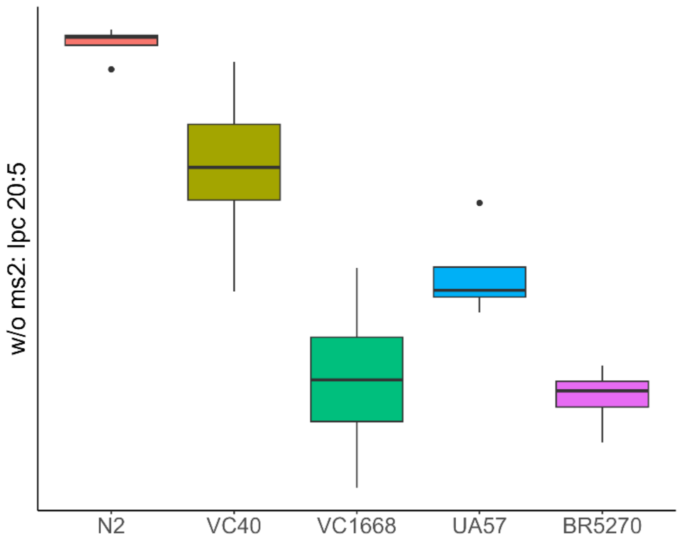

Pubchem CID:	73087697
InChIKey:	PDIGSOAOQOXRDU-UHFFFAOYSA-N
Title:	CID 73087697
Compound name:	w/o MS2: LPC 20:5
Name used:	w/o ms2: lpc 20:5
Annotation level:	3a
ANOVA FDR PValue:	7.871e-05
Fisher's LSD pairwise comparison:	N2 > VC40 - Pvalue: 0.0057<br>N2 > VC1668 - Pvalue: 4.179e-07<br>N2 > UA57 - Pvalue: 5.477e-05<br>N2 > BR5270 - Pvalue: 1.900e-07<br>VC40 > VC1668 - Pvalue: 2.285e-05<br>VC40 > UA57 - Pvalue: 0.0102<br>VC40 > BR5270 - Pvalue: 8.142e-06<br>UA57 > VC1668 - Pvalue: 0.0298<br>UA57 > BR5270 - Pvalue: 0.0123
Type of analysis:	RPLCNEG scheme1 MSDial PublicMSP

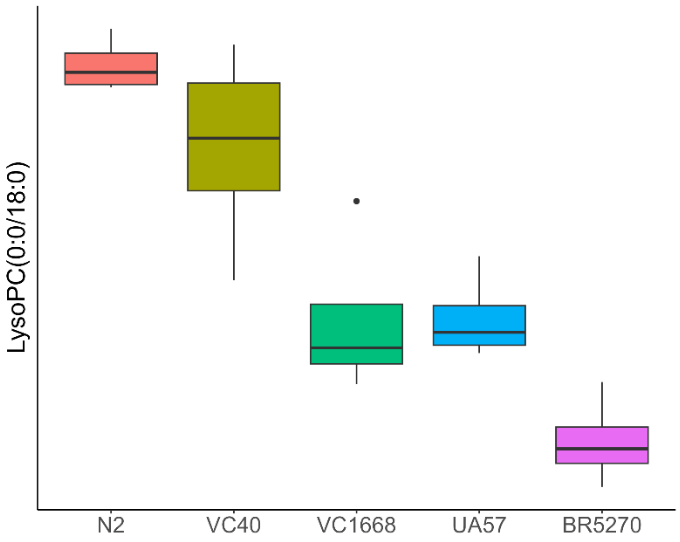

Pubchem CID:	24779491
InChIKey:	IQGPMZRCLCCXAG-RUZDIDTESA-N
Title:	2-Stearoyl-sn-glycero-3-phosphocholine
Compound name:	LysoPC(0:0/18:0)
Name used:	LysoPC(0:0/18:0)
Annotation level:	3a
ANOVA FDR PValue:	1.386e-04
Fisher's LSD pairwise comparison:	N2 > VC1668 - Pvalue: 2.780e-05<br>N2 > UA57 - Pvalue: 3.012e-05<br>N2 > BR5270 - Pvalue: 1.444e-07<br>VC40 > VC1668 - Pvalue: 3.528e-04<br>VC40 > UA57 - Pvalue: 3.884e-04<br>VC40 > BR5270 - Pvalue: 5.784e-07<br>VC1668 > BR5270 - Pvalue: 0.0174<br>UA57 > BR5270 - Pvalue: 0.0161
Type of analysis:	HILICPOS scheme1 MSDial PublicMSP
Other analyses in which significative:	HILICPOS scheme2 MSDial PublicMSP

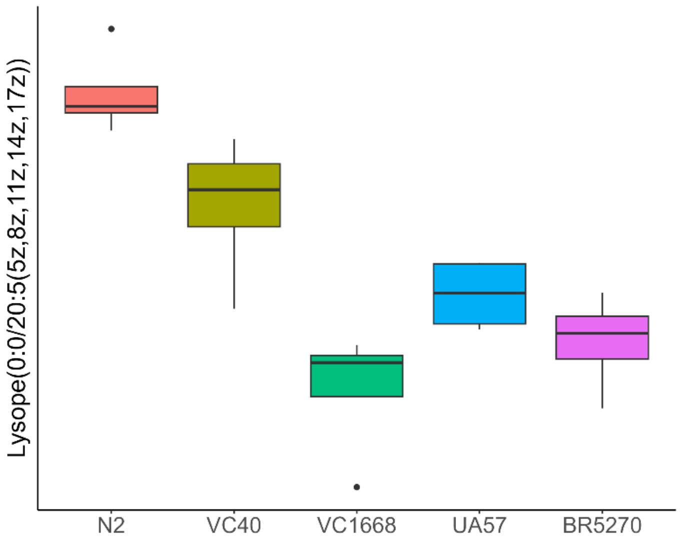

Pubchem CID:	53480938
InChIKey:	MMHCCHGAKPRCIO-KOYQJJOGSA-N
Title:	LysoPE(0:0/20:5(5Z,8Z,11Z,14Z,17Z))
Compound name:	LysoPE(0:0/20:5(5Z,8Z,11Z,14Z,17Z))
Name used:	Lysope(0:0/20:5(5z,8z,11z,14z,17z))
Annotation level:	3a
ANOVA FDR PValue:	2.079e-04
Fisher's LSD pairwise comparison:	N2 > VC40 - Pvalue: 0.0030<br>N2 > VC1668 - Pvalue: 1.447e-07<br>N2 > UA57 - Pvalue: 2.652e-05<br>N2 > BR5270 - Pvalue: 1.768e-06<br>VC40 > VC1668 - Pvalue: 1.050e-05<br>VC40 > UA57 - Pvalue: 0.0083<br>VC40 > BR5270 - Pvalue: 2.867e-04<br>UA57 > VC1668 - Pvalue: 0.0183
Type of analysis:	HILICPOS scheme1 MSDial WormJamEx[illegible]

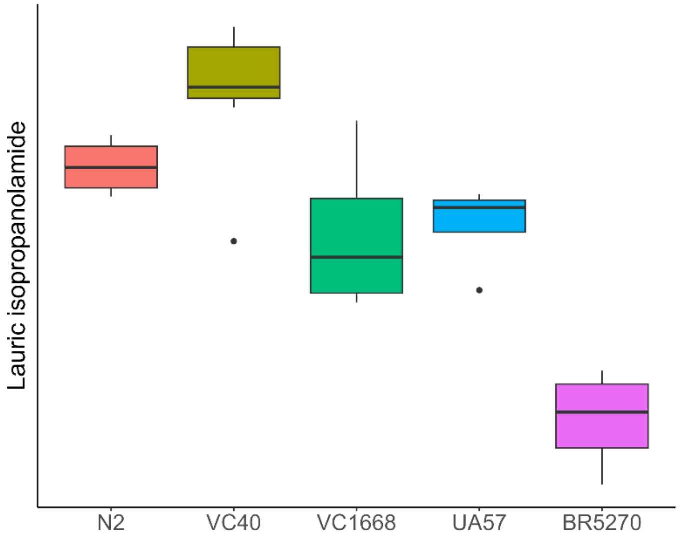

Pubchem CID:	9903249
InChIKey:	MMBILEWCGWTAOV-UHFFFAOYSA-N
Title:	Lauric isopropanolamide
Compound name:	Lauric isopropanolamide
Name used:	Lauric isopropanolamide
Annotation level:	3a
ANOVA FDR PValue:	3.183e-04
Fisher's LSD pairwise comparison:	N2 > BR5270 - Pvalue: 1.067e-05<br>VC40 > VC1668 - Pvalue: 0.0010<br>VC40 > UA57 - Pvalue: 0.0018<br>VC40 > BR5270 - Pvalue: 3.421e-08<br>VC1668 > BR5270 - Pvalue: 3.573e-04<br>UA57 > BR5270 - Pvalue: 2.146e-04
Type of analysis:	HILICPOS scheme1 MSDial PublicMSP

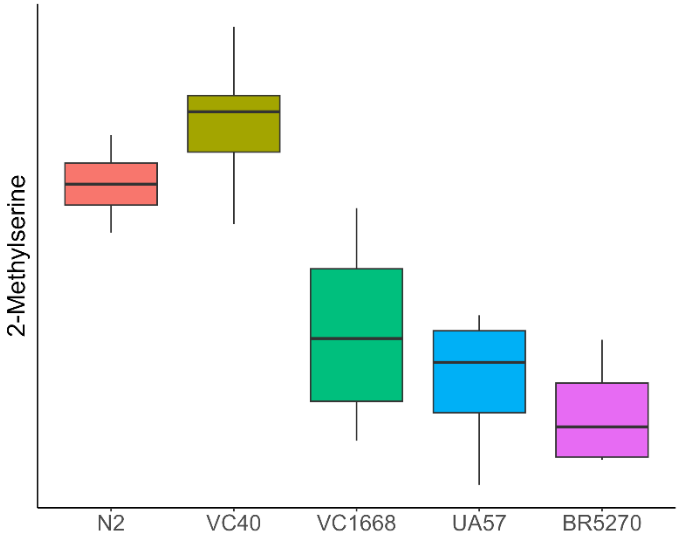

Pubchem CID:	94309
InChIKey:	CDUUKBXTEOFITR-UHFFFAOYSA-N
Title:	DL-2-Methylserine
Compound name:	2-Methylserine
Name used:	2-Methylserine
Annotation level:	3a
ANOVA FDR PValue:	0.0014
Fisher's LSD pairwise comparison:	N2 > VC1668 - Pvalue: 0.0063<br>N2 > UA57 - Pvalue: 5.983e-04<br>N2 > BR5270 - Pvalue: 1.309e-04<br>VC40 > VC1668 - Pvalue: 7.147e-05<br>VC40 > UA57 - Pvalue: 5.373e-06<br>VC40 > BR5270 - Pvalue: 1.110e-06
Type of analysis:	HILICPOS scheme2 MSDial WormJamEx

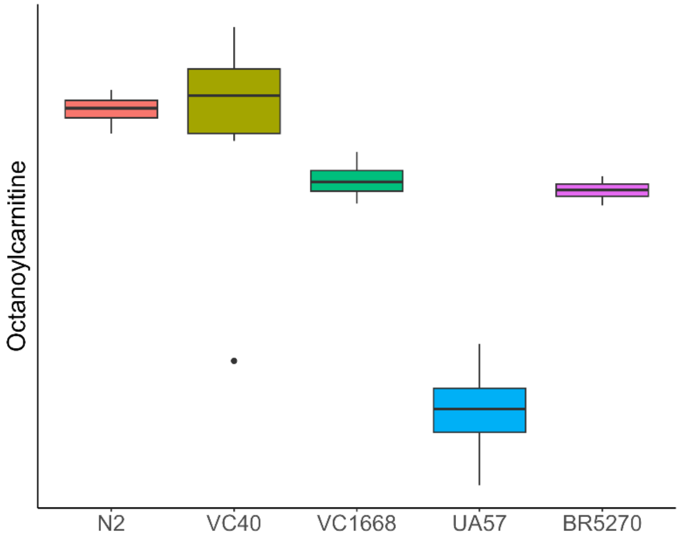

Pubchem CID:	11953814
InChIKey:	CXTATJFJDMJMIY-CYBMUJFWSA-N
Title:	L-Octanoylcarnitine
Compound name:	Octanoyl-L-Carnitine
Name used:	Octanoylcarnitine
Annotation level:	3a
ANOVA FDR PValue:	0.0076
Fisher's LSD pairwise comparison:	N2 > UA57 - Pvalue: 5.602e-06<br>VC40 > UA57 - Pvalue: 1.358e-06<br>VC1668 > UA57 - Pvalue: 1.295e-04<br>BR5270 > UA57 - Pvalue: 2.113e-04
Type of analysis:	HILICPOS scheme2 MSDial PublicMSP

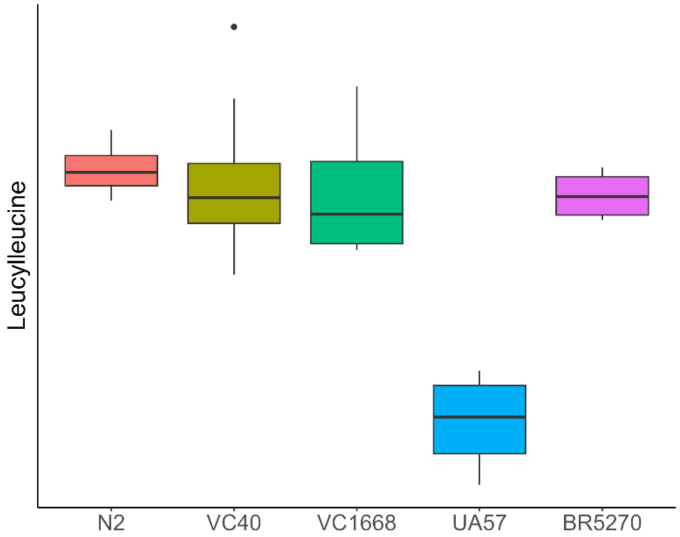

Pubchem CID:	94244
InChIKey:	LCPYQJIKPJDLLB-UHFFFAOYSA-N
Title:	Leucyl-leucine
Compound name:	Leucylleucine
Name used:	Leucylleucine
Annotation level:	3a
ANOVA FDR PValue:	0.0090
Fisher's LSD pairwise comparison:	N2 > UA57 - Pvalue: 2.264e-05<br>VC40 > UA57 - Pvalue: 7.523e-06<br>VC1668 > UA57 - Pvalue: 6.669e-05<br>BR5270 > UA57 - Pvalue: 8.102e-05
Type of analysis:	HILICPOS scheme1 MSDial PublicMSP

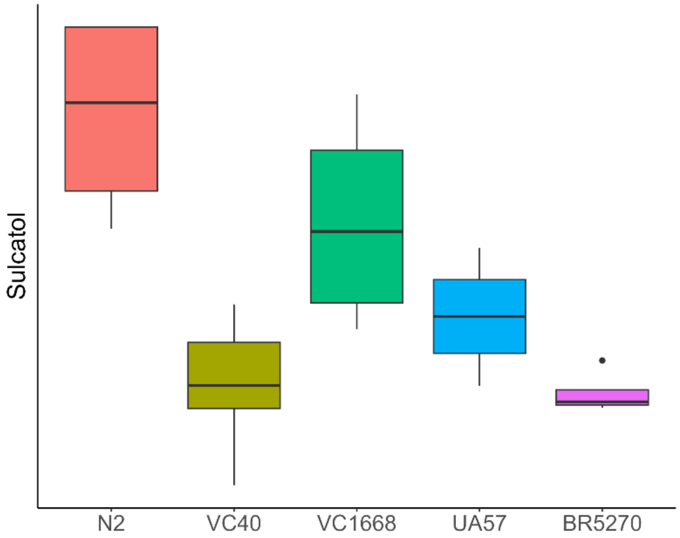

Pubchem CID:	20745
InChIKey:	OHEFFKYYKJVVOX-UHFFFAOYSA-N
Title:	Sulcatol
Compound name:	6-Methyl-5-hepten-2-ol
Name used:	Sulcatol
Annotation level:	3a
ANOVA FDR PValue:	0.0101
Fisher's LSD pairwise comparison:	N2 > VC40 - Pvalue: 1.262e-05<br>N2 > UA57 - Pvalue: 0.0011<br>N2 > BR5270 - Pvalue: 4.306e-05<br>VC1668 > VC40 - Pvalue: 0.0024<br>VC1668 > BR5270 - Pvalue: 0.0042
Type of analysis:	HILICPOS scheme1 MSDial PublicMSP

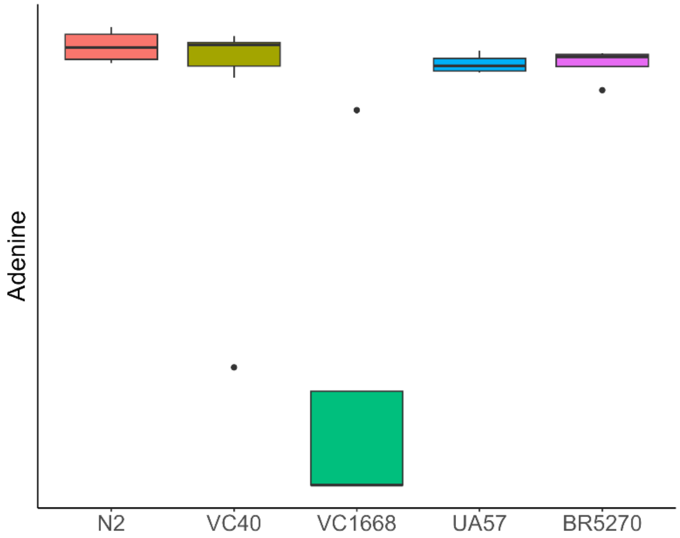

Pubchem CID:	190
InChIKey:	GFFGJBXGBJISGV-UHFFFAOYSA-N
Title:	Adenine
Compound name:	7H-purin-6-amine
Name used:	Adenine
Annotation level:	3a
ANOVA FDR PValue:	0.0113
Fisher's LSD pairwise comparison:	N2 > VC1668 - Pvalue: 1.266e-04<br>VC40 > VC1668 - Pvalue: 1.140e-04<br>UA57 > VC1668 - Pvalue: 2.185e-04<br>BR5270 > VC1668 - Pvalue: 2.223e-04
Type of analysis:	HILICPOS scheme2 patRoon PubChemLi
Other analyses in which significative:	HILICPOS scheme2 patRoon WormJamE

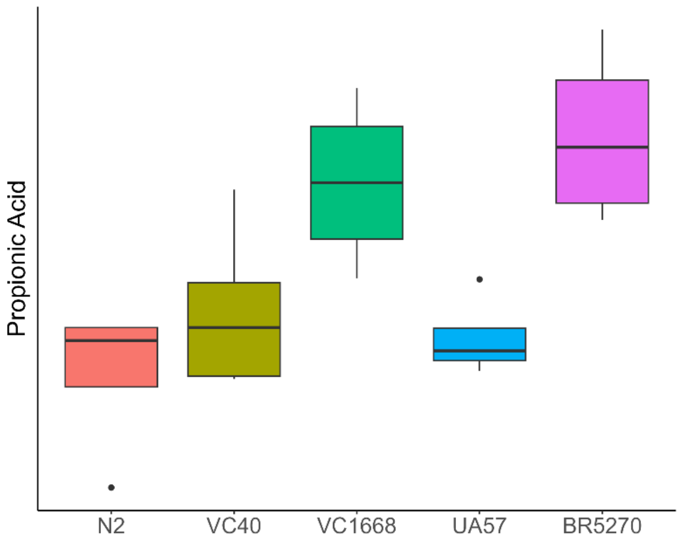

Pubchem CID:	1032
InChIKey:	XBDQKXXYIPTUBI-UHFFFAOYSA-N
Title:	Propionic Acid
Compound name:	Propionic Acid
Name used:	Propionic Acid
Annotation level:	3a
ANOVA FDR PValue:	0.0399
Fisher's LSD pairwise comparison:	VC1668 > N2 - Pvalue: 0.0015<br>BR5270 > N2 - Pvalue: 1.870e-04<br>VC1668 > VC40 - Pvalue: 0.0078<br>BR5270 > VC40 - Pvalue: 7.174e-04<br>VC1668 > UA57 - Pvalue: 0.0072<br>BR5270 > UA57 - Pvalue: 9.178e-04
Type of analysis:	HILICPOS scheme1 MSDial WormJamEx[illegible]

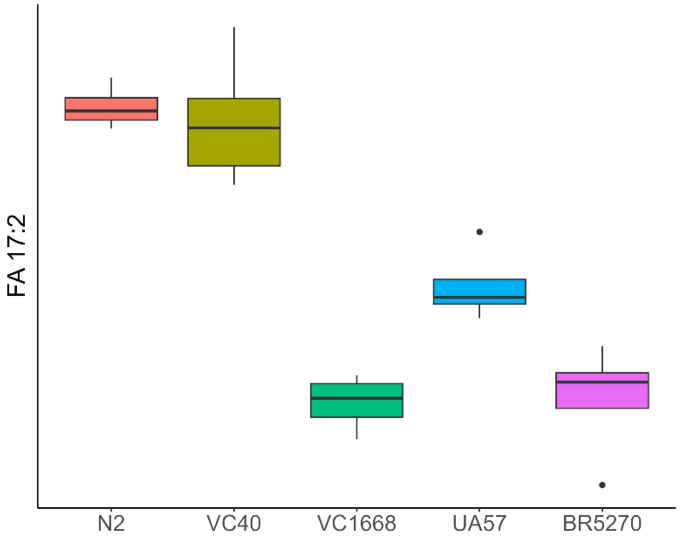

Pubchem CID:	54029176
InChIKey:	LEIXEEFBKOMCEQ-UHFFFAOYSA-N
Title:	Heptadeca-9,12-dienoic acid
Compound name:	FA 17:2
Name used:	FA 17:2
Annotation level:	3b
ANOVA FDR PValue:	1.642e-06
Fisher's LSD pairwise comparison:	N2 > VC1668 - Pvalue: 1.891e-08<br>N2 > UA57 - Pvalue: 2.146e-05<br>N2 > BR5270 - Pvalue: 2.310e-08<br>VC40 > VC1668 - Pvalue: 5.120e-09<br>VC40 > UA57 - Pvalue: 1.312e-05<br>VC40 > BR5270 - Pvalue: 6.372e-09<br>UA57 > VC1668 - Pvalue: 0.0018<br>UA57 > BR5270 - Pvalue: 0.0023
Type of analysis:	RPLCNEG scheme2 MSDial PublicMSP
Other analyses in which significative:	RPLCNEG scheme1 MSDial PublicMSP

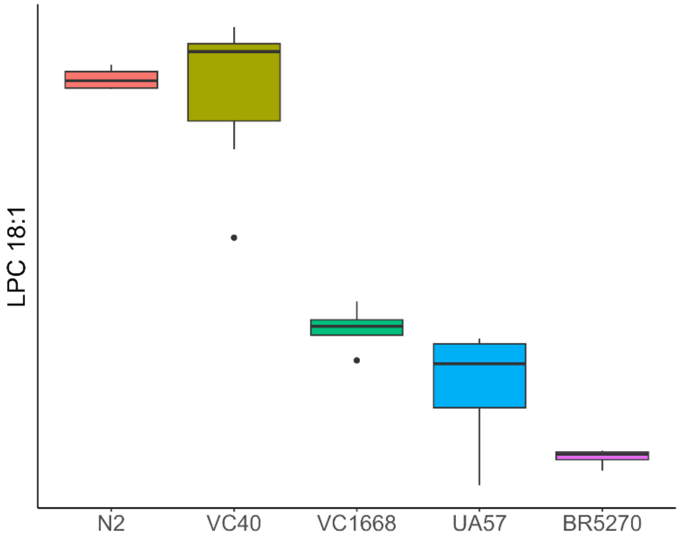

Pubchem CID:	3082003
InChIKey:	YAMUFBLWGFFICM-UHFFFAOYSA-N
Title:	(2-Hydroxy-3-octadec-9-enoyloxypropyl) 2
Compound name:	LPC 18:1
Name used:	LPC 18:1
Annotation level:	3b
ANOVA FDR PValue:	1.669e-06
Fisher's LSD pairwise comparison:	N2 > VC1668 - Pvalue: 2.251e-06<br>N2 > UA57 - Pvalue: 1.071e-07<br>N2 > BR5270 - Pvalue: 4.474e-09<br>VC40 > VC1668 - Pvalue: 5.457e-07<br>VC40 > UA57 - Pvalue: 1.980e-08<br>VC40 > BR5270 - Pvalue: 6.639e-10<br>VC1668 > BR5270 - Pvalue: 0.0028
Type of analysis:	HILICPOS scheme2 MSDial PublicMSP
Other analyses in which significative:	HILICPOS scheme1 MSDial PublicMSP

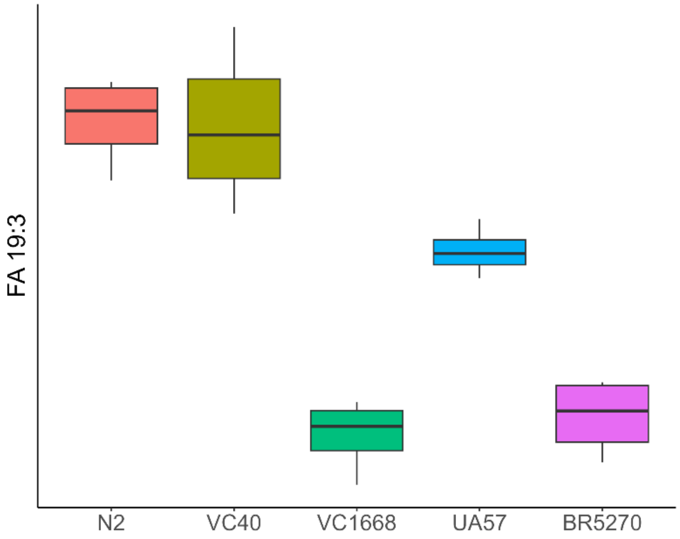

Pubchem CID:	74316061
InChIKey:	CNIDZCYLBMSLGW-UHFFFAOYSA-N
Title:	Nonadeca-10,13,16-trienoic acid
Compound name:	FA 19:3
Name used:	FA 19:3
Annotation level:	3b
ANOVA FDR PValue:	2.739e-06
Fisher's LSD pairwise comparison:	N2 > VC1668 - Pvalue: 2.995e-08<br>N2 > UA57 - Pvalue: 0.0015<br>N2 > BR5270 - Pvalue: 7.471e-08<br>VC40 > VC1668 - Pvalue: 4.299e-09<br>VC40 > UA57 - Pvalue: 6.562e-04<br>VC40 > BR5270 - Pvalue: 1.139e-08<br>UA57 > VC1668 - Pvalue: 4.596e-05<br>UA57 > BR5270 - Pvalue: 1.460e-04
Type of analysis:	RPLCNEG scheme2 MSDial PublicMSP
Other analyses in which significative:	RPLCNEG scheme1 MSDial PublicMSP

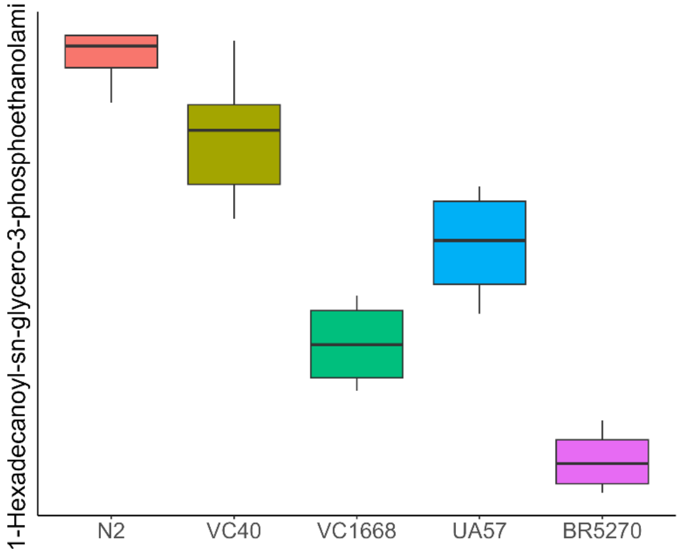

Pubchem CID:	9547069
InChIKey:	YVYMBNSKXOXSKW-HXUWFJFHSA-N
Title:	1-Hexadecanoyl-sn-glycero-3-phosphoeth
Compound name:	1-palmitoyl-2-hydroxy-sn-glycero-3-phospl
Name used:	1-Hexadecanoyl-sn-glycero-3-phosphoeth
Annotation level:	3b
ANOVA FDR PValue:	4.318e-06
Fisher's LSD pairwise comparison:	N2 > VC40 - Pvalue: 0.0247<br>N2 > VC1668 - Pvalue: 3.111e-07<br>N2 > UA57 - Pvalue: 7.396e-05<br>N2 > BR5270 - Pvalue: 1.549e-09<br>VC40 > VC1668 - Pvalue: 3.731e-06<br>VC40 > UA57 - Pvalue: 0.0032<br>VC40 > BR5270 - Pvalue: 5.186e-09<br>UA57 > VC1668 - Pvalue: 0.0162<br>VC1668 > BR5270 - Pvalue: 0.0058<br>UA57 > BR5270 - Pvalue: 1.541e-05
Type of analysis:	HILICPOS scheme1 MSDial PublicMSP
Other analyses in which significative:	HILICPOS scheme2 MSDial PublicMSP

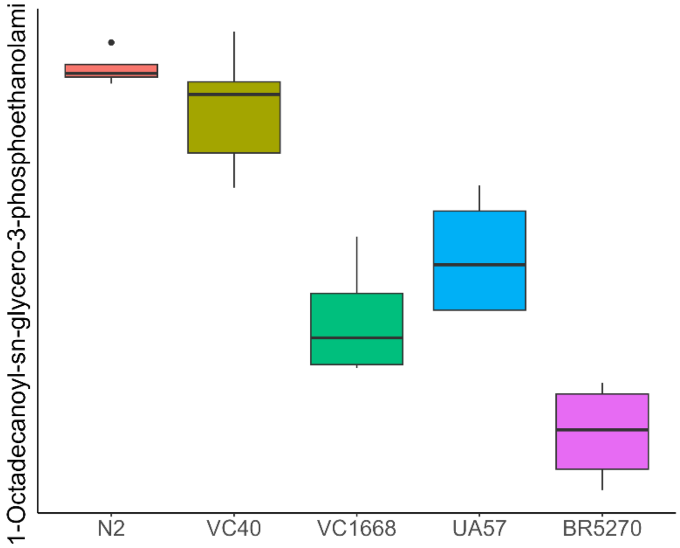

Pubchem CID:	9547068
InChIKey:	BBYWOYAFBUOUFP-JOCHJYFZSA-N
Title:	1-Octadecanoyl-sn-glycero-3-phosphoetha
Compound name:	1-Octadecanoyl-sn-glycero-3-phosphoetha
Name used:	1-Octadecanoyl-sn-glycero-3-phosphoetha
Annotation level:	3b
ANOVA FDR PValue:	4.500e-06
Fisher's LSD pairwise comparison:	N2 > VC1668 - Pvalue: 1.883e-06<br>N2 > UA57 - Pvalue: 7.165e-05<br>N2 > BR5270 - Pvalue: 7.451e-09<br>VC40 > VC1668 - Pvalue: 2.639e-06<br>VC40 > UA57 - Pvalue: 1.882e-04<br>VC40 > BR5270 - Pvalue: 4.562e-09<br>VC1668 > BR5270 - Pvalue: 0.0068<br>UA57 > BR5270 - Pvalue: 1.427e-04
Type of analysis:	RPLCNEG scheme1 MSDial WormJamEx

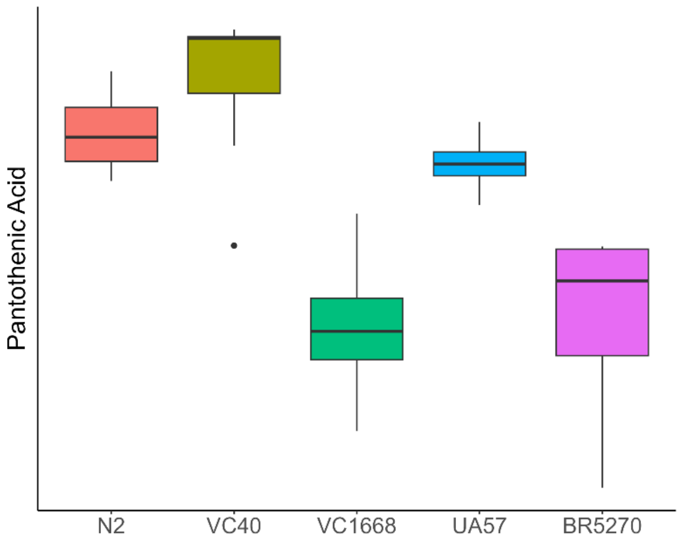

Pubchem CID: 6613
InChIKey: GHOKWGTUZJEAQD-ZETCQYMHSA-N
Title: Pantothenic Acid
Compound name: Pantothenate
Name used: Pantothenate
Annotation level: 3b
ANOVA FDR PValue: 0.0218
Fisher's LSD pairwise comparison: N2 > VC1668 - Pvalue: 0.0021
N2 > BR5270 - Pvalue: 0.0023
VC40 > VC1668 - Pvalue: 5.258e-05
VC40 > BR5270 - Pvalue: 5.992e-05
UA57 > VC1668 - Pvalue: 0.0078
UA57 > BR5270 - Pvalue: 0.0087
Type of analysis: HILICPOS scheme2 MSDial PublicMSP

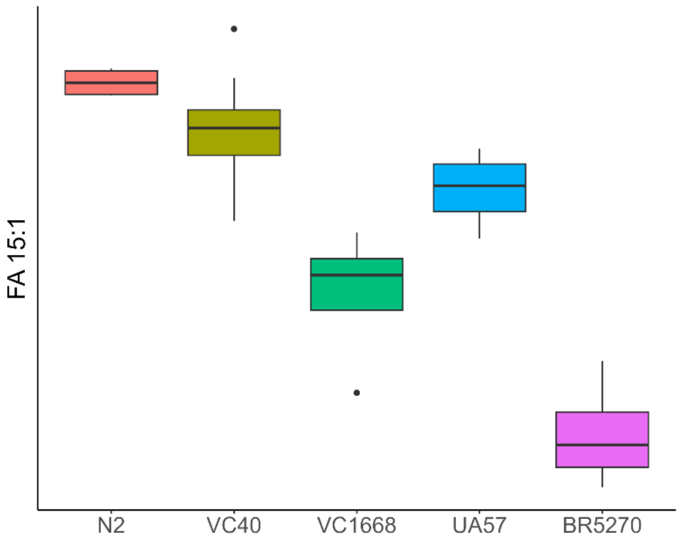

Pubchem CID: 3014053
InChIKey: DJCQJZKZUCHHAL-UHFFFAOYSA-N
Title: CID 3014053
Compound name: FA 15:1
Name used: FA 15:1
Annotation level: 3b
ANOVA FDR PValue: 1.665e-05
Fisher's LSD pairwise comparison: N2 > VC1668 - Pvalue: 1.720e-05
N2 > UA57 - Pvalue: 0.0093
N2 > BR5270 - Pvalue: 1.221e-08
VC40 > VC1668 - Pvalue: 5.819e-05
VC40 > BR5270 - Pvalue: 1.161e-08
UA57 > VC1668 - Pvalue: 0.0113
VC1668 > BR5270 - Pvalue: 0.0012
UA57 > BR5270 - Pvalue: 2.598e-06
Type of analysis: RPLCNEG scheme1 MSDial PublicMSP
Other analyses in which significative: RPLCNEG scheme2 MSDial PublicMSP

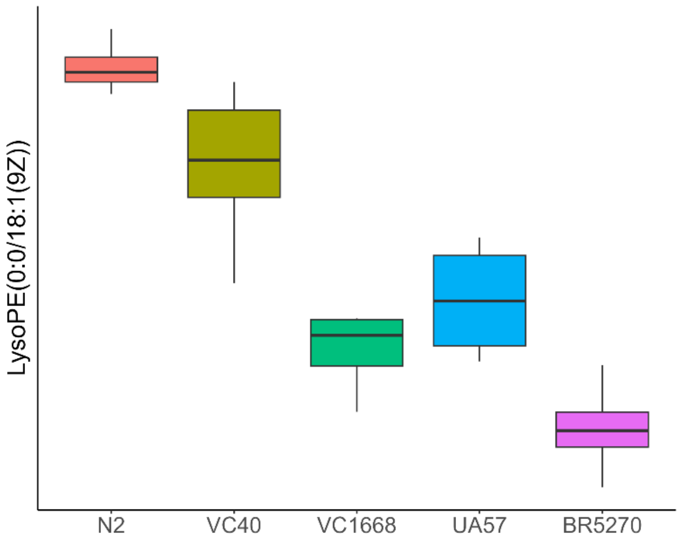

Pubchem CID: 53480925
InChIKey: NOVZJYYJYIFXJC-MZMPXXGTSA-N
Title: LysoPE(0:0/18:1(9Z))
Compound name: LysoPE(0:0/18:1(9Z))
Name used: LysoPE(0:0/18:1(9Z))
Annotation level: 3b
ANOVA FDR PValue: 2.649e-05
Fisher's LSD pairwise comparison: N2 > VC40 - Pvalue: 0.0095
N2 > VC1668 - Pvalue: 6.448e-07
N2 > UA57 - Pvalue: 8.885e-06
N2 > BR5270 - Pvalue: 1.660e-08
VC40 > VC1668 - Pvalue: 2.437e-05
VC40 > UA57 - Pvalue: 6.844e-04
VC40 > BR5270 - Pvalue: 2.180e-07
UA57 > BR5270 - Pvalue: 0.0038
Type of analysis: HILICPOS scheme1 MSDial WormJamEx

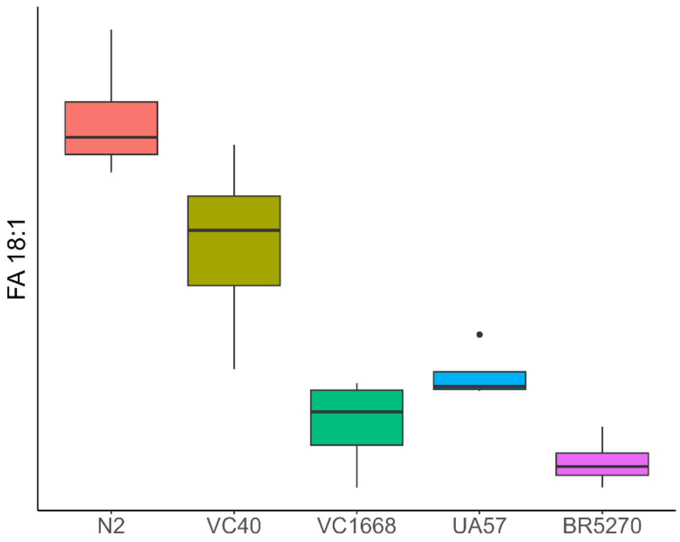

Pubchem CID:	965
InChIKey:	ZQPPMHVWECSIRJ-UHFFFAOYSA-N
Title:	9-Octadecenoic acid
Compound name:	FA 18:1
Name used:	FA 18:1
Annotation level:	3b
ANOVA FDR PValue:	4.872e-05
Fisher's LSD pairwise comparison:	N2 > VC40 - Pvalue: 0.0016<br>N2 > VC1668 - Pvalue: 2.572e-07<br>N2 > UA57 - Pvalue: 3.020e-06<br>N2 > BR5270 - Pvalue: 4.334e-08<br>VC40 > VC1668 - Pvalue: 4.049e-05<br>VC40 > UA57 - Pvalue: 0.0011<br>VC40 > BR5270 - Pvalue: 3.630e-06<br>UA57 > BR5270 - Pvalue: 0.0383
Type of analysis:	RPLCNEG scheme2 MSDial PublicMSP
Other analyses in which significative:	RPLCNEG scheme1 MSDial PublicMSP

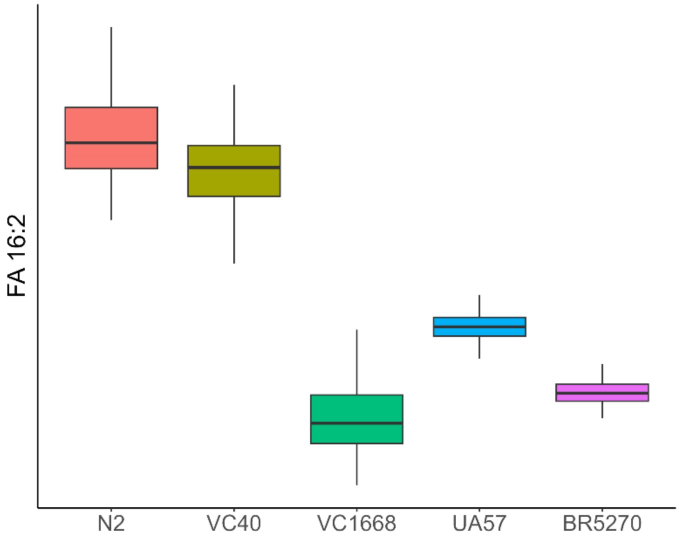

Pubchem CID:	3015085
InChIKey:	RVEKLXYYCHAMDF-UHFFFAOYSA-N
Title:	Hexadec-9,12-dienoic acid
Compound name:	FA 16:2
Name used:	FA 16:2
Annotation level:	3b
ANOVA FDR PValue:	5.678e-05
Fisher's LSD pairwise comparison:	N2 > VC1668 - Pvalue: 5.010e-07<br>N2 > UA57 - Pvalue: 6.470e-05<br>N2 > BR5270 - Pvalue: 1.661e-06<br>VC40 > VC1668 - Pvalue: 4.499e-07<br>VC40 > UA57 - Pvalue: 1.280e-04<br>VC40 > BR5270 - Pvalue: 1.795e-06<br>UA57 > VC1668 - Pvalue: 0.0310
Type of analysis:	RPLCNEG scheme2 MSDial PublicMSP
Other analyses in which significative:	RPLCNEG scheme1 MSDial PublicMSP

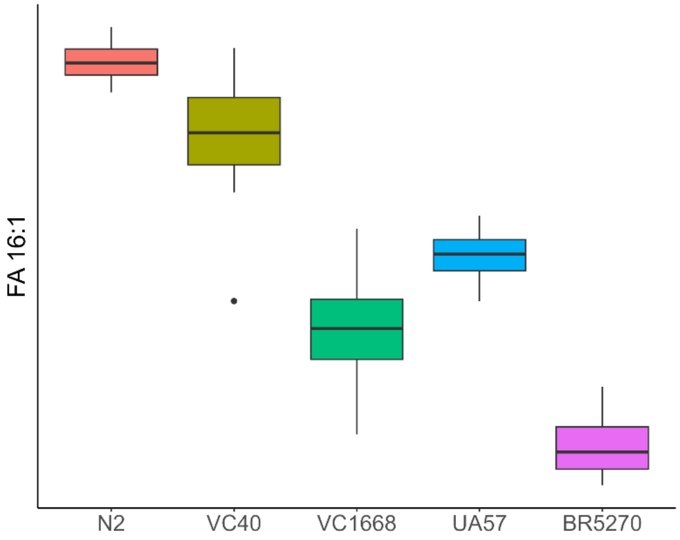

Pubchem CID:	4668
InChIKey:	SECPZKHBENQXJG-UHFFFAOYSA-N
Title:	9-Hexadecenoic acid
Compound name:	FA 16:1
Name used:	FA 16:1
Annotation level:	3b
ANOVA FDR PValue:	6.506e-05
Fisher's LSD pairwise comparison:	N2 > VC1668 - Pvalue: 9.132e-06<br>N2 > UA57 - Pvalue: 3.455e-04<br>N2 > BR5270 - Pvalue: 6.330e-08<br>VC40 > VC1668 - Pvalue: 1.174e-04<br>VC40 > UA57 - Pvalue: 0.0086<br>VC40 > BR5270 - Pvalue: 2.618e-07<br>VC1668 > BR5270 - Pvalue: 0.0199<br>UA57 > BR5270 - Pvalue: 5.018e-04
Type of analysis:	RPLCNEG scheme1 MSDial PublicMSP

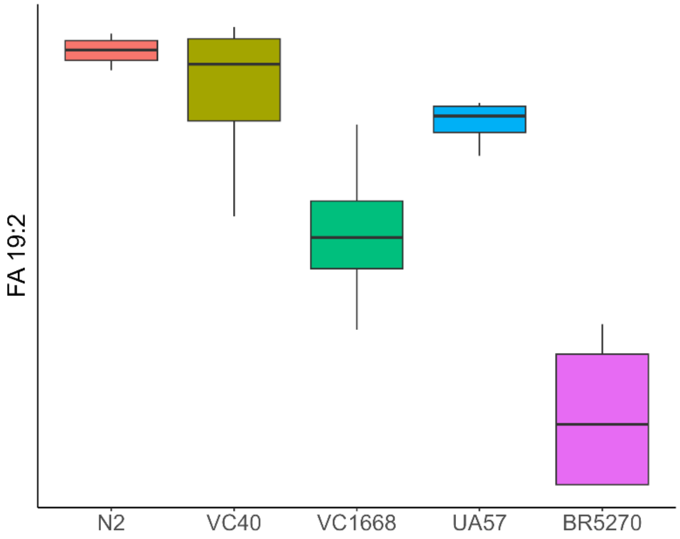

Pubchem CID:	53679925
InChIKey:	BFXHARLBAGKNRH-UHFFFAOYSA-N
Title:	Nonadeca-9,12-dienoic acid
Compound name:	FA 19:2
Name used:	FA 19:2
Annotation level:	3b
ANOVA FDR PValue:	6.928e-05
Fisher's LSD pairwise comparison:	N2 > VC1668 - Pvalue: 5.599e-04<br>N2 > BR5270 - Pvalue: 9.782e-08<br>VC40 > VC1668 - Pvalue: 0.0011<br>VC40 > BR5270 - Pvalue: 5.218e-08<br>UA57 > VC1668 - Pvalue: 0.0217<br>VC1668 > BR5270 - Pvalue: 5.370e-04<br>UA57 > BR5270 - Pvalue: 2.290e-06
Type of analysis:	RPLCNEG scheme1 MSDial PublicMSP

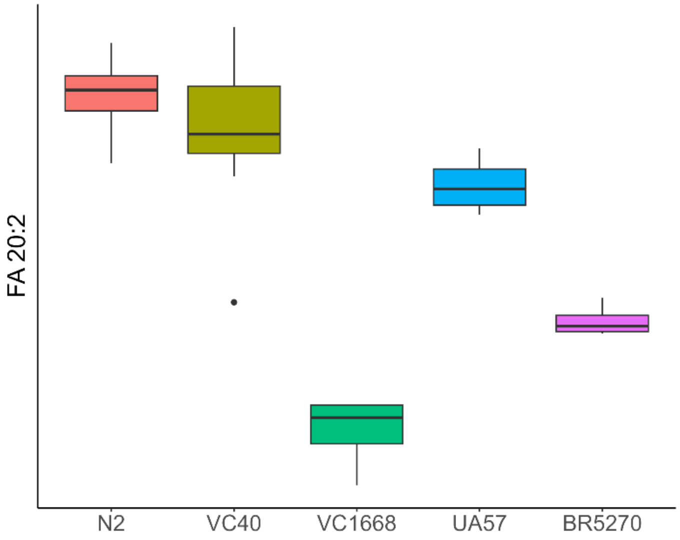

Pubchem CID:	3208
InChIKey:	XSXIVVZCUAHUJO-UHFFFAOYSA-N
Title:	Icosa-11,14-dienoic acid
Compound name:	FA 20:2
Name used:	FA 20:2
Annotation level:	3b
ANOVA FDR PValue:	8.584e-05
Fisher's LSD pairwise comparison:	N2 > VC1668 - Pvalue: 1.241e-07<br>N2 > UA57 - Pvalue: 0.0437<br>N2 > BR5270 - Pvalue: 2.820e-05<br>VC40 > VC1668 - Pvalue: 8.566e-08<br>VC40 > BR5270 - Pvalue: 4.504e-05<br>UA57 > VC1668 - Pvalue: 8.948e-06<br>BR5270 > VC1668 - Pvalue: 0.0144<br>UA57 > BR5270 - Pvalue: 0.0037
Type of analysis:	RPLCNEG scheme2 MSDial PublicMSP
Other analyses in which significative:	RPLCNEG scheme1 MSDial PublicMSP

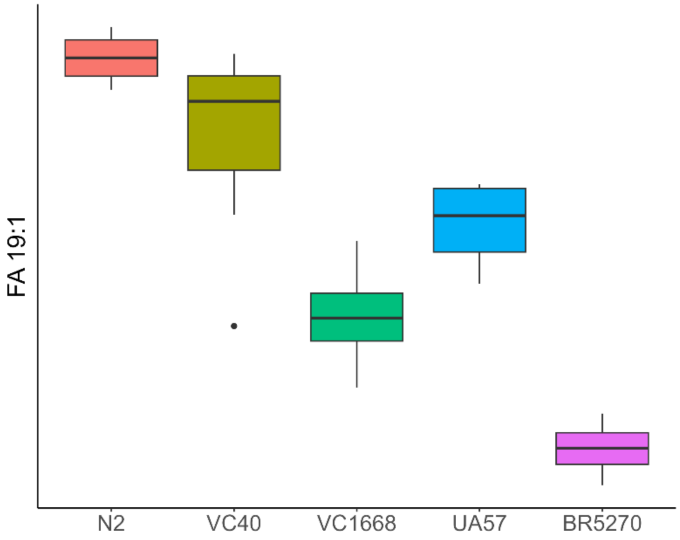

Pubchem CID:	54518967
InChIKey:	YOKHLRHWEXTWJR-UHFFFAOYSA-N
Title:	Nonadec-9-enoic acid
Compound name:	FA 19:1
Name used:	FA 19:1
Annotation level:	3b
ANOVA FDR PValue:	9.841e-05
Fisher's LSD pairwise comparison:	N2 > VC1668 - Pvalue: 2.598e-05<br>N2 > UA57 - Pvalue: 0.0021<br>N2 > BR5270 - Pvalue: 9.035e-08<br>VC40 > VC1668 - Pvalue: 3.012e-04<br>VC40 > UA57 - Pvalue: 0.0440<br>VC40 > BR5270 - Pvalue: 3.070e-07<br>VC1668 > BR5270 - Pvalue: 0.0107<br>UA57 > BR5270 - Pvalue: 1.299e-04
Type of analysis:	RPLCNEG scheme1 MSDial PublicMSP
Other analyses in which significative:	RPLCNEG scheme2 MSDial PublicMSP

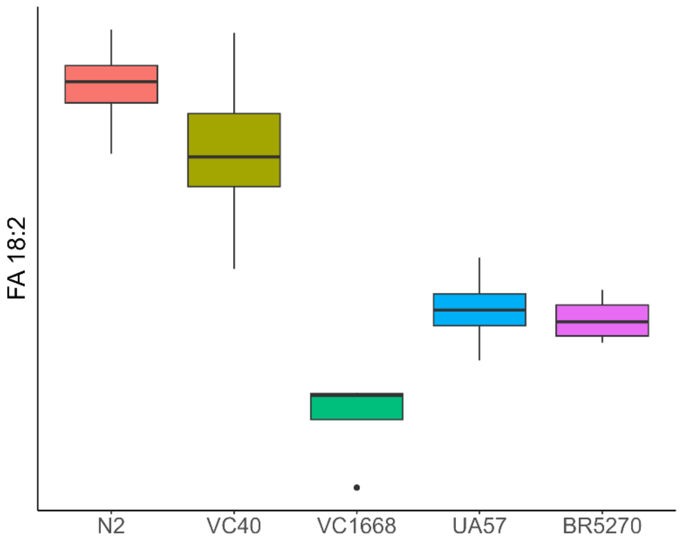

Pubchem CID:	3931
InChIKey:	OYHQOLUKZRVURQ-UHFFFAOYSA-N
Title:	9,12-Octadecadienoic Acid
Compound name:	FA 18:2
Name used:	FA 18:2
Annotation level:	3b
ANOVA FDR PValue:	1.211e-04
Fisher's LSD pairwise comparison:	N2 > VC1668 - Pvalue: 1.953e-07<br>N2 > UA57 - Pvalue: 3.836e-05<br>N2 > BR5270 - Pvalue: 2.326e-05<br>VC40 > VC1668 - Pvalue: 4.742e-07<br>VC40 > UA57 - Pvalue: 2.678e-04<br>VC40 > BR5270 - Pvalue: 1.474e-04<br>UA57 > VC1668 - Pvalue: 0.0180<br>BR5270 > VC1668 - Pvalue: 0.0291
Type of analysis:	RPLCNEG scheme2 MSDial PublicMSP
Other analyses in which significative:	RPLCNEG scheme1 MSDial PublicMSP

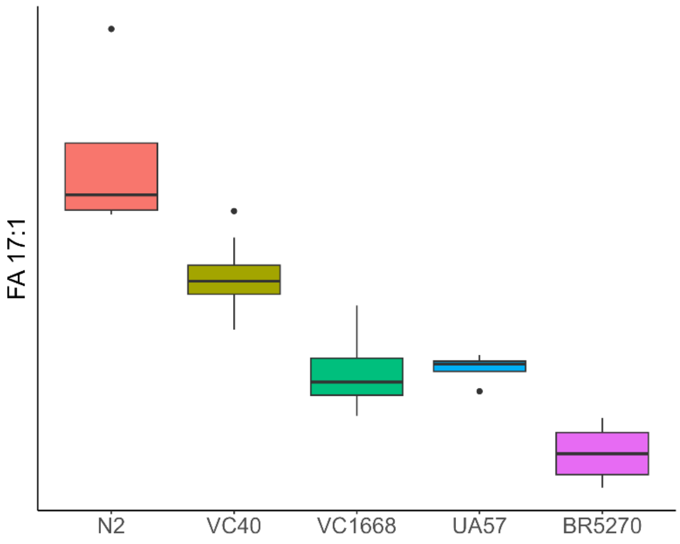

Pubchem CID:	3014063
InChIKey:	QSBYPNXLFMSGKH-UHFFFAOYSA-N
Title:	9-Heptadecenoic acid
Compound name:	FA 17:1
Name used:	FA 17:1
Annotation level:	3b
ANOVA FDR PValue:	1.222e-04
Fisher's LSD pairwise comparison:	N2 > VC40 - Pvalue: 7.016e-04<br>N2 > VC1668 - Pvalue: 4.248e-06<br>N2 > UA57 - Pvalue: 5.064e-06<br>N2 > BR5270 - Pvalue: 3.952e-08<br>VC40 > VC1668 - Pvalue: 0.0038<br>VC40 > UA57 - Pvalue: 0.0047<br>VC40 > BR5270 - Pvalue: 6.916e-06<br>VC1668 > BR5270 - Pvalue: 0.0244<br>UA57 > BR5270 - Pvalue: 0.0205
Type of analysis:	RPLCNEG scheme2 MSDial PublicMSP
Other analyses in which significative:	RPLCNEG scheme1 MSDial PublicMSP

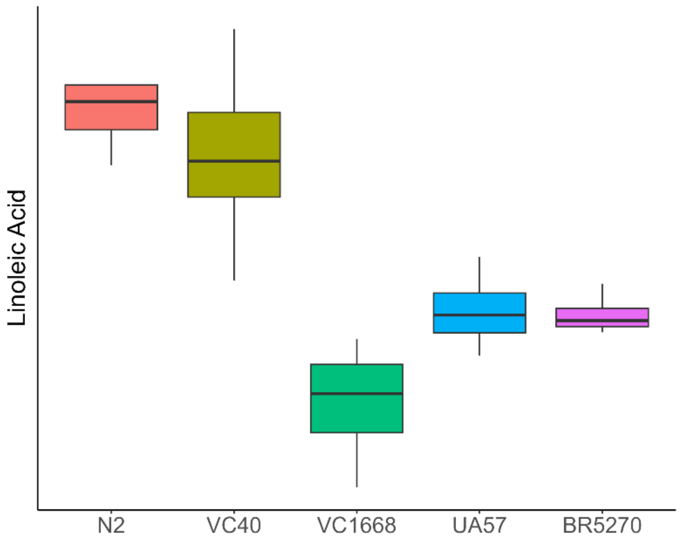

Pubchem CID:	5280450
InChIKey:	OYHQOLUKZRVURQ-HZJYTTRNSA-N
Title:	Linoleic Acid
Compound name:	(9Z,12Z)-octadeca-9,12-dienoic acid
Name used:	Linoleic Acid
Annotation level:	3b
ANOVA FDR PValue:	1.644e-04
Fisher's LSD pairwise comparison:	N2 > VC1668 - Pvalue: 2.364e-06<br>N2 > UA57 - Pvalue: 2.346e-04<br>N2 > BR5270 - Pvalue: 1.943e-04<br>VC40 > VC1668 - Pvalue: 2.445e-06<br>VC40 > UA57 - Pvalue: 5.143e-04<br>VC40 > BR5270 - Pvalue: 4.130e-04<br>UA57 > VC1668 - Pvalue: 0.0472
Type of analysis:	RPLCNEG scheme2 patRoon PubChemL
Other analyses in which significative:	RPLCNEG scheme2 patRoon WormJamE<br>RPLCNEG scheme1 patRoon PubChemL<br>RPLCNEG scheme1 patRoon WormJamE

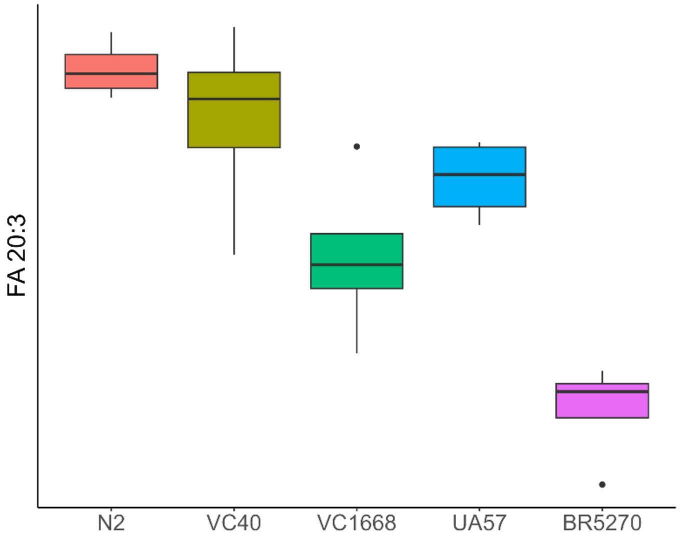

Pubchem CID:	3210
InChIKey:	AHANXAKGNAKFSK-UHFFFAOYSA-N
Title:	Eicosa-11,14,17-trienoic acid
Compound name:	FA 20:3
Name used:	FA 20:3
Annotation level:	3b
ANOVA FDR PValue:	1.820e-04
Fisher's LSD pairwise comparison:	N2 > VC1668 - Pvalue: 3.613e-04<br>N2 > UA57 - Pvalue: 0.0205<br>N2 > BR5270 - Pvalue: 2.282e-07<br>VC40 > VC1668 - Pvalue: 0.0013<br>VC40 > BR5270 - Pvalue: 2.367e-07<br>VC1668 > BR5270 - Pvalue: 0.0024<br>UA57 > BR5270 - Pvalue: 4.027e-05
Type of analysis:	RPLCNEG scheme1 MSDial PublicMSP
Other analyses in which significative:	RPLCNEG scheme2 MSDial PublicMSP

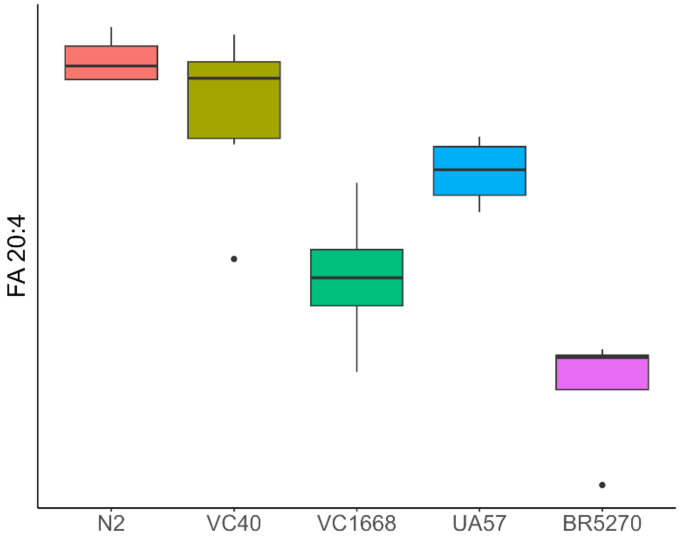

Pubchem CID:	3080584
InChIKey:	HQPCSDADVLFHHO-UHFFFAOYSA-N
Title:	Icosa-8,11,14,17-tetraenoic acid
Compound name:	FA 20:4
Name used:	FA 20:4
Annotation level:	3b
ANOVA FDR PValue:	2.334e-04
Fisher's LSD pairwise comparison:	N2 > VC1668 - Pvalue: 9.562e-05<br>N2 > UA57 - Pvalue: 0.0201<br>N2 > BR5270 - Pvalue: 5.285e-07<br>VC40 > VC1668 - Pvalue: 2.469e-04<br>VC40 > BR5270 - Pvalue: 5.681e-07<br>UA57 > VC1668 - Pvalue: 0.0279<br>VC1668 > BR5270 - Pvalue: 0.0228<br>UA57 > BR5270 - Pvalue: 1.092e-04
Type of analysis:	RPLCNEG scheme1 MSDial PublicMSP
Other analyses in which significative:	RPLCNEG scheme2 MSDial PublicMSP

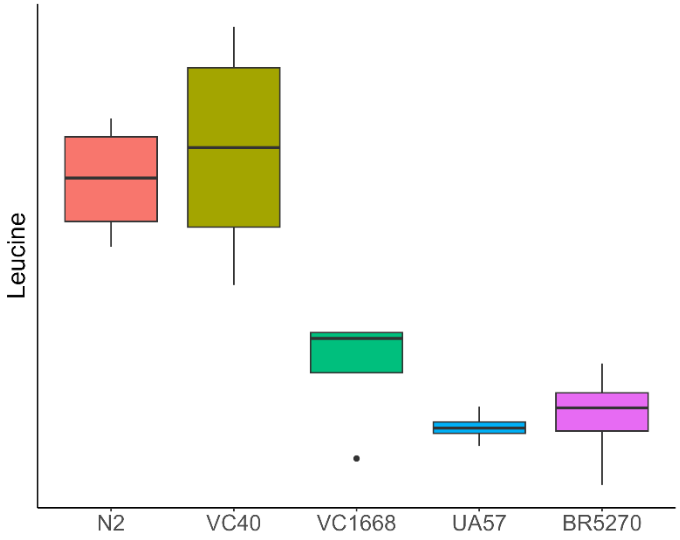

Pubchem CID:	6106
InChIKey:	ROHFNLRQFUQHCH-YFKPBYRVSA-N
Title:	Leucine
Compound name:	L-Leucine
Name used:	Leucine
Annotation level:	3b
ANOVA FDR PValue:	5.270e-04
Fisher's LSD pairwise comparison:	N2 > VC1668 - Pvalue: 0.0019<br>N2 > UA57 - Pvalue: 1.335e-04<br>N2 > BR5270 - Pvalue: 2.172e-04<br>VC40 > VC1668 - Pvalue: 1.059e-04<br>VC40 > UA57 - Pvalue: 5.707e-06<br>VC40 > BR5270 - Pvalue: 9.648e-06
Type of analysis:	RPLCNEG scheme1 MSDial PublicMSP

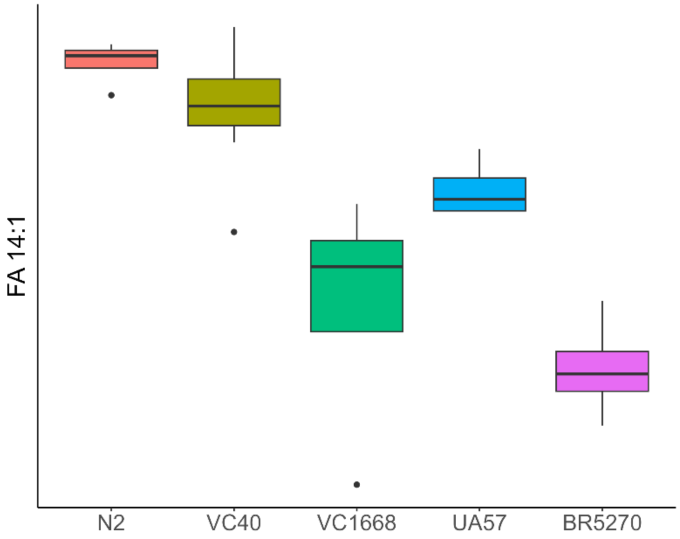

Pubchem CID:	68344
InChIKey:	YWWVWXASSLXJHU-UHFFFAOYSA-N
Title:	CID 68344
Compound name:	FA 14:1
Name used:	FA 14:1
Annotation level:	3b
ANOVA FDR PValue:	5.888e-04
Fisher's LSD pairwise comparison:	N2 > VC1668 - Pvalue: 5.491e-05<br>N2 > UA57 - Pvalue: 0.0142<br>N2 > BR5270 - Pvalue: 3.081e-06<br>VC40 > VC1668 - Pvalue: 1.247e-04<br>VC40 > BR5270 - Pvalue: 4.279e-06<br>UA57 > VC1668 - Pvalue: 0.0234<br>UA57 > BR5270 - Pvalue: 0.0012
Type of analysis:	RPLCNEG scheme1 MSDial PublicMSP

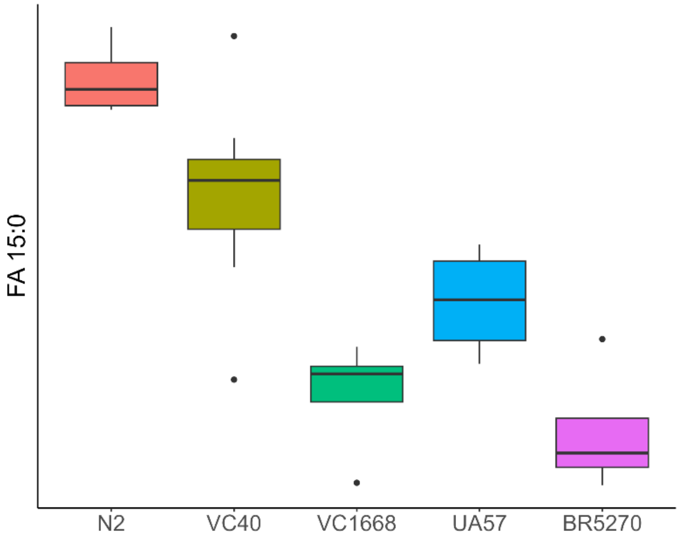

Pubchem CID:	13849
InChIKey:	WQEPLUUGTLDZJY-UHFFFAOYSA-N
Title:	Pentadecanoic Acid
Compound name:	FA 15:0
Name used:	FA 15:0
Annotation level:	3b
ANOVA FDR PValue:	7.142e-04
Fisher's LSD pairwise comparison:	N2 > VC40 - Pvalue: 0.0197<br>N2 > VC1668 - Pvalue: 9.760e-06<br>N2 > UA57 - Pvalue: 4.688e-04<br>N2 > BR5270 - Pvalue: 2.171e-06<br>VC40 > VC1668 - Pvalue: 3.563e-04<br>VC40 > UA57 - Pvalue: 0.0316<br>VC40 > BR5270 - Pvalue: 5.453e-05<br>UA57 > BR5270 - Pvalue: 0.0233
Type of analysis:	RPLCNEG scheme1 MSDial PublicMSP

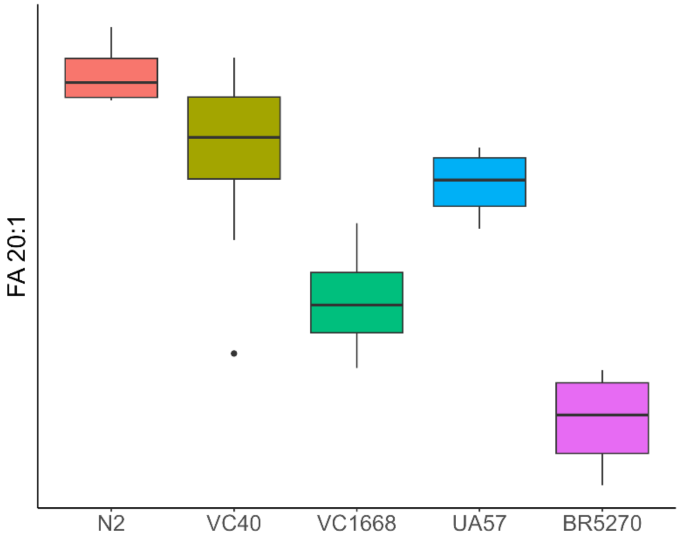

Pubchem CID:	142770
InChIKey:	BITHHVVYSMSWAG-UHFFFAOYSA-N
Title:	Eicos-11-enoic acid
Compound name:	FA 20:1
Name used:	FA 20:1
Annotation level:	3b
ANOVA FDR PValue:	8.410e-04
Fisher's LSD pairwise comparison:	N2 > VC1668 - Pvalue: 2.167e-04<br>N2 > UA57 - Pvalue: 0.0384<br>N2 > BR5270 - Pvalue: 1.193e-06<br>VC40 > VC1668 - Pvalue: 0.0036<br>VC40 > BR5270 - Pvalue: 7.034e-06<br>UA57 > VC1668 - Pvalue: 0.0310<br>VC1668 > BR5270 - Pvalue: 0.0254<br>UA57 > BR5270 - Pvalue: 1.374e-04
Type of analysis:	RPLCNEG scheme1 MSDial PublicMSP
Other analyses in which significative:	RPLCNEG scheme2 MSDial PublicMSP

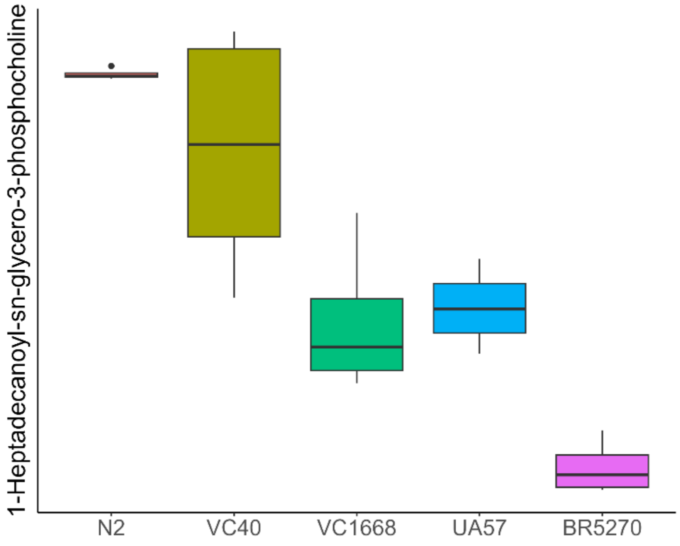

Pubchem CID:	24779463
InChIKey:	SRRQPVVYXBTRQK-XMMPIXPASA-N
Title:	1-Heptadecanoyl-sn-glycero-3-phosphoch
Compound name:	1-heptadecanoyl-2-hydroxy-sn-glycero-3-
Name used:	1-Heptadecanoyl-sn-glycero-3-phosphoch
Annotation level:	3b
ANOVA FDR PValue:	8.637e-04
Fisher's LSD pairwise comparison:	N2 > VC1668 - Pvalue: 2.331e-04<br>N2 > UA57 - Pvalue: 4.308e-04<br>N2 > BR5270 - Pvalue: 8.312e-07<br>VC40 > VC1668 - Pvalue: 0.0019<br>VC40 > UA57 - Pvalue: 0.0038<br>VC40 > BR5270 - Pvalue: 2.306e-06<br>VC1668 > BR5270 - Pvalue: 0.0161<br>UA57 > BR5270 - Pvalue: 0.0090
Type of analysis:	HILICPOS scheme1 MSDial PublicMSP

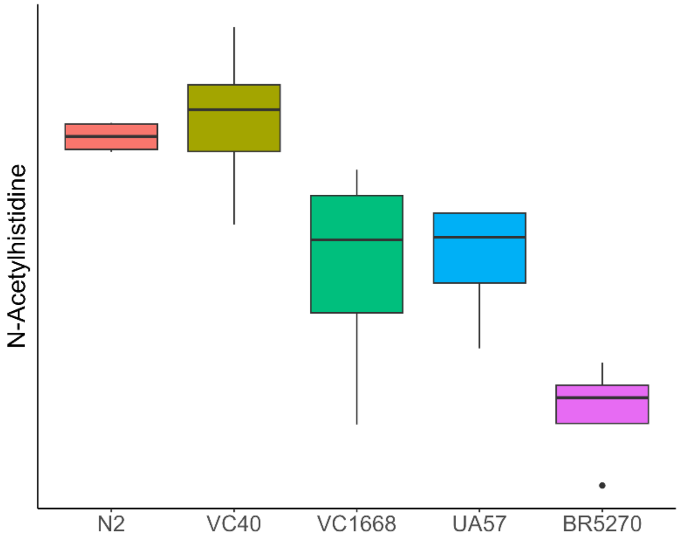

Pubchem CID:	75619
InChIKey:	KBOJOGQFRVVWBH-ZETCQYMHSA-N
Title:	N-Acetylhistidine
Compound name:	N-Acetylhistidine
Name used:	N-Acetylhistidine
Annotation level:	3b
ANOVA FDR PValue:	0.0011
Fisher's LSD pairwise comparison:	N2 > VC1668 - Pvalue: 0.0126<br>N2 > UA57 - Pvalue: 0.0194<br>N2 > BR5270 - Pvalue: 1.572e-05<br>VC40 > VC1668 - Pvalue: 0.0018<br>VC40 > UA57 - Pvalue: 0.0030<br>VC40 > BR5270 - Pvalue: 9.945e-07<br>VC1668 > BR5270 - Pvalue: 0.0077<br>UA57 > BR5270 - Pvalue: 0.0049
Type of analysis:	RPLCNEG scheme1 MSDial PublicMSP

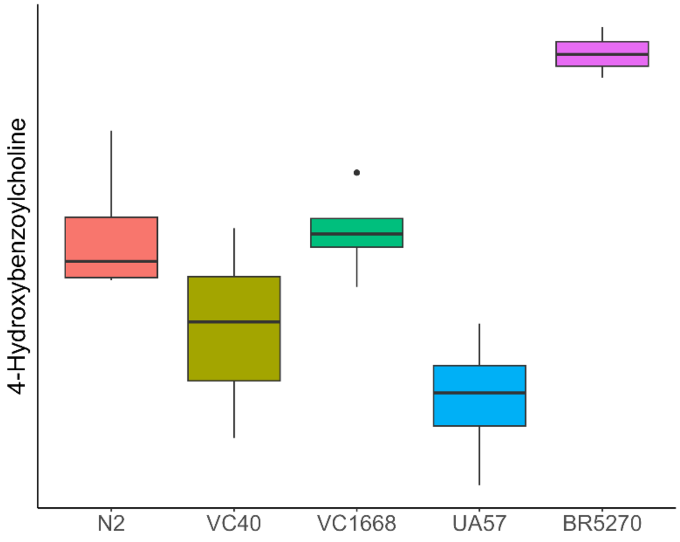

Pubchem CID:	151252
InChIKey:	BAPAICNRGIBFJT-UHFFFAOYSA-O
Title:	4-Hydroxybenzoylcholine
Compound name:	4-Hydroxybenzoylcholine
Name used:	4-Hydroxybenzoylcholine
Annotation level:	3b
ANOVA FDR PValue:	0.0014
Fisher's LSD pairwise comparison:	N2 > VC40 - Pvalue: 0.0198<br>N2 > UA57 - Pvalue: 0.0012<br>BR5270 > N2 - Pvalue: 5.597e-04<br>VC1668 > VC40 - Pvalue: 0.0181<br>BR5270 > VC40 - Pvalue: 6.086e-07<br>VC1668 > UA57 - Pvalue: 0.0011<br>BR5270 > VC1668 - Pvalue: 6.083e-04<br>BR5270 > UA57 - Pvalue: 1.909e-07
Type of analysis:	HILICPOS scheme2 MSDial PublicMSP

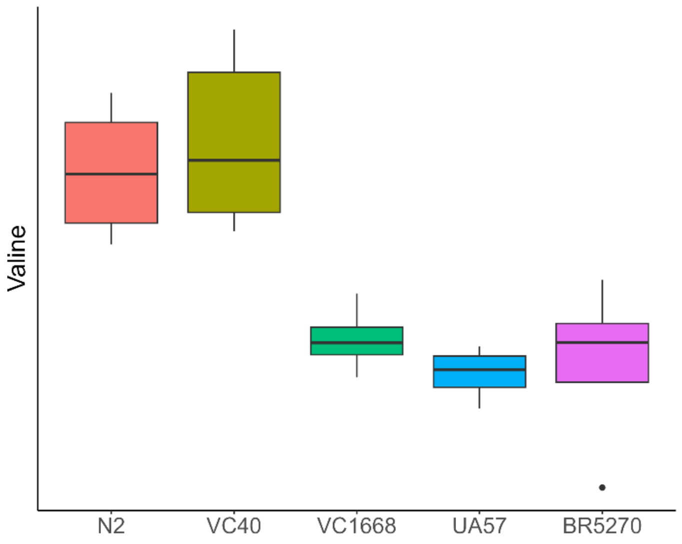

Pubchem CID:	6287
InChIKey:	KZSNJWFQEVHDMF-BYPYZUCNSA-N
Title:	Valine
Compound name:	L-Valine
Name used:	Valine
Annotation level:	3b
ANOVA FDR PValue:	0.0019
Fisher's LSD pairwise comparison:	N2 > VC1668 - Pvalue: 0.0029<br>N2 > UA57 - Pvalue: 5.811e-04<br>N2 > BR5270 - Pvalue: 9.447e-04<br>VC40 > VC1668 - Pvalue: 1.861e-04<br>VC40 > UA57 - Pvalue: 3.062e-05<br>VC40 > BR5270 - Pvalue: 5.261e-05
Type of analysis:	RPLCNEG scheme1 MSDial PublicMSP

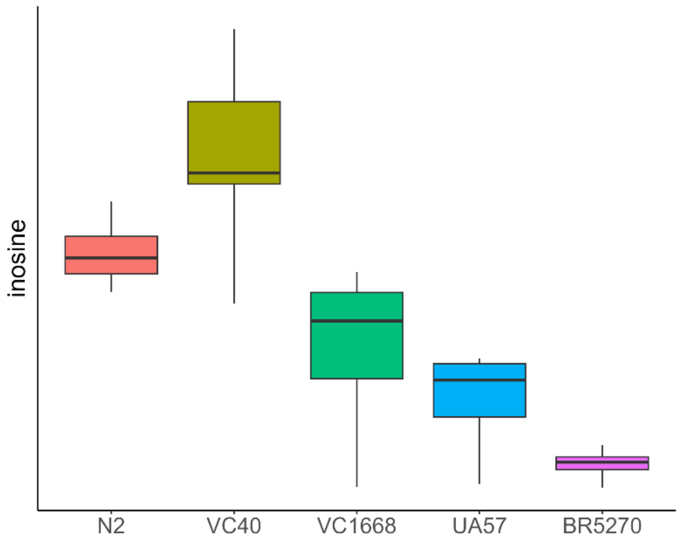

Pubchem CID:	6021
InChIKey:	UGQMRVRMYYASKQ-KQYNXXCUSA-N
Title:	CID 6021
Compound name:	inosine
Name used:	inosine
Annotation level:	3b
ANOVA FDR PValue:	0.0025
Fisher's LSD pairwise comparison:	VC40 > N2 - Pvalue: 0.0333<br>N2 > UA57 - Pvalue: 0.0074<br>N2 > BR5270 - Pvalue: 3.983e-04<br>VC40 > VC1668 - Pvalue: 2.040e-04<br>VC40 > UA57 - Pvalue: 1.510e-05<br>VC40 > BR5270 - Pvalue: 7.079e-07<br>VC1668 > BR5270 - Pvalue: 0.0325
Type of analysis:	HILICPOS scheme2 MSDial PublicMSP

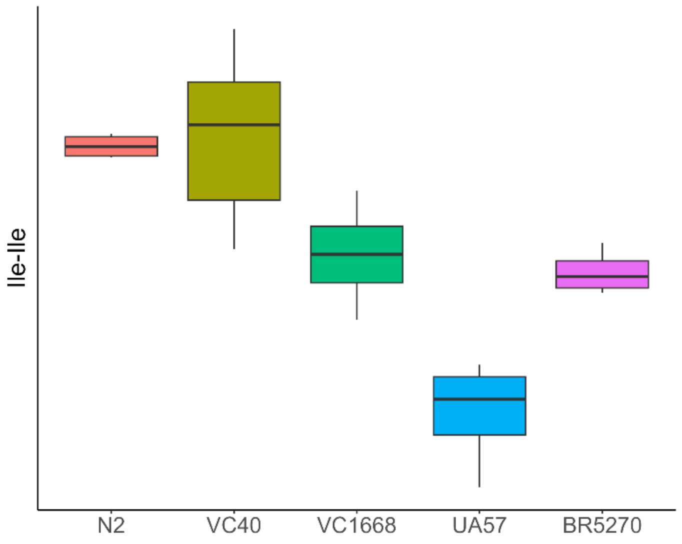

Pubchem CID:	13879965
InChIKey:	BCVIOZZGJNOEQS-UHFFFAOYSA-N
Title:	Ile-Ile
Compound name:	Isoleucylisoleucine
Name used:	Ile-Ile
Annotation level:	3b
ANOVA FDR PValue:	0.0026
Fisher's LSD pairwise comparison:	N2 > VC1668 - Pvalue: 0.0178<br>N2 > UA57 - Pvalue: 4.159e-06<br>N2 > BR5270 - Pvalue: 0.0072<br>VC40 > VC1668 - Pvalue: 0.0043<br>VC40 > UA57 - Pvalue: 3.607e-07<br>VC40 > BR5270 - Pvalue: 0.0015<br>VC1668 > UA57 - Pvalue: 0.0013<br>BR5270 > UA57 - Pvalue: 0.0033
Type of analysis:	HILICPOS scheme2 MSDial PublicMSP
Other analyses in which significative:	HILICPOS scheme1 MSDial PublicMSP

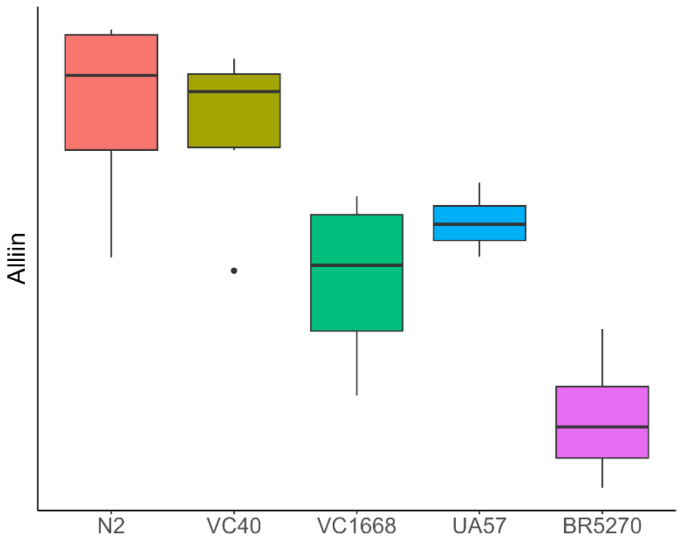

Pubchem CID:	138392130
InChIKey:	XUHLIQGRKRUKPH-JDLXGLENSA-N
Title:	2-amino-3-[(R)-prop-2-enylsulfinyl]propanc
Compound name:	Alliin
Name used:	Alliin
Annotation level:	3b
ANOVA FDR PValue:	0.0047
Fisher's LSD pairwise comparison:	N2 > VC1668 - Pvalue: 0.0049<br>N2 > UA57 - Pvalue: 0.0498<br>N2 > BR5270 - Pvalue: 1.585e-05<br>VC40 > VC1668 - Pvalue: 0.0025<br>VC40 > UA57 - Pvalue: 0.0391<br>VC40 > BR5270 - Pvalue: 3.730e-06<br>VC1668 > BR5270 - Pvalue: 0.0195<br>UA57 > BR5270 - Pvalue: 0.0017
Type of analysis:	HILICPOS scheme1 MSDial PublicMSP

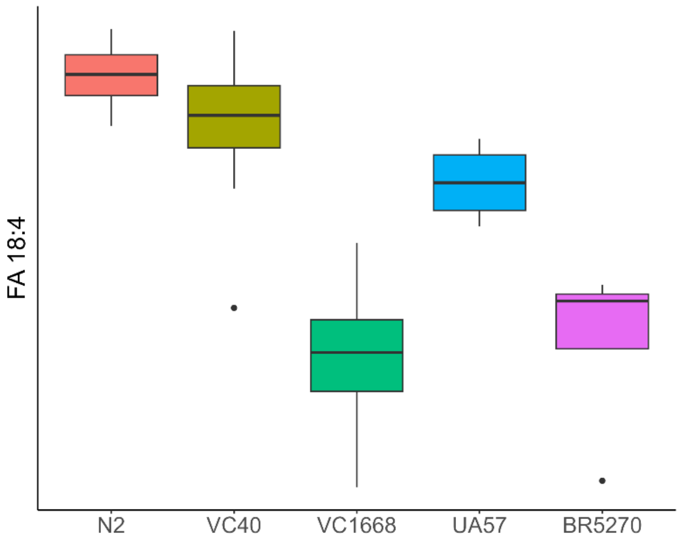

Pubchem CID:	163841
InChIKey:	JIWBIWFOSCKQMA-UHFFFAOYSA-N
Title:	6,9,12,15-Octadecatetraenoic acid
Compound name:	FA 18:4
Name used:	FA 18:4
Annotation level:	3b
ANOVA FDR PValue:	0.0049
Fisher's LSD pairwise comparison:	N2 > VC1668 - Pvalue: 6.217e-05<br>N2 > BR5270 - Pvalue: 1.227e-04<br>VC40 > VC1668 - Pvalue: 1.421e-04<br>VC40 > BR5270 - Pvalue: 3.146e-04<br>UA57 > VC1668 - Pvalue: 0.0049<br>UA57 > BR5270 - Pvalue: 0.0096
Type of analysis:	RPLCNEG scheme1 MSDial PublicMSP
Other analyses in which significative:	RPLCNEG scheme2 MSDial PublicMSP

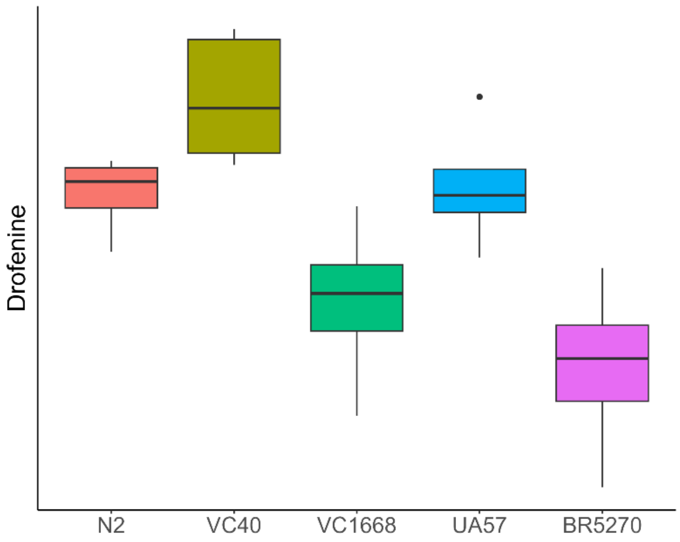

Pubchem CID:	3166
InChIKey:	AGJBLWCLQCKRJP-UHFFFAOYSA-N
Title:	Drofenine
Compound name:	Drofenine
Name used:	Drofenine
Annotation level:	3b
ANOVA FDR PValue:	0.0054
Fisher's LSD pairwise comparison:	VC40 > N2 - Pvalue: 0.0354<br>N2 > VC1668 - Pvalue: 0.0366<br>N2 > BR5270 - Pvalue: 0.0019<br>VC40 > VC1668 - Pvalue: 1.085e-04<br>VC40 > BR5270 - Pvalue: 3.593e-06<br>UA57 > VC1668 - Pvalue: 0.0262<br>UA57 > BR5270 - Pvalue: 0.0013
Type of analysis:	HILICPOS scheme1 MSDial PublicMSP

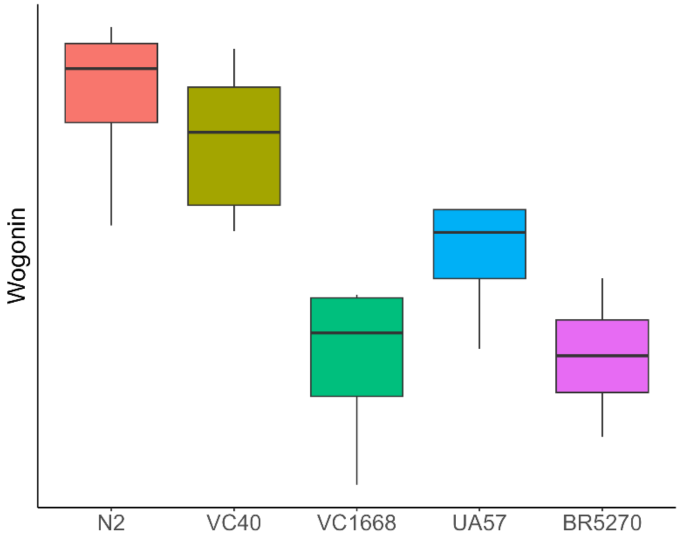

Pubchem CID:	5281703
InChIKey:	XLTFNNCXVBYBSX-UHFFFAOYSA-N
Title:	Wogonin
Compound name:	Wogonin
Name used:	Wogonin
Annotation level:	3b
ANOVA FDR PValue:	0.0079
Fisher's LSD pairwise comparison:	N2 > VC1668 - Pvalue: 9.290e-05<br>N2 > UA57 - Pvalue: 0.0081<br>N2 > BR5270 - Pvalue: 1.131e-04<br>VC40 > VC1668 - Pvalue: 1.349e-04<br>VC40 > UA57 - Pvalue: 0.0223<br>VC40 > BR5270 - Pvalue: 1.695e-04
Type of analysis:	HILICPOS scheme1 MSDial PublicMSP
Other analyses in which significative:	HILICPOS scheme2 MSDial PublicMSP

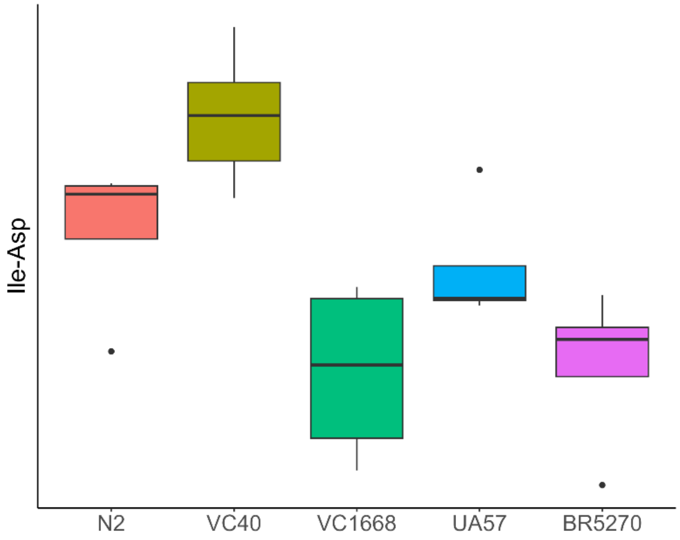

Pubchem CID:	14304554
InChIKey:	WKXVAXOSIPTXEC-UHFFFAOYSA-N
Title:	Ile-Asp
Compound name:	Isoleucylaspartate
Name used:	Ile-Asp
Annotation level:	3b
ANOVA FDR PValue:	0.0149
Fisher's LSD pairwise comparison:	VC40 > N2 - Pvalue: 0.0194<br>N2 > VC1668 - Pvalue: 0.0136<br>N2 > BR5270 - Pvalue: 0.0182<br>VC40 > VC1668 - Pvalue: 1.725e-05<br>VC40 > UA57 - Pvalue: 0.0031<br>VC40 > BR5270 - Pvalue: 2.422e-05
Type of analysis:	HILICPOS scheme2 MSDial PublicMSP

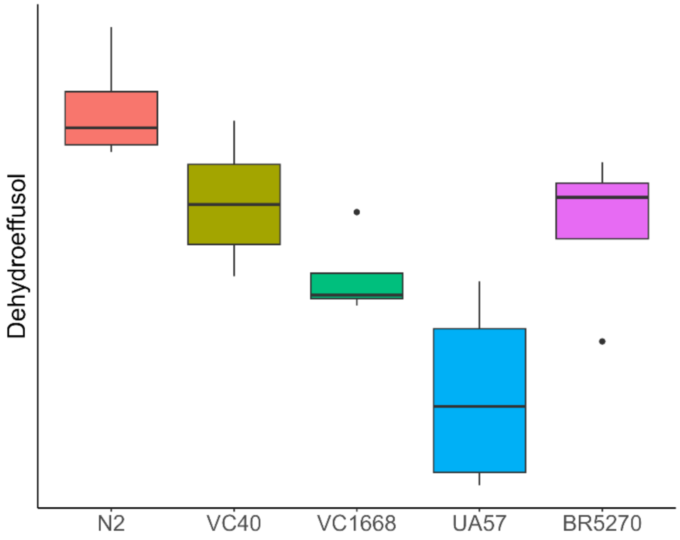

Pubchem CID:	5316810
InChIKey:	GSSPKCPIRDPBQE-UHFFFAOYSA-N
Title:	5-Ethenyl-1-methylphenanthrene-2,7-diol
Compound name:	Dehydroeffusol
Name used:	Dehydroeffusol
Annotation level:	3b
ANOVA FDR PValue:	0.0152
Fisher's LSD pairwise comparison:	N2 > VC40 - Pvalue: 0.0330<br>N2 > VC1668 - Pvalue: 0.0020<br>N2 > UA57 - Pvalue: 7.204e-06<br>N2 > BR5270 - Pvalue: 0.0231<br>VC40 > UA57 - Pvalue: 1.396e-04<br>VC1668 > UA57 - Pvalue: 0.0211<br>BR5270 > UA57 - Pvalue: 0.0018
Type of analysis:	HILICPOS scheme1 MSDial PublicMSP

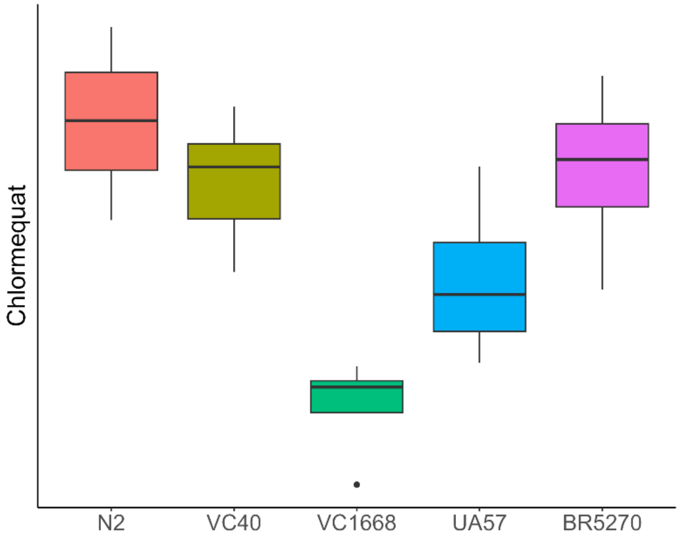

Pubchem CID:	13837
InChIKey:	JUZXDNPBRPUIOR-UHFFFAOYSA-N
Title:	Chlormequat
Compound name:	Chlormequat
Name used:	Chlormequat
Annotation level:	3b
ANOVA FDR PValue:	0.0172
Fisher's LSD pairwise comparison:	N2 > VC1668 - Pvalue: 2.319e-05<br>N2 > UA57 - Pvalue: 0.0062<br>VC40 > VC1668 - Pvalue: 7.151e-05<br>VC40 > UA57 - Pvalue: 0.0416<br>UA57 > VC1668 - Pvalue: 0.0227<br>BR5270 > VC1668 - Pvalue: 1.961e-04<br>BR5270 > UA57 - Pvalue: 0.0474
Type of analysis:	HILICPOS scheme1 MSDial PublicMSP

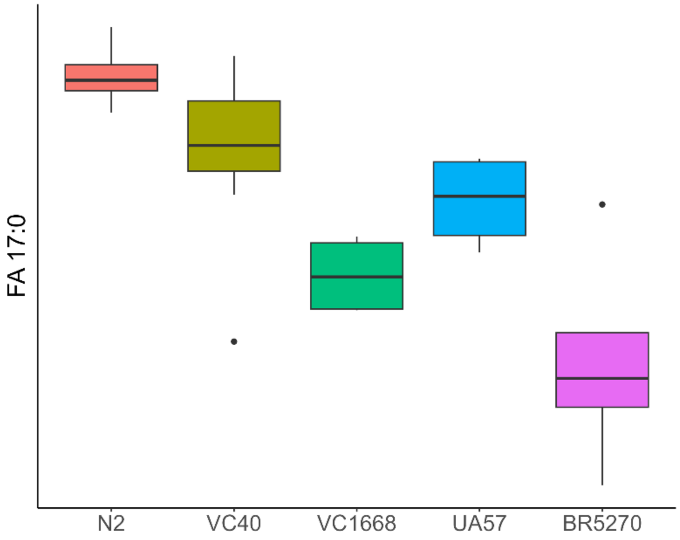

Pubchem CID:	10465
InChIKey:	KEMQGTRYUADPNZ-UHFFFAOYSA-N
Title:	Heptadecanoic acid
Compound name:	FA 17:0
Name used:	FA 17:0
Annotation level:	3b
ANOVA FDR PValue:	0.0181
Fisher's LSD pairwise comparison:	N2 > VC1668 - Pvalue: 0.0014<br>N2 > UA57 - Pvalue: 0.0298<br>N2 > BR5270 - Pvalue: 3.676e-05<br>VC40 > VC1668 - Pvalue: 0.0181<br>VC40 > BR5270 - Pvalue: 2.744e-04<br>UA57 > BR5270 - Pvalue: 0.0074
Type of analysis:	RPLCNEG scheme1 MSDial PublicMSP
Other analyses in which significative:	RPLCNEG scheme2 MSDial PublicMSP

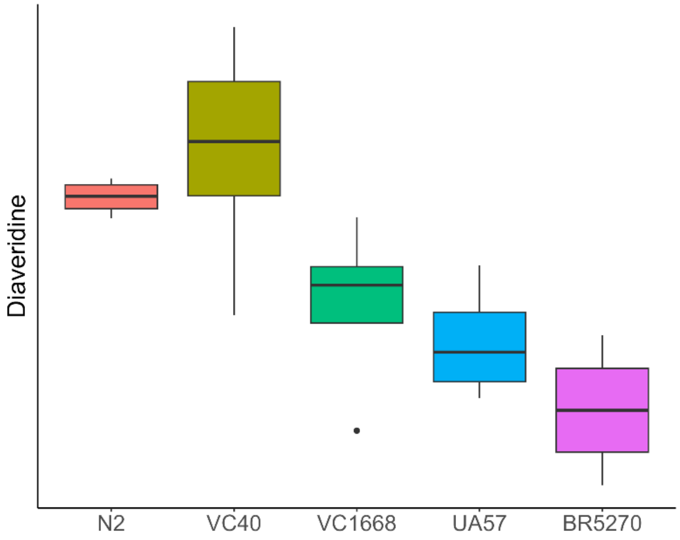

Pubchem CID:	21453
InChIKey:	LDBTVAXGKYIFHO-UHFFFAOYSA-N
Title:	Diaveridine
Compound name:	Diaveridine
Name used:	Diaveridine
Annotation level:	3b
ANOVA FDR PValue:	0.0257
Fisher's LSD pairwise comparison:	N2 > UA57 - Pvalue: 0.0157<br>N2 > BR5270 - Pvalue: 9.523e-04<br>VC40 > VC1668 - Pvalue: 0.0032<br>VC40 > UA57 - Pvalue: 5.247e-04<br>VC40 > BR5270 - Pvalue: 2.070e-05
Type of analysis:	HILICPOS scheme2 MSDial PublicMSP

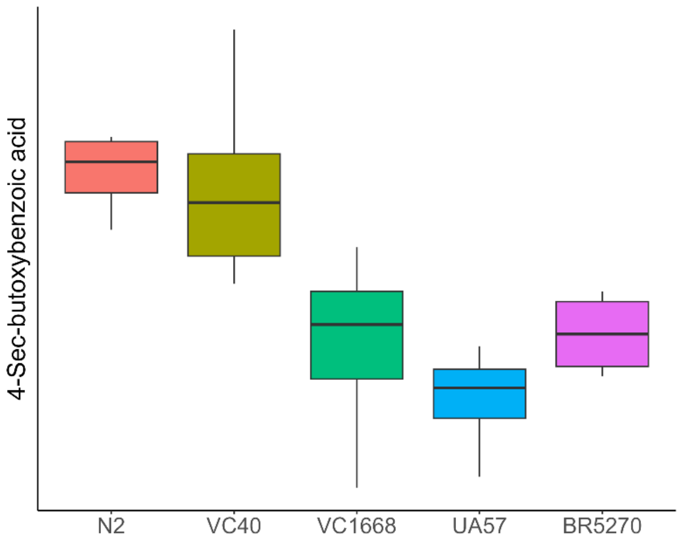

Pubchem CID:	5132695
InChIKey:	LCMFDJQAHNDLEI-UHFFFAOYSA-N
Title:	4-Sec-butoxybenzoic acid
Compound name:	4-(sec-butoxy)benzoic acid
Name used:	4-Sec-butoxybenzoic acid
Annotation level:	3b
ANOVA FDR PValue:	0.0268
Fisher's LSD pairwise comparison:	N2 > VC1668 - Pvalue: 0.0032<br>N2 > UA57 - Pvalue: 2.960e-04<br>N2 > BR5270 - Pvalue: 0.0054<br>VC40 > VC1668 - Pvalue: 0.0025<br>VC40 > UA57 - Pvalue: 1.603e-04<br>VC40 > BR5270 - Pvalue: 0.0046
Type of analysis:	HILICPOS scheme1 MSDial PublicMSP

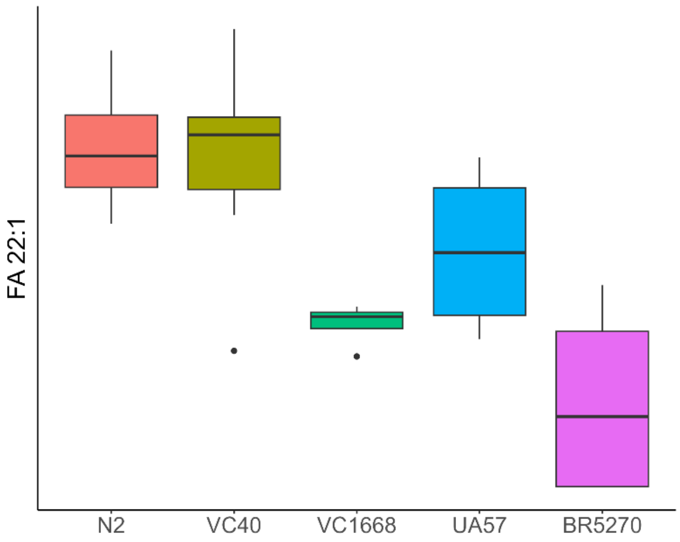

Pubchem CID:	8216
InChIKey:	DPUOLQHDNGRHBS-UHFFFAOYSA-N
Title:	13-Docosenoic acid
Compound name:	FA 22:1
Name used:	FA 22:1
Annotation level:	3b
ANOVA FDR PValue:	0.0334
Fisher's LSD pairwise comparison:	N2 > VC1668 - Pvalue: 0.0077<br>N2 > BR5270 - Pvalue: 4.047e-04<br>VC40 > VC1668 - Pvalue: 0.0046<br>VC40 > BR5270 - Pvalue: 1.519e-04<br>UA57 > BR5270 - Pvalue: 0.0202
Type of analysis:	RPLCNEG scheme1 MSDial PublicMSP

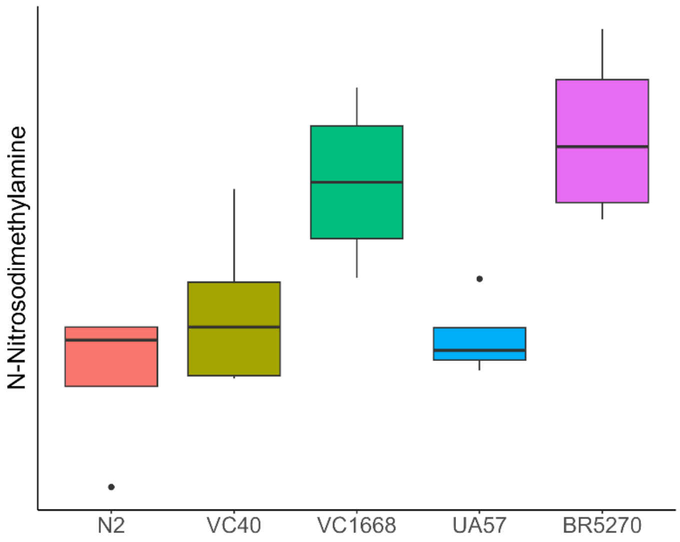

Pubchem CID:	6124
InChIKey:	UMFJAHHVKNCGLG-UHFFFAOYSA-N
Title:	N-Nitrosodimethylamine
Compound name:	N-Nitrosodimethylamine (NDMA)
Name used:	N-Nitrosodimethylamine
Annotation level:	3b
ANOVA FDR PValue:	0.0395
Fisher's LSD pairwise comparison:	VC1668 > N2 - Pvalue: 0.0015<br>BR5270 > N2 - Pvalue: 1.870e-04<br>VC1668 > VC40 - Pvalue: 0.0078<br>BR5270 > VC40 - Pvalue: 7.174e-04<br>VC1668 > UA57 - Pvalue: 0.0072<br>BR5270 > UA57 - Pvalue: 9.178e-04
Type of analysis:	HILICPOS scheme1 MSDial PublicMSP

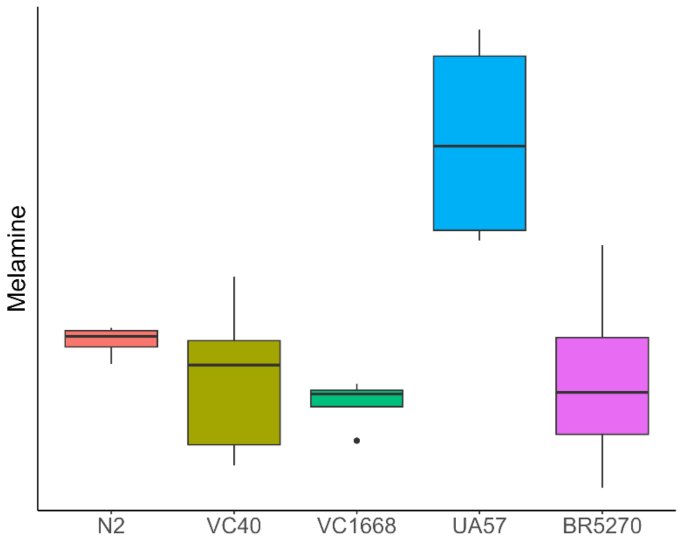

Pubchem CID:	7955
InChIKey:	JDSHMPZPIAZGSV-UHFFFAOYSA-N
Title:	Melamine
Compound name:	Melamine
Name used:	Melamine
Annotation level:	3b
ANOVA FDR PValue:	0.0401
Fisher's LSD pairwise comparison:	UA57 > N2 - Pvalue: 9.685e-04<br>UA57 > VC40 - Pvalue: 3.562e-05<br>UA57 > VC1668 - Pvalue: 6.326e-05<br>UA57 > BR5270 - Pvalue: 1.779e-04
Type of analysis:	HILICPOS scheme2 MSDial PublicMSP

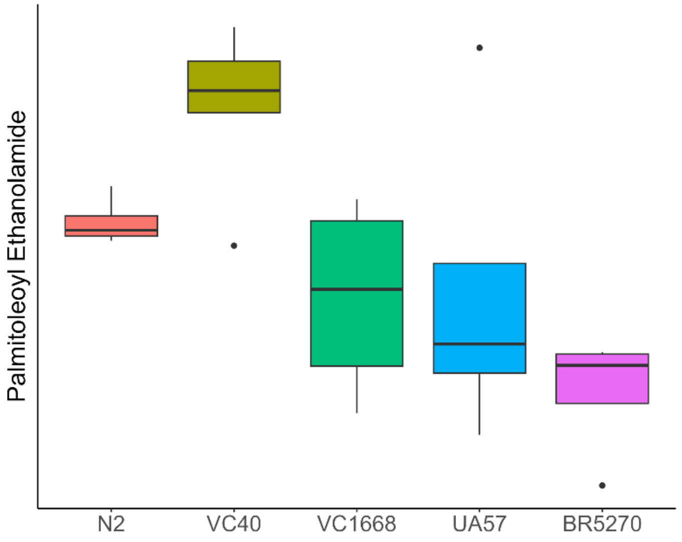

Pubchem CID:	9835868
InChIKey:	WFRLANWAASSSFV-FPLPWBNLSA-N
Title:	Palmitoleoyl Ethanolamide
Compound name:	Palmitoleoyl Ethanolamide
Name used:	Palmitoleoyl Ethanolamide
Annotation level:	3b
ANOVA FDR PValue:	0.0464
Fisher's LSD pairwise comparison:	VC40 > N2 - Pvalue: 0.0426<br>N2 > BR5270 - Pvalue: 0.0171<br>VC40 > VC1668 - Pvalue: 0.0023<br>VC40 > UA57 - Pvalue: 0.0028<br>VC40 > BR5270 - Pvalue: 5.191e-05
Type of analysis:	HILICPOS scheme1 MSDial PublicMSP
Other analyses in which significative:	HILICPOS scheme1 MSDial WormJamEx

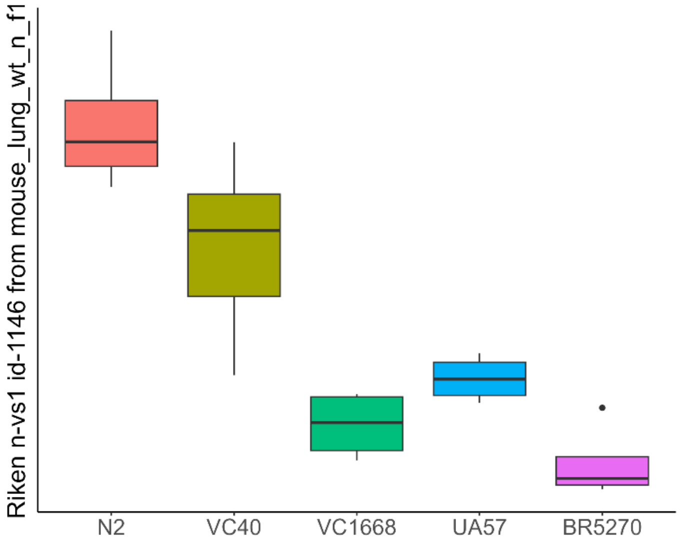

Pubchem CID:	null
InChIKey:	null
Title:	NA
Compound name:	RIKEN N-VS1 ID-1146 from Mouse_Lung_
Name used:	Riken n-vs1 id-1146 from mouse_lung_wt_
Annotation level:	3c
ANOVA FDR PValue:	1.087e-04
Fisher's LSD pairwise comparison:	N2 > VC40 - Pvalue: 0.0030<br>N2 > VC1668 - Pvalue: 6.633e-07<br>N2 > UA57 - Pvalue: 6.480e-06<br>N2 > BR5270 - Pvalue: 1.130e-07<br>VC40 > VC1668 - Pvalue: 7.902e-05<br>VC40 > UA57 - Pvalue: 0.0015<br>VC40 > BR5270 - Pvalue: 7.523e-06
Type of analysis:	RPLCNEG scheme2 MSDial PublicMSP
Other analyses in which significative:	RPLCNEG scheme1 MSDial PublicMSP
